# Data set of interactomes and metabolic pathways of proteins differentially expressed in brains with Alzheimer׳s disease

**DOI:** 10.1016/j.dib.2016.04.071

**Published:** 2016-05-06

**Authors:** Benito Minjarez, Karla Grisel Calderón-González, Ma Luz Valero Rustarazo, María Esther Herrera-Aguirre, María Luisa Labra-Barrios, Diego E. Rincon-Limas, Manuel M. Sánchez del Pino, Raul Mena, Juan Pedro Luna-Arias

**Affiliations:** a Departamento de Biología Celular, Centro de Investigación y de Estudios Avanzados del Instituto Politécnico Nacional (Cinvestav-IPN), Av. Instituto Politécnico Nacional 2508, Col. San Pedro Zacatenco, Gustavo A. Madero, C.P. 07360 Ciudad de México, México; b Departamento de Biología Celular y Molecular, Centro Universitario de Ciencias Biológicas y Agropecuarias (CUCBA), Universidad de Guadalajara, Camino Ramón Padilla Sánchez No. 2100, Nextipac, Zapopan, Jalisco, México; c Unidad de Proteómica, Centro de Investigación Príncipe Felipe, C/Rambla del Saler 16, 46012 Valencia, España; d Secció de Proteómica, Servei Central de Suport a la Investigació Experimental (SCSIE), Universitat de València, C/Doctor Moliner 50, 46100 Burjassot, València, España; e Departments of Neurology and Neuroscience, McKnight Brain Institute, University of Florida, Gainesville, FL 32611, USA; f Departament de Bioquímica i Biologia Molecular, Facultat de Ciències Biològiques, Universitat de València, C/ Doctor Moliner 50, 46100 Burjassot, València, España; g Departamento de Fisiología, Biofísica y Neurociencias, Cinvestav-IPN, Av. Instituto Politécnico Nacional 2508, Col. San Pedro Zacatenco, Gustavo A. Madero, C.P. 07360 Ciudad de México, México

## Abstract

Alzheimer׳s disease is one of the main causes of dementia in the elderly and its frequency is on the rise worldwide. It is considered the result of complex interactions between genetic and environmental factors, being many of them unknown. Therefore, there is a dire necessity for the identification of novel molecular players for the understanding of this disease. In this data article we determined the protein expression profiles of whole protein extracts from cortex regions of brains from patients with Alzheimer׳s disease in comparison to a normal brain. We identified 721 iTRAQ-labeled polypeptides with more than 95% in confidence. We analyzed all proteins that changed in their expression level and located them in the KEGG metabolic pathways, as well as in the mitochondrial complexes of the electron transport chain and ATP synthase. In addition, we analyzed the over- and sub-expressed polypeptides through IPA software, specifically Core I and Biomarkers I modules. Data in this article is related to the research article “Identification of proteins that are differentially expressed in brains with Alzheimer’s disease using iTRAQ labeling and tandem mass spectrometry” (Minjarez et al., 2016) [1].

**Specifications Table**

**Table:** 

Subject area	Cell Biology
More specific subject area	Alzheimer׳s disease
Type of data	Tables, figures
How data was acquired	Isobaric labeling, preparative isoelectrofocusing, reverse phase chromatography, and tandem mass spectrometry using an AB SCIEX high performance hybrid quadrupole time-of-flight QSTAR ESI XL Hybrid LC/MS/MS Mass Spectrometer System
Data format	Analyzed and filtered
Experimental factors	Samples were reduced, alkylated and digested with 50 mM TCEP, 200 mM MMTS, and sequencing grade Trypsin, respectively, and iTRAQ-labeled according to the protocol described in the manual of the iTRAQ 8-plex kit, with minor modifications
Experimental features	Peptides were labeled, pooled and separated by isoelectrofocusing on a non-linear pH 3-10 gradient. Strips were divided in sections, and peptides extracted were chromatographed in a C18 column using several ACN linear gradients and analyzed by MS/MS. Identified proteins were grouped and classified with IPA and KEGG software, as well as analyzed in IntAct and BioGRID databases
Data source location	Mexico City, Mexico
Data accessibility	Data is within this article

**Value of the data**

• Data provide the whole list of proteins identified in brains with Alzheimer׳s disease and their expression levels in comparison to a normal brain.
• Data provide information of over- and sub-expressed polypeptides found in common in all Alzheimer׳s disease brains in relation to diseases and biofunctions in which they might be involved according to IPA database.
• Data can be used in further studies to gain insight into the different affected pathways or networks, or into the role of each identified protein using cellular or whole organism models of Alzheimer׳s disease.
• Data provide insight of putative biomarkers found in Alzheimer׳s disease brains and open the possibility to explore their expression level in a bigger number of brains with Alzheimer׳s disease in comparison to normal brains to validate them as possible biomarkers, as well as in serum of CSF samples.

## 1 Data

**Fig. 1:**
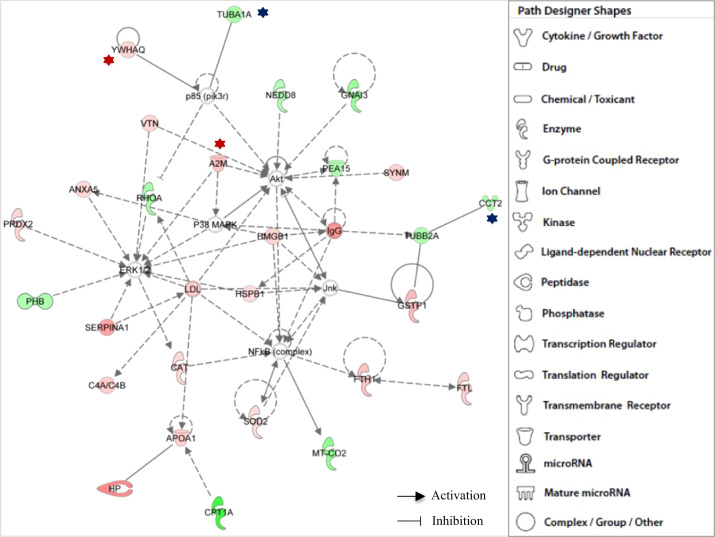
IPA functional network of differentially expressed proteins found in all brains with Alzheimer׳s disease through IPA Core I analyses related with (i) Free Radical Scavenging, (ii) Neurological Disease, (iii) Cancer, and (iv) Cell Death and Survival. Different shapes, indicating the functional class to which they belong, represent proteins. Molecules in red are overexpressed polypeptides, whilst those green molecules correspond to subexpressed proteins. IPA incorporated molecules in grey, which are not specified, into the networks through relationships with other molecules. Molecular relationships between polypeptides are indicated with lines. A continuous line illustrates a direct interaction and a dotted line is used for indirect interactions. Proteins, whose expression is age-dependent, are labeled with a star (red and blue for over- and sub-expressed, respectively).

**Fig. 2:**
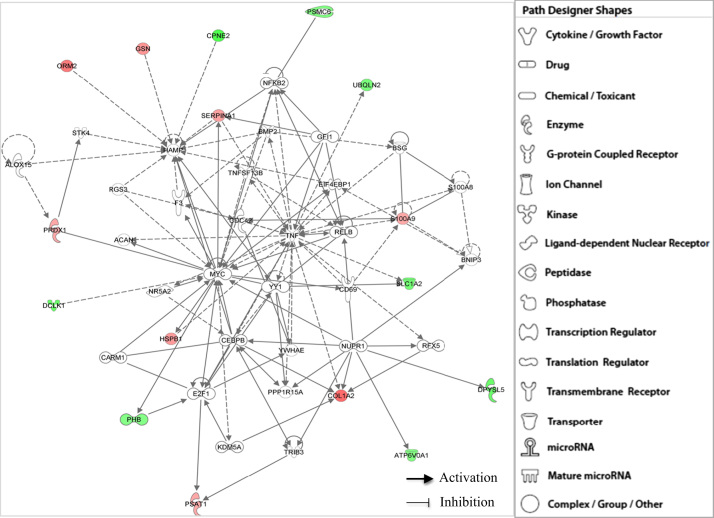
IPA functional network of differentially expressed proteins found in all brains with Alzheimer׳s disease through core I analyses related with (i) Cell Death and Survival, (ii) Cellular Movement, (iii) Cancer, (iv) Cellular Growth and Proliferation, (v) Tissue Morphology, (vi) Hematological System Development and Function, (vii) Immune Cell Trafficking, and (viii) Organ Morphology. Different shapes, indicating the functional class to which they belong, represent proteins. Molecules in red are overexpressed polypeptides, whilst those green molecules correspond to subexpressed proteins. IPA incorporated molecules in grey, which are not specified, into the networks through relationships with other molecules. Molecular relationships between polypeptides are indicated with lines. A continuous line illustrates a direct interaction and a dotted line is used for indirect interactions.

**Fig. 3:**
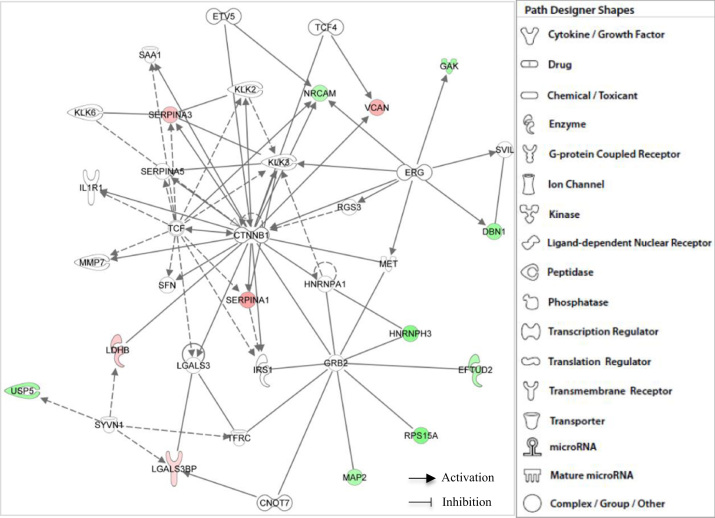
IPA functional network of differentially expressed proteins found in all brains with Alzheimer׳s disease through core I analyses related with (i) Cellular Movement, (ii) Cellular Growth and Proliferation, (iii) Cell Death and Survival, (iv) Cancer, and (v) Gastrointestinal Disease. Different shapes, indicating the functional class to which they belong, represent proteins. Molecules in red are overexpressed polypeptides, whilst those green molecules correspond to subexpressed proteins. Molecules in grey, which are not specified, were incorporated into the networks by IPA through relationships with other molecules. Molecular relationships between polypeptides are indicated with lines. A continuous line illustrates a direct interaction and a dotted line is used for indirect interactions.

**Fig. 4:**
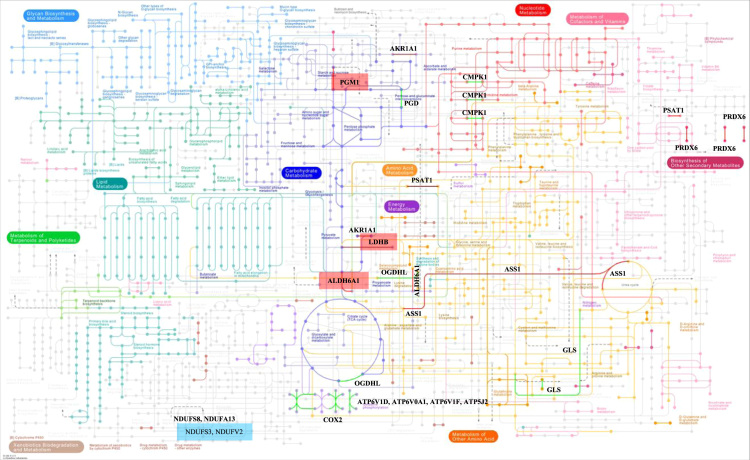
Global view of the metabolic pathways of the KEGG database that were affected in brains with Alzheimer׳s disease. The overexpressed proteins are represented in red color and the subexpressed polypeptides in green. Those proteins whose expression is age-dependent are in red (overexpressed) and blue (subexpressed) rectangles (see Table 1 and 2 in [1]).

**Fig. 5:**
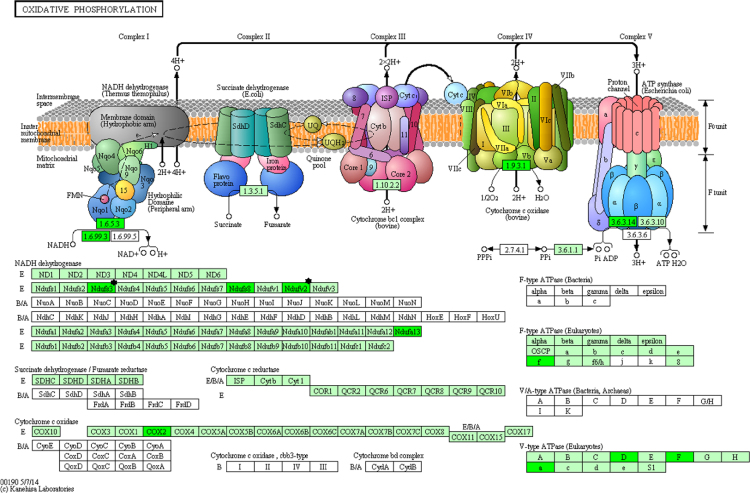
Localization of subexpressed proteins in the oxidative phosphorylation process obtained from the KEGG database. The localization of the subexpressed polypeptides in each complex of the respiratory chain is indicated in brilliant green color. We also show in brilliant green the subunits of the V-ATPase that were found subexpressed. Proteins, whose expression is age-dependent, are labeled with a black star (see Table 2 in [1]).

**Fig. 6:**
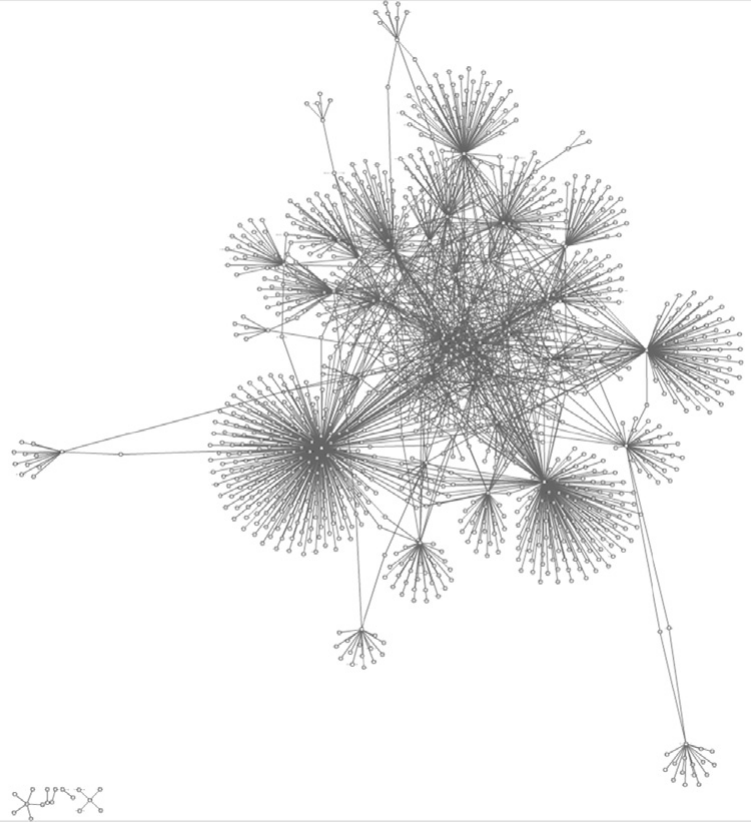
Interactions maps determined in the IntAct Molecular Interaction Database for the set of overexpressed proteins in brains with Alzheimer׳s disease (see Table 1 in [1] for data).

**Fig. 7:**
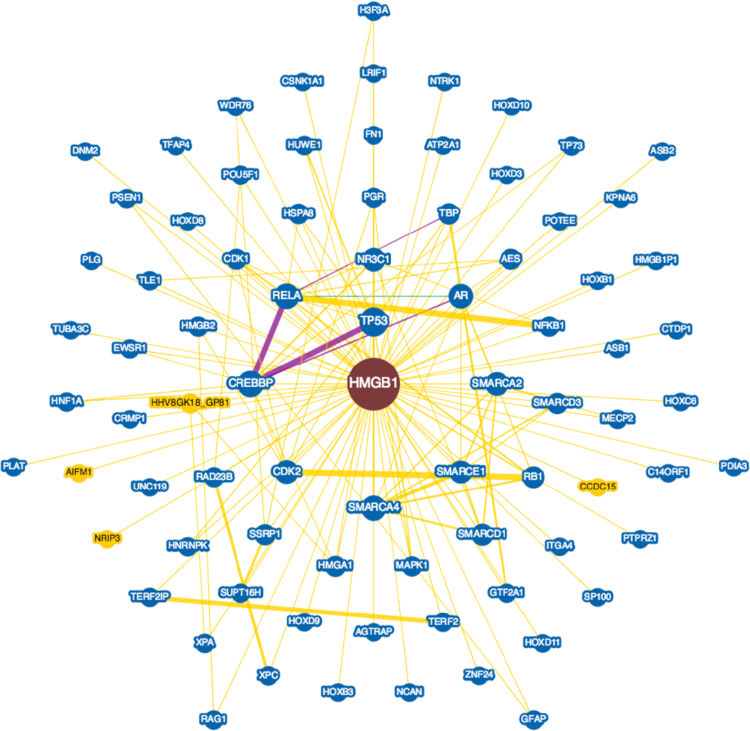
Interactions experimentally determined for the overexpressed High Mobility Group Box 1 protein (*HMGB1*, UniProtKB ID P09429) in brains with Alzheimer׳s disease in BioGRID database.

**Fig. 8:**
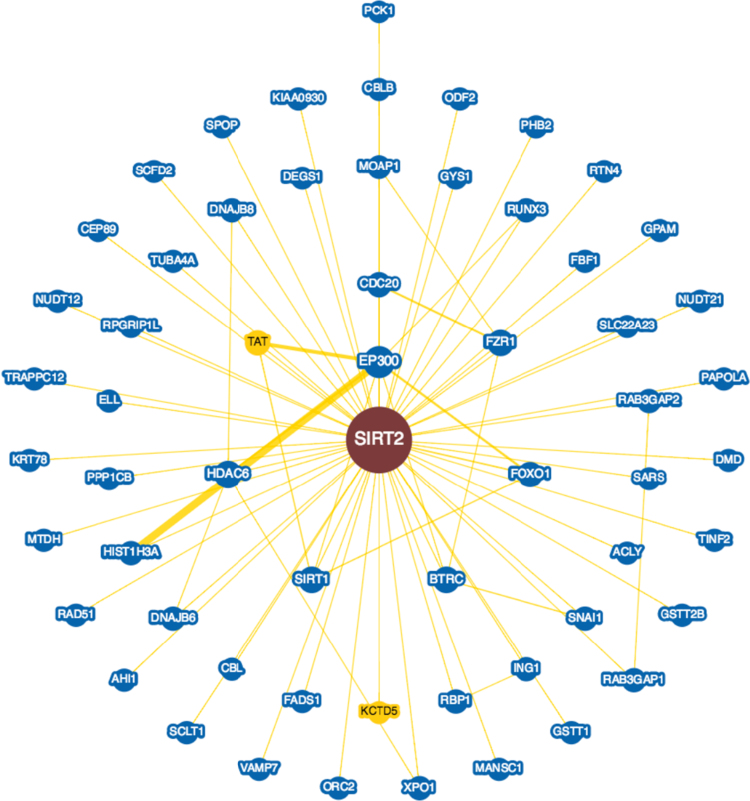
Interactions experimentally determined for the overexpressed NAD-dependent deacetylase sirtuin-2 protein (*SIRT2*, UniProtKB ID Q8IXJ6) in brains with Alzheimer׳s disease in BioGRID database.

**Fig. 9:**
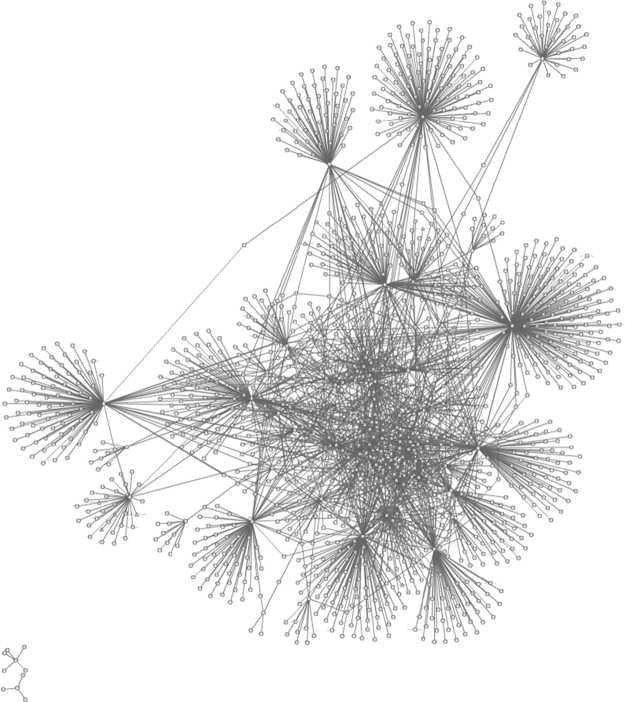
Interactions maps determined in the IntAct Molecular Interaction Database for the set of subexpressed proteins in brains with Alzheimer׳s disease (see Table 2 in [1] for data).

**Fig. 10:**
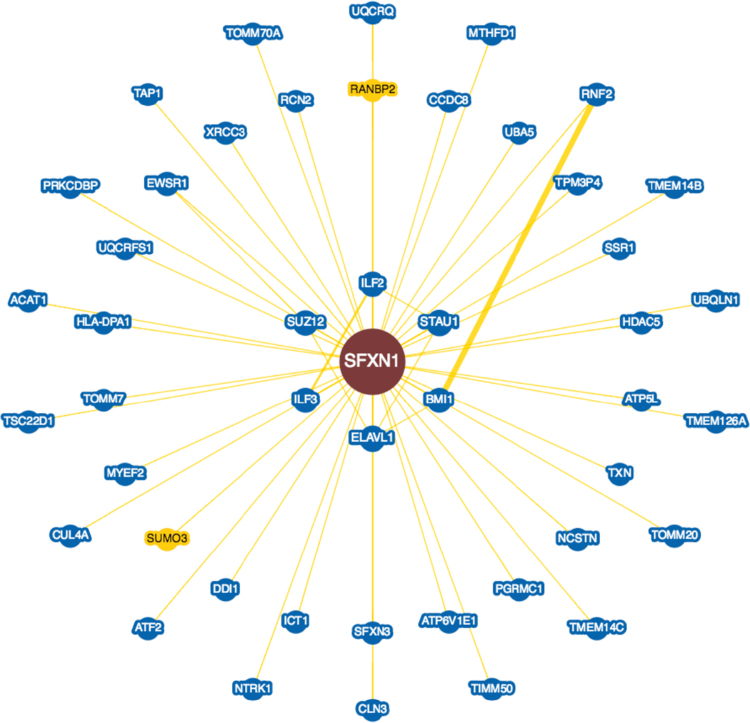
Interactions experimentally determined for the subexpressed Sideroflexin-1 protein (*SFXN1*, UniProtKB ID Q9H9B4) in brains with Alzheimer׳s disease in BioGRID database.

**Fig. 11:**
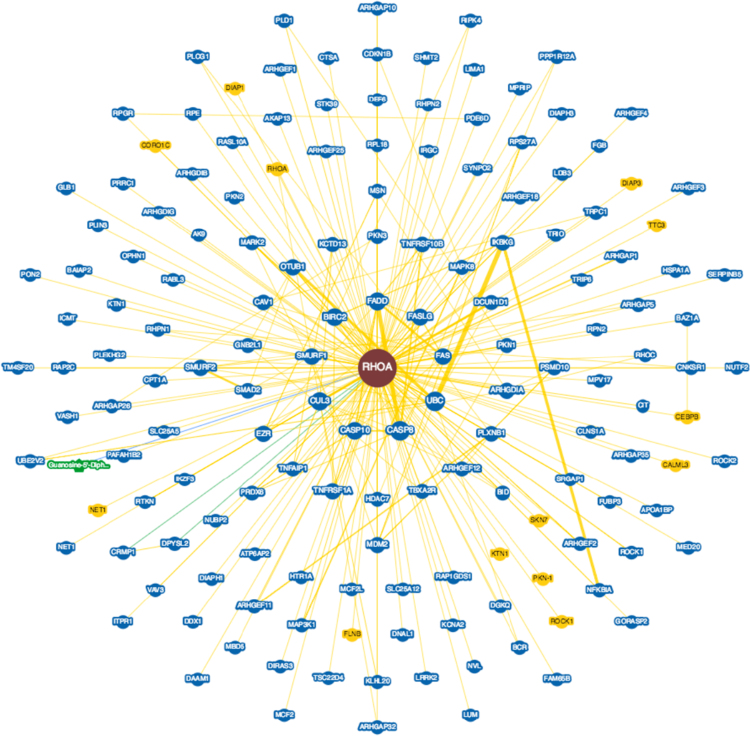
Interactions experimentally determined for the subexpressed Transforming protein RhoA or Ras homology family member A (*RHOA*, UniProtKB ID P61586) in brains with Alzheimer׳s disease in BioGRID database.

**Fig. 12:**
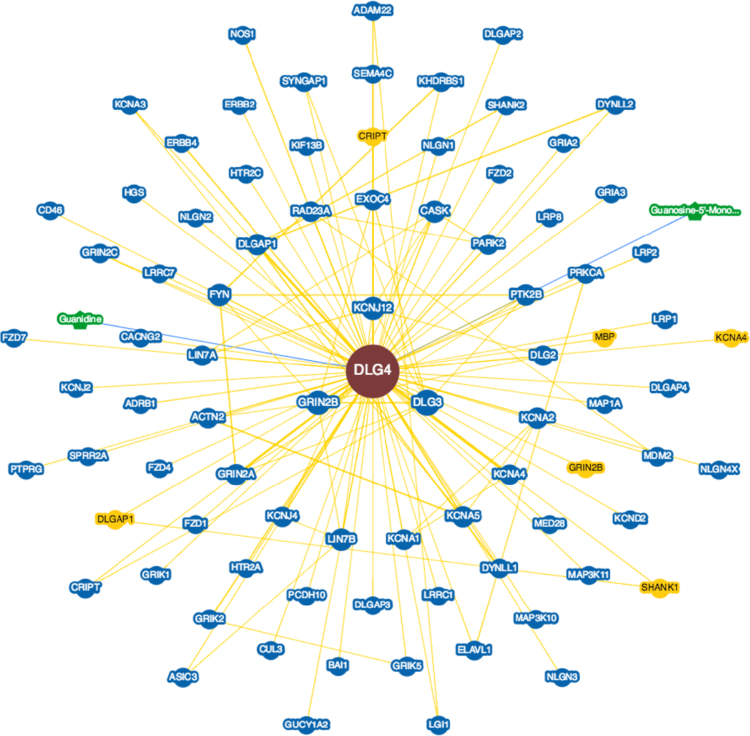
Interactions experimentally determined for the Disks large homolog 4 protein (*DLG4*, UniProtKB ID P78352) in brains with Alzheimer׳s disease in BioGRID database.

**Fig. 13:**
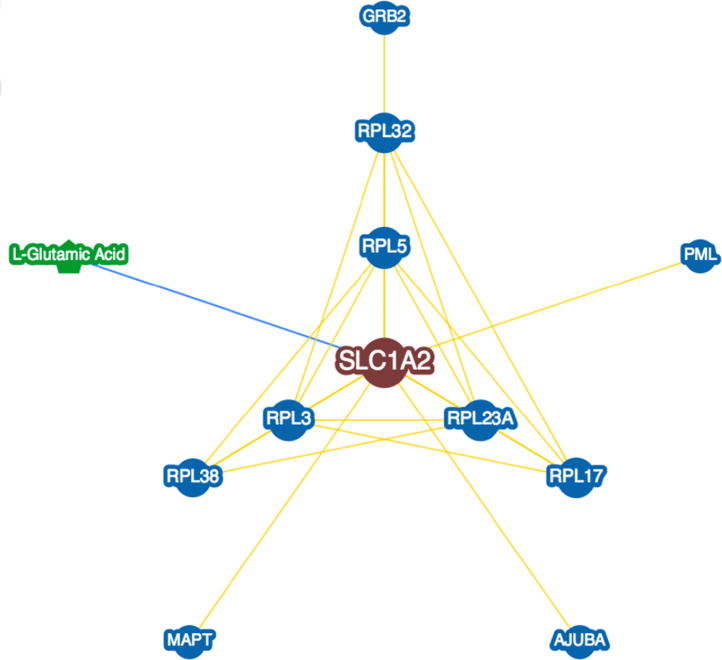
Interactions experimentally determined for the solute carrier family 1 (glial high affinity glutamate transporter), member 2, also known as Excitatory amino acid transporter 2 (*SLC1A2*, UniProtKB ID P43004) in brains with Alzheimer׳s disease in BioGRID database.

The data provides a list of identified 721 iTRAQ-labeled proteins with at least one peptide with ≥95% in confidence (Table 1). Polypeptides that changed in their expression levels were classified according to diseases and biofunctions with the IPA software (Table 2) and analyzed with IPA Core I into different functional networks (Fig. 1, Fig. 2, Fig. 3). We included the mapping of proteins with differential expression into metabolic (Fig. 4) and oxidative phosphorylation (Fig. 5) processes, and determined different interaction networks using IntAct (Fig. 6, Fig. 9) and BioGRID (Fig. 7, Fig. 8, Fig. 10, Fig. 11, Fig. 12, Fig. 13) databases.

## 2 Experimental design, materials and methods

The analyses performed in this data article were focused on the over- or sub-expressed proteins that changed in their expression level in ≥15% or ≤15%, respectively.

### 2.1 IPA Core Analysis of proteins that changed in their expression level in all Alzheimer׳s disease brains

For the classification of identified overexpressed and subexpressed proteins in all Alzheimer׳s disease brains [1] in diseases and biological functions, canonical pathways, molecular networks and the selection of putative candidates for biomarkers, we used the module Core Analysis and Biomarkers filter of the full version of the Ingenuity Pathway Analysis software (IPA, QIAGEN׳s Redwood City, www.qiagen.com/ingenuity). We performed one analysis named Core I. In this analysis we used tissues and primary cells, including nervous system, organ systems, and other tissues and primary cells. The filter also included cell lines of Central Nervous System (CNS), neuroblastoma, leukemia, osteosarcoma, melanoma, lymphoma, immune system, macrophage, lung, breast, hepatoma, pancreatic, kidney, myeloma, ovarian, cervical, prostate, teratocarcinoma and colon cancer, and other cell lines, using a stringent filter. The Fisher׳s Exact Test was employed for the determination of the *p*-value. It was considered as significant with values lower than 0.05. Analysis included the following parameters: (1) Ingenuity Knowledge Base (genes only), considering direct and indirect relationships. (2) Interaction networks including endogenous chemicals, default value of 35 molecules per network and 25 networks per analysis. (3) All data source. (4) Confidence: experimentally observed, highly and moderately predicted. (5) Human species with stringent filter. (6) All mutations.

### 2.2 IPA biomarker analysis

In order to select some putative biomarkers, we filtered all selected proteins through IPA software using only the Biomarkers I analyses. The parameters of Biomarkers filter I included all tissues and primary cells, and all cell lines, all diseases and all biomarkers application, and biomarkers diseases. It also included the following parameters: (1) human species, (2) all molecules type, and (3) all biofluids.

### 2.3 Bioinformatics analyses in KEGG, IntAct and BioGRID databases

The proteins that showed a change in their expression levels in the three AD brains were mapped in the biochemical pathways of the Kyoto Encyclopedia of Genes and Genomes (KEGG) (http://www.genome.jp/kegg/). For the determination of interactions maps, sets of overexpressed and subexpressed proteins were submitted for their analysis to the IntAct Molecular Interaction Database at the European Bioinformatics Institute (http://www.ebi.ac.uk/intact/) [2]. To identify interactors that have been experimentally identified for selected overexpressed and subexpressed proteins, their IDs were submitted to the BioGRID database (Biological General Repository for Interaction Datasets, http://thebiogrid.org/) [3], using the current index version 3.4.133.
