# Supplementary material for: Data set of interactomes and metabolic pathways of proteins differentially expressed in brains with Alzheimer׳s disease
Source: Data Brief. 2016 May 6;7:1707–19. doi: 10.1016/j.dib.2016.04.071 (PMC4878460; doi:10.1016/j.dib.2016.04.071)
Supplement: Supplementary file 1 — Supplementary material [file mmc1.docx]

Authors declare that there is no conflict of interest.
