# Supplementary material for: Data set of interactomes and metabolic pathways of proteins differentially expressed in brains with Alzheimer׳s disease
Source: Data Brief. 2016 May 6;7:1707–19. doi: 10.1016/j.dib.2016.04.071 (PMC4878460; doi:10.1016/j.dib.2016.04.071)
Supplement: Supplementary file 2 — Table 1. [file mmc2.docx]

**Table 1. List of polypeptides identified in the quantitative analysis of iTRAQ-labeled whole protein extracts of brains with Alzheimer’s disease in comparison to a normal brain by tandem mass spectrometry.**

|  | **Polypeptide** | **UniProt/KB ID^a^** | **Unused^b^** | **% Cov. (≥95)^c^** | **Pep. Ident. (≥95)^d^** | **114:113 ^e^** | **116:113 ^f^** | **118:113 ^g^** |
| --- | --- | --- | --- | --- | --- | --- | --- | --- |
|  | Spectrin alpha chain, brain GN=SPTAN1 PE=1 SV=3 | Q13813\|SPTA2 | 120.45 | 34.34 | 90 | 0.9652 | 0.91 | 0.8946 |
|  | Spectrin beta chain, brain 1 GN=SPTBN1 PE=1 SV=2 | Q01082\|SPTB2 | 86.22 | 25.04 | 57 | 1.0283 | 1.00 | 1.0223 |
|  | Glial fibrillary acidic protein GN=GFAP PE=1 SV=1 | P14136\|GFAP | 69.18 | 66.20 | 68 | 1.8510 | 1.49 | 3.4907 |
|  | Clathrin heavy chain 1 GN=CLTC PE=1 SV=5 | Q00610\|CLH1 | 52.81 | 22.39 | 43 | 0.8370 | 1.02 | 0.9802 |
|  | Glyceraldehyde-3-phosphate dehydrogenase GN=GAPDH PE=1 SV=3 | P04406\|G3P | 50.51 | 73.13 | 58 | 1.4980 | 1.21 | 0.7613 |
|  | Heat shock cognate 71 kDa protein GN=HSPA8 PE=1 SV=1 | P11142\|HSP7C | 47.29 | 50.46 | 34 | 1.1613 | 0.94 | 0.8157 |
|  | ATP synthase subunit beta, mitochondrial GN=ATP5B PE=1 SV=3 | P06576\|ATPB | 47.03 | 71.08 | 56 | 0.8748 | 0.83 | 0.5912 |
|  | Tubulin beta-4B chain GN=TUBB4B PE=1 SV=1 | P68371\|TBB4B | 45.68 | 62.25 | 94 | 0.6422 | 0.71 | 0.9867 |
|  | Actin, cytoplasmic 1 GN=ACTB PE=1 SV=1 | P60709\|ACTB | 44.41 | 64.80 | 84 | 1.1083 | 1.02 | 1.3489 |
|  | Plectin GN=PLEC PE=1 SV=3 | Q15149\|PLEC | 43.46 | 6.00 | 23 | 1.0180 | 0.98 | 1.2675 |
|  | Dihydropyrimidinase-related protein 2 GN=DPYSL2 PE=1 SV=1 | Q16555\|DPYL2 | 42.47 | 59.44 | 51 | 1.2647 | 0.96 | 0.8486 |
|  | Syntaxin-binding protein 1 GN=STXBP1 PE=1 SV=1 | P61764\|STXB1 | 40.86 | 40.07 | 24 | 0.9728 | 1.05 | 0.7771 |
|  | Vimentin GN=VIM PE=1 SV=4 | P08670\|VIME | 39.26 | 49.79 | 31 | 0.9343 | 1.27 | 3.4983 |
|  | Alpha-enolase GN=ENO1 PE=1 SV=2 | P06733\|ENOA | 39.02 | 62.67 | 39 | 1.3500 | 1.03 | 1.0833 |
|  | Collagen alpha-1(I) chain GN=COL1A1 PE=1 SV=5 | P02452\|CO1A1 | 38.25 | 35.31 | 53 | 1.0710 | 2.29 | 7.7474 |
|  | ATP synthase subunit alpha, mitochondrial GN=ATP5A1 PE=1 SV=1 | P25705\|ATPA | 35.64 | 44.30 | 32 | 0.9377 | 1.00 | 0.8508 |
|  | Sodium/potassium-transporting ATPase subunit alpha-3 GN=ATP1A3 PE=1 SV=3 | P13637\|AT1A3 | 33.11 | 21.13 | 26 | 0.7447 | 1.05 | 0.8880 |
|  | 2',3'-cyclic-nucleotide 3'-phosphodiesterase GN=CNP PE=1 SV=2 | P09543\|CN37 | 32.99 | 41.81 | 25 | 2.0383 | 1.85 | 0.9845 |
|  | Collagen alpha-2(I) chain GN=COL1A2 PE=1 SV=7 | P08123\|CO1A2 | 32.04 | 36.24 | 50 | 1.4193 | 1.98 | 6.1003 |
|  | Pyruvate kinase isozymes M1/M2 GN=PKM2 PE=1 SV=4 | P14618\|KPYM | 31.44 | 42.75 | 21 | 1.4233 | 1.10 | 1.0363 |
|  | Alpha-internexin GN=INA PE=1 SV=2 | Q16352\|AINX | 30.63 | 40.48 | 24 | 1.3234 | 1.19 | 0.7543 |
|  | Hemoglobin subunit beta GN=HBB PE=1 SV=2 | P68871\|HBB | 29.86 | 95.24 | 58 | 1.0630 | 1.75 | 1.8697 |
|  | Fructose-bisphosphate aldolase A GN=ALDOA PE=1 SV=2 | P04075\|ALDOA | 29.16 | 46.43 | 40 | 1.4460 | 1.05 | 0.9211 |
|  | Creatine kinase B-type GN=CKB PE=1 SV=1 | P12277\|KCRB | 28.48 | 50.39 | 30 | 0.9284 | 1.04 | 0.7266 |
|  | Heat shock protein HSP 90-alpha GN=HSP90AA1 PE=1 SV=5 | P07900\|HS90A | 28.25 | 31.15 | 20 | 1.2586 | 0.97 | 1.0833 |
|  | Gamma-enolase GN=ENO2 PE=1 SV=3 | P09104\|ENOG | 27.98 | 64.06 | 50 | 1.2930 | 1.04 | 0.7953 |
|  | 60 kDa heat shock protein, mitochondrial GN=HSPD1 PE=1 SV=2 | P10809\|CH60 | 27.05 | 35.43 | 23 | 0.9463 | 0.98 | 0.9414 |
|  | Neurofilament light polypeptide GN=NEFL PE=1 SV=3 | P07196\|NFL | 26.41 | 34.99 | 32 | 1.6741 | 1.32 | 0.7756 |
|  | Hexokinase-1 GN=HK1 PE=1 SV=3 | P19367\|HXK1 | 25.87 | 16.14 | 13 | 0.9386 | 0.93 | 0.7623 |
|  | Dynamin-1 GN=DNM1 PE=1 SV=2 | Q05193\|DYN1 | 24.49 | 20.02 | 20 | 0.7909 | 0.82 | 0.7761 |
|  | Alpha-actinin-1 GN=ACTN1 PE=1 SV=2 | P12814\|ACTN1 | 22.48 | 14.80 | 13 | 0.8009 | 0.77 | 1.1386 |
|  | V-type proton ATPase subunit B, brain isoform GN=ATP6V1B2 PE=1 SV=3 | P21281\|VATB2 | 22.18 | 36.20 | 15 | 0.9630 | 0.89 | 0.8345 |
|  | Synapsin-1 GN=SYN1 PE=1 SV=3 | P17600\|SYN1 | 21.99 | 22.98 | 16 | 0.9945 | 0.94 | 0.7438 |
|  | 14-3-3 protein zeta/delta GN=YWHAZ PE=1 SV=1 | P63104\|1433Z | 21.71 | 56.73 | 16 | 1.0413 | 0.99 | 0.9460 |
|  | L-lactate dehydrogenase B chain GN=LDHB PE=1 SV=2 | P07195\|LDHB | 21.59 | 42.51 | 18 | 1.5374 | 1.25 | 1.2121 |
|  | Microtubule-associated protein 1B GN=MAP1B PE=1 SV=2 | P46821\|MAP1B | 21.44 | 6.44 | 12 | 0.9884 | 0.96 | 0.9191 |
|  | Heat shock 70 kDa protein 1A/1B GN=HSPA1A PE=1 SV=5 | P08107\|HSP71 | 21.35 | 34.63 | 20 | 1.3459 | 1.09 | 1.2446 |
|  | Calcium/calmodulin-dependent protein kinase type II subunit alpha GN=CAMK2A PE=1 SV=2 | Q9UQM7\|KCC2A | 21.03 | 29.92 | 12 | 0.7870 | 0.94 | 0.8364 |
|  | Filamin-A GN=FLNA PE=1 SV=4 | P21333\|FLNA | 20.9 | 6.42 | 11 | 0.9305 | 1.03 | 2.6750 |
|  | Fructose-bisphosphate aldolase C GN=ALDOC PE=1 SV=2 | P09972\|ALDOC | 20.69 | 38.46 | 19 | 1.3657 | 1.07 | 0.9671 |
|  | Hemoglobin subunit alpha GN=HBA1 PE=1 SV=2 | P69905\|HBA | 20.13 | 71.83 | 59 | 1.4277 | 2.09 | 2.9908 |
|  | Guanine nucleotide-binding protein G(o) subunit alpha GN=GNAO1 PE=1 SV=4 | P09471\|GNAO | 19.78 | 37.85 | 18 | 0.8464 | 1.02 | 0.9604 |
|  | Neurofilament medium polypeptide GN=NEFM PE=1 SV=3 | P07197\|NFM | 19.69 | 22.05 | 24 | 1.8731 | 1.39 | 0.7663 |
|  | Cytoplasmic dynein 1 heavy chain 1 GN=DYNC1H1 PE=1 SV=5 | Q14204\|DYHC1 | 19.64 | 2.76 | 9 | 0.9690 | 1.00 | 0.9841 |
|  | Triosephosphate isomerase GN=TPI1 PE=1 SV=3 | P60174\|TPIS | 19.21 | 45.10 | 20 | 1.1717 | 1.10 | 0.8760 |
|  | Glutamate dehydrogenase 1, mitochondrial GN=GLUD1 PE=1 SV=2 | P00367\|DHE3 | 18.78 | 29.39 | 13 | 0.9747 | 0.93 | 1.1186 |
|  | Collagen alpha-3(VI) chain GN=COL6A3 PE=1 SV=5 | P12111\|CO6A3 | 18.38 | 4.53 | 12 | 0.9403 | 1.18 | 3.3170 |
|  | Syntaxin-1B GN=STX1B PE=2 SV=1 | P61266\|STX1B | 17.79 | 39.58 | 10 | 0.9034 | 1.12 | 1.0636 |
|  | Neural cell adhesion molecule 1 GN=NCAM1 PE=1 SV=3 | P13591\|NCAM1 | 17.34 | 19.35 | 11 | 0.9092 | 0.94 | 0.9632 |
|  | Malate dehydrogenase, mitochondrial GN=MDH2 PE=1 SV=3 | P40926\|MDHM | 16.59 | 36.98 | 15 | 1.1781 | 1.08 | 0.8247 |
|  | Tubulin beta-3 chain GN=TUBB3 PE=1 SV=2 | Q13509\|TBB3 | 16.4 | 53.11 | 64 | 0.9735 | 0.87 | 0.6977 |
|  | Tenascin-R GN=TNR PE=1 SV=3 | Q92752\|TENR | 16.28 | 12.44 | 12 | 0.9395 | 0.88 | 0.7641 |
|  | Alpha-1-antitrypsin GN=SERPINA1 PE=1 SV=3 | P01009\|A1AT | 15.98 | 29.43 | 11 | 2.8425 | 1.26 | 1.6218 |
|  | Glucose-6-phosphate isomerase GN=GPI PE=1 SV=4 | P06744\|G6PI | 15.71 | 25.27 | 14 | 1.4903 | 1.10 | 0.9205 |
|  | Phosphoglycerate kinase 1 GN=PGK1 PE=1 SV=3 | P00558\|PGK1 | 15.16 | 34.29 | 16 | 1.5723 | 1.29 | 0.9357 |
|  | Alpha-crystallin B chain GN=CRYAB PE=1 SV=2 | P02511\|CRYAB | 15.05 | 58.86 | 8 | 2.3710 | 1.96 | 1.4280 |
|  | Aspartate aminotransferase, cytoplasmic GN=GOT1 PE=1 SV=3 | P17174\|AATC | 15.02 | 23.49 | 12 | 1.3347 | 1.00 | 0.7973 |
|  | Aconitate hydratase, mitochondrial GN=ACO2 PE=1 SV=2 | Q99798\|ACON | 14.63 | 14.10 | 9 | 1.1018 | 1.06 | 1.0374 |
|  | Synaptosomal-associated protein 25 GN=SNAP25 PE=1 SV=1 | P60880\|SNP25 | 14.59 | 49.51 | 12 | 0.7730 | 0.87 | 0.8009 |
|  | Stress-70 protein, mitochondrial GN=HSPA9 PE=1 SV=2 | P38646\|GRP75 | 14.2 | 19.15 | 10 | 0.9570 | 0.92 | 0.7898 |
|  | Tubulin beta-4A chain GN=TUBB4A PE=1 SV=2 | P04350\|TBB4A | 13.29 | 63.29 | 91 | 0.9970 | 1.01 | 0.8629 |
|  | V-type proton ATPase catalytic subunit A GN=ATP6V1A PE=1 SV=2 | P38606\|VATA | 13.21 | 16.05 | 11 | 0.9376 | 0.91 | 0.6473 |
|  | Peptidyl-prolyl cis-trans isomerase A GN=PPIA PE=1 SV=2 | P62937\|PPIA | 13.18 | 39.39 | 10 | 1.0695 | 0.87 | 1.0659 |
|  | Puromycin-sensitive aminopeptidase GN=NPEPPS PE=1 SV=2 | P55786\|PSA | 13.13 | 11.86 | 10 | 1.2562 | 0.96 | 1.0202 |
|  | Ubiquitin carboxyl-terminal hydrolase isozyme L1 GN=UCHL1 PE=1 SV=2 | P09936\|UCHL1 | 13.12 | 47.98 | 16 | 1.2601 | 1.02 | 0.7353 |
|  | Peroxiredoxin-1 GN=PRDX1 PE=1 SV=1 | Q06830\|PRDX1 | 13.05 | 42.71 | 11 | 1.3748 | 1.15 | 1.8461 |
|  | Malate dehydrogenase, cytoplasmic GN=MDH1 PE=1 SV=4 | P40925\|MDHC | 12.99 | 34.43 | 10 | 1.5781 | 1.37 | 0.9929 |
|  | Myelin basic protein GN=MBP PE=1 SV=3 | P02686\|MBP | 12.91 | 24.01 | 13 | 2.3337 | 2.33 | 0.9314 |
|  | Citrate synthase, mitochondrial GN=CS PE=1 SV=2 | O75390\|CISY | 12.84 | 21.46 | 8 | 1.0298 | 1.08 | 1.0405 |
|  | Sodium/potassium-transporting ATPase subunit alpha-1 GN=ATP1A1 PE=1 SV=1 | P05023\|AT1A1 | 12.75 | 20.14 | 26 | 0.7251 | 1.02 | 0.8215 |
|  | Glycogen phosphorylase, brain form GN=PYGB PE=1 SV=5 | P11216\|PYGB | 12.64 | 9.73 | 7 | 1.0149 | 1.09 | 1.2546 |
|  | Myosin-9 GN=MYH9 PE=1 SV=4 | P35579\|MYH9 | 12.57 | 5.51 | 7 | 1.0646 | 0.94 | 1.3042 |
|  | Contactin-1 GN=CNTN1 PE=1 SV=1 | Q12860\|CNTN1 | 12.42 | 5.60 | 5 | 1.0111 | 1.02 | 0.8315 |
|  | ADP/ATP translocase 1 GN=SLC25A4 PE=1 SV=4 | P12235\|ADT1 | 12.35 | 22.15 | 7 | 0.6094 | 0.81 | 0.7883 |
|  | Alpha-synuclein GN=SNCA PE=1 SV=1 | P37840\|SYUA | 12.2 | 44.29 | 6 | 1.0667 | 0.93 | 0.7878 |
|  | Aspartate aminotransferase, mitochondrial GN=GOT2 PE=1 SV=3 | P00505\|AATM | 12.15 | 20.93 | 7 | 1.0550 | 0.96 | 0.7041 |
|  | Cytochrome b-c1 complex subunit 1, mitochondrial GN=UQCRC1 PE=1 SV=3 | P31930\|QCR1 | 11.96 | 20.83 | 14 | 0.8957 | 0.93 | 0.8299 |
|  | Visinin-like protein 1 GN=VSNL1 PE=1 SV=2 | P62760\|VISL1 | 11.77 | 41.88 | 7 | 1.0129 | 1.07 | 0.9074 |
|  | ATP synthase subunit O, mitochondrial GN=ATP5O PE=1 SV=1 | P48047\|ATPO | 11.55 | 37.56 | 7 | 1.0607 | 1.11 | 1.0419 |
|  | Vesicle-fusing ATPase GN=NSF PE=1 SV=3 | P46459\|NSF | 11.51 | 9.41 | 7 | 1.0162 | 1.09 | 1.0757 |
|  | Superoxide dismutase [Mn], mitochondrial GN=SOD2 PE=1 SV=2 | P04179\|SODM | 11.34 | 43.69 | 10 | 1.2173 | 1.17 | 1.6182 |
|  | 14-3-3 protein gamma GN=YWHAG PE=1 SV=2 | P61981\|1433G | 11.19 | 38.46 | 16 | 0.9619 | 0.96 | 0.6972 |
|  | L-lactate dehydrogenase A chain GN=LDHA PE=1 SV=2 | P00338\|LDHA | 11.17 | 23.80 | 8 | 1.5234 | 1.16 | 0.9969 |
|  | Protein kinase C and casein kinase substrate in neurons protein 1 GN=PACSIN1 PE=1 SV=1 | Q9BY11\|PACN1 | 10.99 | 17.34 | 6 | 1.0352 | 0.93 | 0.7668 |
|  | Histone H2A type 2-A GN=HIST2H2AA3 PE=1 SV=3 | Q6FI13\|H2A2A | 10.99 | 49.23 | 9 | 0.8117 | 0.70 | 0.7060 |
|  | Dihydropyrimidinase-related protein 1 GN=CRMP1 PE=1 SV=1 | Q14194\|DPYL1 | 10.88 | 19.58 | 13 | 0.8302 | 0.76 | 0.6338 |
|  | Septin-7 GN=SEPT7 PE=1 SV=2 | Q16181\|SEPT7 | 10.87 | 20.37 | 8 | 1.2559 | 1.01 | 0.8560 |
|  | Carbonyl reductase [NADPH] 1 GN=CBR1 PE=1 SV=3 | P16152\|CBR1 | 10.81 | 31.41 | 8 | 1.4824 | 1.11 | 1.1431 |
|  | Ig gamma-1 chain C region GN=IGHG1 PE=1 SV=1 | P01857\|IGHG1 | 10.66 | 24.24 | 9 | 3.1305 | 1.57 | 2.2553 |
|  | Rab GDP dissociation inhibitor beta GN=GDI2 PE=1 SV=2 | P50395\|GDIB | 10.66 | 20.22 | 11 | 1.3853 | 1.11 | 0.8431 |
|  | Histone H4 GN=HIST1H4A PE=1 SV=2 | P62805\|H4 | 10.49 | 51.46 | 6 | 1.0375 | 1.46 | 1.3015 |
|  | 14-3-3 protein epsilon GN=YWHAE PE=1 SV=1 | P62258\|1433E | 10.17 | 36.47 | 14 | 1.0590 | 0.98 | 0.9364 |
|  | Protein DJ-1 GN=PARK7 PE=1 SV=2 | Q99497\|PARK7 | 10.13 | 29.63 | 6 | 1.3200 | 1.10 | 1.0745 |
|  | Voltage-dependent anion-selective channel protein 1 GN=VDAC1 PE=1 SV=2 | P21796\|VDAC1 | 10.07 | 31.80 | 7 | 0.8464 | 1.00 | 1.0372 |
|  | Endophilin-A1 GN=SH3GL2 PE=1 SV=1 | Q99962\|SH3G2 | 10.02 | 25.57 | 7 | 0.9703 | 0.88 | 0.6699 |
|  | Tubulin beta-2A chain GN=TUBB2A PE=1 SV=1 | Q13885\|TBB2A | 10 | 62.25 | 92 | 0.8118 | 0.76 | 0.5635 |
|  | Basement membrane-specific heparan sulfate proteoglycan core protein GN=HSPG2 PE=1 SV=4 | P98160\|PGBM | 9.96 | 1.53 | 5 | 1.0823 | 1.12 | 2.4933 |
|  | Tubulin polymerization-promoting protein GN=TPPP PE=1 SV=1 | O94811\|TPPP | 9.96 | 35.62 | 6 | 1.1999 | 1.08 | 0.7788 |
|  | Phosphoglycerate mutase 1 GN=PGAM1 PE=1 SV=2 | P18669\|PGAM1 | 9.94 | 25.20 | 10 | 1.1427 | 1.00 | 0.8105 |
|  | Band 4.1-like protein 3 GN=EPB41L3 PE=1 SV=2 | Q9Y2J2\|E41L3 | 9.92 | 4.88 | 5 | 1.0134 | 1.14 | 0.9160 |
|  | ADP-ribosylation factor 1 GN=ARF1 PE=1 SV=2 | P84077\|ARF1 | 9.89 | 39.23 | 8 | 1.0262 | 0.94 | 0.6912 |
|  | 6-phosphofructokinase, liver type GN=PFKL PE=1 SV=6 | P17858\|K6PL | 9.83 | 8.72 | 6 | 0.9713 | 1.01 | 1.3703 |
|  | Methylmalonate-semialdehyde dehydrogenase [acylating], mitochondrial GN=ALDH6A1 PE=1 SV=2 | Q02252\|MMSA | 9.79 | 9.91 | 5 | 1.2929 | 1.32 | 1.3356 |
|  | Annexin A6 GN=ANXA6 PE=1 SV=3 | P08133\|ANXA6 | 9.77 | 9.81 | 5 | 0.7659 | 0.79 | 0.7601 |
|  | Synaptotagmin-1 GN=SYT1 PE=1 SV=1 | P21579\|SYT1 | 9.76 | 14.69 | 6 | 0.7667 | 0.90 | 0.5881 |
|  | Fascin GN=FSCN1 PE=1 SV=3 | Q16658\|FSCN1 | 9.71 | 14.60 | 6 | 1.0288 | 0.79 | 0.7935 |
|  | Cofilin-1 GN=CFL1 PE=1 SV=3 | P23528\|COF1 | 9.67 | 49.40 | 6 | 1.1576 | 1.04 | 0.9178 |
|  | Collagen alpha-1(VI) chain GN=COL6A1 PE=1 SV=3 | P12109\|CO6A1 | 9.64 | 7.49 | 6 | 0.8810 | 1.08 | 2.6092 |
|  | Transitional endoplasmic reticulum ATPase GN=VCP PE=1 SV=4 | P55072\|TERA | 9.55 | 9.31 | 6 | 0.9891 | 0.93 | 0.7999 |
|  | AP-2 complex subunit beta GN=AP2B1 PE=1 SV=1 | P63010\|AP2B1 | 9.33 | 8.75 | 5 | 1.0045 | 1.04 | 0.8595 |
|  | Cathepsin D GN=CTSD PE=1 SV=1 | P07339\|CATD | 9.33 | 16.75 | 6 | 1.2611 | 1.15 | 1.3223 |
|  | Profilin-1 GN=PFN1 PE=1 SV=2 | P07737\|PROF1 | 9.28 | 42.86 | 5 | 1.1212 | 0.98 | 1.1519 |
|  | Succinyl-CoA:3-ketoacid-coenzyme A transferase 1, mitochondrial GN=OXCT1 PE=1 SV=1 | P55809\|SCOT1 | 9.1 | 11.35 | 6 | 0.7931 | 0.88 | 0.8474 |
|  | Peroxiredoxin-2 GN=PRDX2 PE=1 SV=5 | P32119\|PRDX2 | 9.04 | 32.83 | 9 | 1.3470 | 1.15 | 1.1562 |
|  | Heat shock 70 kDa protein 12A GN=HSPA12A PE=1 SV=2 | O43301\|HS12A | 9 | 8.30 | 4 | 0.9361 | 1.04 | 0.8077 |
|  | Elongation factor 1-alpha 2 GN=EEF1A2 PE=1 SV=1 | Q05639\|EF1A2 | 9 | 15.98 | 5 | 1.1208 | 1.03 | 1.0426 |
|  | AP-2 complex subunit alpha-1 GN=AP2A1 PE=1 SV=3 | O95782\|AP2A1 | 8.95 | 8.39 | 6 | 0.9177 | 0.93 | 0.8946 |
|  | Tubulin alpha-4A chain GN=TUBA4A PE=1 SV=1 | P68366\|TBA4A | 8.86 | 47.54 | 67 | 1.0279 | 1.16 | 0.9198 |
|  | Ubiquitin-like modifier-activating enzyme 1 GN=UBA1 PE=1 SV=3 | P22314\|UBA1 | 8.85 | 8.32 | 9 | 1.0289 | 0.92 | 1.0533 |
|  | Dihydropteridine reductase GN=QDPR PE=1 SV=2 | P09417\|DHPR | 8.76 | 38.93 | 7 | 1.8002 | 1.30 | 0.9898 |
|  | N(G),N(G)-dimethylarginine dimethylaminohydrolase 1 GN=DDAH1 PE=1 SV=3 | O94760\|DDAH1 | 8.74 | 25.61 | 6 | 1.1920 | 1.04 | 1.0682 |
|  | Haptoglobin GN=HP PE=1 SV=1 | P00738\|HPT | 8.65 | 12.32 | 5 | 3.5817 | 1.42 | 2.2968 |
|  | Sodium/potassium-transporting ATPase subunit alpha-2 GN=ATP1A2 PE=1 SV=1 | P50993\|AT1A2 | 8.59 | 16.47 | 22 | 0.6754 | 1.08 | 1.4427 |
|  | Ferritin light chain GN=FTL PE=1 SV=2 | P02792\|FRIL | 8.58 | 25.71 | 7 | 1.5072 | 1.38 | 2.6519 |
|  | Transketolase GN=TKT PE=1 SV=3 | P29401\|TKT | 8.49 | 19.74 | 8 | 1.2621 | 1.07 | 1.0525 |
|  | Prohibitin GN=PHB PE=1 SV=1 | P35232\|PHB | 8.48 | 27.21 | 6 | 0.7881 | 0.85 | 0.6128 |
|  | Versican core protein GN=VCAN PE=1 SV=3 | P13611\|CSPG2 | 8.4 | 2.59 | 7 | 2.3505 | 1.60 | 2.0642 |
|  | Annexin A5 GN=ANXA5 PE=1 SV=2 | P08758\|ANXA5 | 8.3 | 15.94 | 4 | 1.2420 | 1.17 | 2.0336 |
|  | Immunoglobulin superfamily member 8 GN=IGSF8 PE=1 SV=1 | Q969P0\|IGSF8 | 8.24 | 13.21 | 7 | 0.9139 | 1.01 | 0.8298 |
|  | Tubulin beta chain GN=TUBB PE=1 SV=2 | P07437\|TBB5 | 8.2 | 62.39 | 87 | 0.8657 | 0.82 | 0.6528 |
|  | Creatine kinase U-type, mitochondrial GN=CKMT1A PE=1 SV=1 | P12532\|KCRU | 8.2 | 18.23 | 5 | 1.0205 | 1.00 | 0.7705 |
|  | Carbonic anhydrase 2 GN=CA2 PE=1 SV=2 | P00918\|CAH2 | 8.19 | 25.00 | 5 | 2.2137 | 1.46 | 1.2316 |
|  | Peroxiredoxin-5, mitochondrial GN=PRDX5 PE=1 SV=4 | P30044\|PRDX5 | 8.12 | 24.77 | 7 | 1.0716 | 0.86 | 0.8957 |
|  | Microtubule-associated protein 2 GN=MAP2 PE=1 SV=4 | P11137\|MAP2 | 8.11 | 3.18 | 4 | 0.7792 | 0.86 | 0.6807 |
|  | Serine/threonine-protein phosphatase 2A 65 kDa regulatory subunit A alpha isoform GN=PPP2R1A PE=1 SV=4 | P30153\|2AAA | 8.03 | 7.13 | 4 | 1.0261 | 0.98 | 0.7779 |
|  | Ankyrin-2 GN=ANK2 PE=1 SV=3 | Q01484\|ANK2 | 7.98 | 2.09 | 6 | 0.8813 | 0.92 | 0.8367 |
|  | Prohibitin-2 GN=PHB2 PE=1 SV=2 | Q99623\|PHB2 | 7.96 | 18.73 | 6 | 0.7033 | 0.87 | 0.6932 |
|  | Hypoxanthine-guanine phosphoribosyltransferase GN=HPRT1 PE=1 SV=2 | P00492\|HPRT | 7.95 | 21.56 | 4 | 1.0673 | 0.88 | 0.7851 |
|  | Ras-related protein Rab-3A GN=RAB3A PE=1 SV=1 | P20336\|RAB3A | 7.94 | 25.00 | 5 | 0.8832 | 0.83 | 0.9323 |
|  | Amphiphysin GN=AMPH PE=1 SV=1 | P49418\|AMPH | 7.85 | 13.38 | 7 | 1.2013 | 1.02 | 0.8984 |
|  | Trifunctional enzyme subunit alpha, mitochondrial GN=HADHA PE=1 SV=2 | P40939\|ECHA | 7.77 | 10.09 | 5 | 1.1561 | 1.12 | 1.3814 |
|  | Syntaxin-1A GN=STX1A PE=1 SV=1 | Q16623\|STX1A | 7.73 | 32.64 | 7 | 0.8569 | 0.87 | 0.7714 |
|  | Spectrin beta chain, brain 2 GN=SPTBN2 PE=1 SV=3 | O15020\|SPTN2 | 7.69 | 3.51 | 6 | 0.8863 | 1.05 | 1.3295 |
|  | Peroxiredoxin-6 GN=PRDX6 PE=1 SV=3 | P30041\|PRDX6 | 7.61 | 29.46 | 10 | 1.4512 | 1.20 | 1.2090 |
|  | Dynamin-1-like protein GN=DNM1L PE=1 SV=2 | O00429\|DNM1L | 7.59 | 7.47 | 4 | 0.9993 | 0.96 | 0.9604 |
|  | Ferritin heavy chain GN=FTH1 PE=1 SV=2 | P02794\|FRIH | 7.55 | 22.40 | 7 | 1.8815 | 1.47 | 1.7327 |
|  | Dihydropyrimidinase-related protein 3 GN=DPYSL3 PE=1 SV=1 | Q14195\|DPYL3 | 7.48 | 18.07 | 14 | 1.1657 | 0.78 | 1.0197 |
|  | Myelin-associated glycoprotein GN=MAG PE=1 SV=1 | P20916\|MAG | 7.48 | 8.15 | 7 | 1.8070 | 1.28 | 0.8919 |
|  | Mitochondrial 2-oxoglutarate/malate carrier protein GN=SLC25A11 PE=1 SV=3 | Q02978\|M2OM | 7.4 | 21.34 | 5 | 0.8284 | 0.91 | 0.8845 |
|  | Calcium-binding mitochondrial carrier protein Aralar1 GN=SLC25A12 PE=1 SV=2 | O75746\|CMC1 | 7.37 | 9.29 | 4 | 0.7148 | 0.82 | 0.9648 |
|  | Biglycan GN=BGN PE=1 SV=2 | P21810\|PGS1 | 7.31 | 17.12 | 9 | 0.9688 | 1.43 | 2.9870 |
|  | Serine/threonine-protein phosphatase 2B catalytic subunit alpha isoform GN=PPP3CA PE=1 SV=1 | Q08209\|PP2BA | 7.22 | 10.56 | 5 | 0.9447 | 0.91 | 0.9152 |
|  | Protein NDRG2 GN=NDRG2 PE=1 SV=2 | Q9UN36\|NDRG2 | 7.19 | 16.17 | 4 | 0.9964 | 1.10 | 1.2348 |
|  | 4-aminobutyrate aminotransferase, mitochondrial GN=ABAT PE=1 SV=3 | P80404\|GABT | 7.14 | 8.60 | 6 | 0.9553 | 1.06 | 0.8387 |
|  | Gelsolin GN=GSN PE=1 SV=1 | P06396\|GELS | 7.04 | 7.67 | 6 | 1.5422 | 1.40 | 1.8915 |
|  | Isocitrate dehydrogenase [NADP], mitochondrial GN=IDH2 PE=1 SV=2 | P48735\|IDHP | 7.01 | 9.74 | 4 | 0.9142 | 1.01 | 1.0554 |
|  | Adenylate kinase isoenzyme 1 GN=AK1 PE=1 SV=3 | P00568\|KAD1 | 6.97 | 23.71 | 4 | 1.2401 | 1.21 | 1.1486 |
|  | T-complex protein 1 subunit beta GN=CCT2 PE=1 SV=4 | P78371\|TCPB | 6.93 | 11.21 | 4 | 0.7383 | 0.71 | 0.5601 |
|  | Phosphatidylethanolamine-binding protein 1 GN=PEBP1 PE=1 SV=3 | P30086\|PEBP1 | 6.91 | 40.11 | 7 | 1.2035 | 0.98 | 0.8965 |
|  | Guanine nucleotide-binding protein G(I)/G(S)/G(T) subunit beta-2 GN=GNB2 PE=1 SV=3 | P62879\|GBB2 | 6.87 | 13.24 | 4 | 0.8730 | 0.94 | 1.0641 |
|  | Isocitrate dehydrogenase [NAD] subunit alpha, mitochondrial GN=IDH3A PE=1 SV=1 | P50213\|IDH3A | 6.77 | 14.21 | 5 | 1.0769 | 1.03 | 0.7474 |
|  | Cytochrome b-c1 complex subunit 2, mitochondrial GN=UQCRC2 PE=1 SV=3 | P22695\|QCR2 | 6.75 | 21.19 | 7 | 0.9149 | 1.00 | 0.9131 |
|  | Vinculin GN=VCL PE=1 SV=4 | P18206\|VINC | 6.64 | 6.70 | 4 | 1.0495 | 0.94 | 1.8866 |
|  | Heat shock protein beta-1 GN=HSPB1 PE=1 SV=2 | P04792\|HSPB1 | 6.62 | 20.00 | 3 | 1.1752 | 1.33 | 3.0783 |
|  | Prelamin-A/C GN=LMNA PE=1 SV=1 | P02545\|LMNA | 6.46 | 6.02 | 3 | 0.9441 | 0.92 | 1.3788 |
|  | Dihydrolipoyl dehydrogenase, mitochondrial GN=DLD PE=1 SV=2 | P09622\|DLDH | 6.46 | 16.11 | 4 | 1.0009 | 1.12 | 1.0027 |
|  | Synapsin-2 GN=SYN2 PE=1 SV=3 | Q92777\|SYN2 | 6.41 | 10.82 | 6 | 0.7361 | 0.61 | 0.6045 |
|  | T-complex protein 1 subunit zeta GN=CCT6A PE=1 SV=3 | P40227\|TCPZ | 6.27 | 9.61 | 3 | 0.9318 | 0.99 | 0.8395 |
|  | Histone H2B type 1-O GN=HIST1H2BO PE=1 SV=3 | P23527\|H2B1O | 6.26 | 27.78 | 7 | 1.2509 | 1.30 | 1.3375 |
|  | Transgelin GN=TAGLN PE=1 SV=4 | Q01995\|TAGL | 6.22 | 28.36 | 7 | 0.9900 | 1.05 | 3.2208 |
|  | Elongation factor Tu, mitochondrial GN=TUFM PE=1 SV=2 | P49411\|EFTU | 6.1 | 14.16 | 5 | 1.1489 | 1.14 | 0.9895 |
|  | Gamma-soluble NSF attachment protein GN=NAPG PE=1 SV=1 | Q99747\|SNAG | 6 | 10.90 | 3 | 0.9380 | 1.11 | 0.9988 |
|  | Mitogen-activated protein kinase 1 GN=MAPK1 PE=1 SV=3 | P28482\|MK01 | 5.98 | 15.56 | 5 | 1.0157 | 1.01 | 1.4214 |
|  | V-type proton ATPase subunit E 1 GN=ATP6V1E1 PE=1 SV=1 | P36543\|VATE1 | 5.91 | 20.80 | 4 | 0.8732 | 0.89 | 0.6625 |
|  | Neutral alpha-glucosidase AB GN=GANAB PE=1 SV=3 | Q14697\|GANAB | 5.9 | 6.67 | 4 | 0.9997 | 1.16 | 1.1535 |
|  | Myosin-Va GN=MYO5A PE=1 SV=2 | Q9Y4I1\|MYO5A | 5.85 | 1.94 | 4 | 0.8376 | 1.02 | 0.9339 |
|  | Neuronal cell adhesion molecule GN=NRCAM PE=1 SV=3 | Q92823\|NRCAM | 5.73 | 3.99 | 4 | 0.8372 | 0.85 | 0.7013 |
|  | Heterogeneous nuclear ribonucleoproteins A2/B1 GN=HNRNPA2B1 PE=1 SV=2 | P22626\|ROA2 | 5.67 | 17.00 | 3 | 0.8741 | 0.94 | 0.8978 |
|  | Cullin-associated NEDD8-dissociated protein 1 GN=CAND1 PE=1 SV=2 | Q86VP6\|CAND1 | 5.65 | 4.15 | 4 | 1.0569 | 1.02 | 1.1040 |
|  | Glutathione S-transferase P GN=GSTP1 PE=1 SV=2 | P09211\|GSTP1 | 5.6 | 29.52 | 4 | 2.1241 | 1.50 | 1.8281 |
|  | Neurofascin GN=NFASC PE=1 SV=4 | O94856\|NFASC | 5.58 | 2.75 | 3 | 1.1136 | 1.14 | 0.9212 |
|  | Calcium-dependent secretion activator 1 GN=CADPS PE=1 SV=3 | Q9ULU8\|CAPS1 | 5.51 | 2.59 | 3 | 1.0040 | 1.05 | 0.8248 |
|  | Nucleoside diphosphate kinase A GN=NME1 PE=1 SV=1 | P15531\|NDKA | 5.51 | 26.32 | 3 | 1.2809 | 1.24 | 1.0449 |
|  | NADH dehydrogenase [ubiquinone] iron-sulfur protein 3, mitochondrial GN=NDUFS3 PE=1 SV=1 | O75489\|NDUS3 | 5.43 | 16.67 | 5 | 0.7167 | 0.82 | 0.7645 |
|  | Pyruvate carboxylase, mitochondrial GN=PC PE=1 SV=2 | P11498\|PYC | 5.39 | 4.08 | 3 | 0.8718 | 1.03 | 1.4513 |
|  | Vesicle-associated membrane protein 2 GN=VAMP2 PE=1 SV=3 | P63027\|VAMP2 | 5.33 | 39.66 | 7 | 0.7893 | 0.99 | 0.8882 |
|  | Protein-L-isoaspartate(D-aspartate) O-methyltransferase GN=PCMT1 PE=1 SV=4 | P22061\|PIMT | 5.26 | 12.33 | 3 | 1.1271 | 1.08 | 0.7728 |
|  | Tropomyosin alpha-3 chain GN=TPM3 PE=1 SV=1 | P06753\|TPM3 | 5.22 | 12.32 | 3 | 0.9740 | 0.84 | 1.1884 |
|  | Microtubule-associated protein tau GN=MAPT PE=1 SV=5 | P10636\|TAU | 5.2 | 5.01 | 3 | 0.9807 | 1.20 | 1.7345 |
|  | Ras-related protein Rab-1B GN=RAB1B PE=1 SV=1 | Q9H0U4\|RAB1B | 5 | 17.41 | 3 | 1.0418 | 1.03 | 1.1242 |
|  | Polyubiquitin-C GN=UBC PE=1 SV=2 | P0CG48\|UBC | 4.99 | 32.85 | 3 | 1.4257 | 1.10 | 1.2864 |
|  | Cytochrome c GN=CYCS PE=1 SV=2 | P99999\|CYC | 4.96 | 24.76 | 4 | 1.1072 | 1.09 | 0.8297 |
|  | Actin-related protein 2 GN=ACTR2 PE=1 SV=1 | P61160\|ARP2 | 4.94 | 15.48 | 3 | 0.9253 | 0.99 | 1.3532 |
|  | Clathrin coat assembly protein AP180 GN=SNAP91 PE=1 SV=2 | O60641\|AP180 | 4.94 | 3.86 | 4 | 1.1644 | 0.90 | 0.8377 |
|  | Myc box-dependent-interacting protein 1 GN=BIN1 PE=1 SV=1 | O00499\|BIN1 | 4.93 | 8.43 | 5 | 1.3172 | 1.08 | 0.8271 |
|  | Protein kinase C beta type GN=PRKCB PE=1 SV=4 | P05771\|KPCB | 4.92 | 7.00 | 3 | 0.7911 | 0.99 | 1.0056 |
|  | Histone H3.3 GN=H3F3A PE=1 SV=2 | P84243\|H33 | 4.88 | 11.76 | 2 | 1.3053 | 1.44 | 1.9806 |
|  | Flavin reductase (NADPH) GN=BLVRB PE=1 SV=3 | P30043\|BLVRB | 4.88 | 16.50 | 3 | 0.8980 | 0.91 | 0.9336 |
|  | Beta-synuclein GN=SNCB PE=1 SV=1 | Q16143\|SYUB | 4.84 | 41.79 | 4 | 1.0845 | 0.95 | 0.8638 |
|  | Ras-related protein Rab-2B GN=RAB2B PE=1 SV=1 | Q8WUD1\|RAB2B | 4.82 | 12.96 | 2 | 0.8633 | 0.94 | 0.9224 |
|  | Pyruvate dehydrogenase E1 component subunit beta, mitochondrial GN=PDHB PE=1 SV=3 | P11177\|ODPB | 4.79 | 12.26 | 3 | 0.8356 | 0.98 | 0.8712 |
|  | Neurofilament heavy polypeptide GN=NEFH PE=1 SV=4 | P12036\|NFH | 4.78 | 5.26 | 6 | 1.4705 | 1.21 | 0.8625 |
|  | Plasma membrane calcium-transporting ATPase 4 GN=ATP2B4 PE=1 SV=2 | P23634\|AT2B4 | 4.76 | 2.02 | 2 | 0.7336 | 0.80 | 0.9386 |
|  | Cytochrome c oxidase subunit 5A, mitochondrial GN=COX5A PE=1 SV=2 | P20674\|COX5A | 4.7 | 22.00 | 3 | 0.7200 | 0.93 | 0.5653 |
|  | Elongation factor 1-gamma GN=EEF1G PE=1 SV=3 | P26641\|EF1G | 4.66 | 7.55 | 3 | 1.1205 | 1.00 | 1.2632 |
|  | Heat shock protein HSP 90-beta GN=HSP90AB1 PE=1 SV=4 | P08238\|HS90B | 4.65 | 21.82 | 16 | 0.7979 | 0.76 | 0.9293 |
|  | Aldehyde dehydrogenase, mitochondrial GN=ALDH2 PE=1 SV=2 | P05091\|ALDH2 | 4.63 | 10.06 | 4 | 1.0507 | 1.03 | 1.1571 |
|  | Guanine nucleotide-binding protein G(i) subunit alpha-1 GN=GNAI1 PE=1 SV=2 | P63096\|GNAI1 | 4.62 | 15.25 | 8 | 0.6573 | 0.90 | 0.6777 |
|  | T-complex protein 1 subunit alpha GN=TCP1 PE=1 SV=1 | P17987\|TCPA | 4.61 | 3.96 | 2 | 1.0074 | 1.03 | 0.8275 |
|  | Galectin-3 GN=LGALS3 PE=1 SV=5 | P17931\|LEG3 | 4.61 | 11.60 | 3 | 1.0381 | 1.12 | 1.3675 |
|  | Talin-1 GN=TLN1 PE=1 SV=3 | Q9Y490\|TLN1 | 4.56 | 2.28 | 5 | 1.0732 | 1.16 | 1.9564 |
|  | Adenosylhomocysteinase GN=AHCY PE=1 SV=4 | P23526\|SAHH | 4.55 | 7.41 | 3 | 1.3916 | 1.03 | 1.2862 |
|  | Ras-related protein Rab-7a GN=RAB7A PE=1 SV=1 | P51149\|RAB7A | 4.54 | 10.14 | 2 | 0.9867 | 0.94 | 1.1243 |
|  | Septin-6 GN=SEPT6 PE=1 SV=4 | Q14141\|SEPT6 | 4.51 | 8.99 | 3 | 1.0899 | 1.03 | 0.8889 |
|  | Fumarate hydratase, mitochondrial GN=FH PE=1 SV=3 | P07954\|FUMH | 4.49 | 13.53 | 4 | 1.0023 | 0.92 | 0.8531 |
|  | Enoyl-CoA hydratase, mitochondrial GN=ECHS1 PE=1 SV=4 | P30084\|ECHM | 4.49 | 12.76 | 3 | 1.0642 | 1.05 | 1.0042 |
|  | Ras-related protein Rab-5B GN=RAB5B PE=1 SV=1 | P61020\|RAB5B | 4.49 | 16.28 | 3 | 0.8451 | 0.81 | 1.0689 |
|  | Collagen alpha-1(XIV) chain GN=COL14A1 PE=1 SV=3 | Q05707\|COEA1 | 4.46 | 2.17 | 3 | 0.8573 | 1.06 | 2.3912 |
|  | Dynactin subunit 2 GN=DCTN2 PE=1 SV=4 | Q13561\|DCTN2 | 4.45 | 8.48 | 3 | 1.1277 | 1.00 | 0.7703 |
|  | NAD(P) transhydrogenase, mitochondrial GN=NNT PE=1 SV=3 | Q13423\|NNTM | 4.44 | 3.22 | 2 | 0.9122 | 1.01 | 0.9503 |
|  | Periostin GN=POSTN PE=1 SV=2 | Q15063\|POSTN | 4.43 | 7.54 | 4 | 1.0986 | 1.00 | 2.2222 |
|  | Heterogeneous nuclear ribonucleoprotein K GN=HNRNPK PE=1 SV=1 | P61978\|HNRPK | 4.43 | 9.29 | 3 | 1.2479 | 1.35 | 1.1039 |
|  | Pyridoxal kinase GN=PDXK PE=1 SV=1 | O00764\|PDXK | 4.42 | 11.86 | 4 | 0.9199 | 0.92 | 0.7777 |
|  | T-complex protein 1 subunit epsilon GN=CCT5 PE=1 SV=1 | P48643\|TCPE | 4.4 | 8.50 | 2 | 1.0414 | 1.13 | 0.6432 |
|  | Neurochondrin GN=NCDN PE=1 SV=1 | Q9UBB6\|NCDN | 4.35 | 3.29 | 2 | 1.3078 | 1.18 | 0.9938 |
|  | 14-3-3 protein beta/alpha GN=YWHAB PE=1 SV=3 | P31946\|1433B | 4.33 | 38.62 | 13 | 0.9362 | 1.02 | 0.6861 |
|  | Fibronectin GN=FN1 PE=1 SV=4 | P02751\|FINC | 4.32 | 1.55 | 3 | 1.1401 | 1.04 | 4.3495 |
|  | Endoplasmin GN=HSP90B1 PE=1 SV=1 | P14625\|ENPL | 4.32 | 2.99 | 2 | 0.8224 | 0.72 | 1.1487 |
|  | NADH dehydrogenase [ubiquinone] flavoprotein 1, mitochondrial GN=NDUFV1 PE=1 SV=4 | P49821\|NDUV1 | 4.32 | 9.70 | 3 | 0.8055 | 0.98 | 0.7153 |
|  | Ectonucleotide pyrophosphatase/phosphodiesterase family member 6 GN=ENPP6 PE=2 SV=2 | Q6UWR7\|ENPP6 | 4.31 | 9.09 | 3 | 1.2886 | 1.12 | 1.1786 |
|  | Fatty acid synthase GN=FASN PE=1 SV=3 | P49327\|FAS | 4.24 | 1.35 | 2 | 1.1071 | 0.83 | 0.8608 |
|  | 6-phosphofructokinase, muscle type GN=PFKM PE=1 SV=2 | P08237\|K6PF | 4.22 | 8.08 | 5 | 1.1311 | 1.13 | 1.1788 |
|  | Myosin-10 GN=MYH10 PE=1 SV=3 | P35580\|MYH10 | 4.22 | 2.73 | 3 | 0.8325 | 0.76 | 0.8685 |
|  | Glutathione S-transferase Mu 2 GN=GSTM2 PE=1 SV=2 | P28161\|GSTM2 | 4.2 | 18.35 | 3 | 0.9729 | 0.96 | 1.1441 |
|  | Ras-related protein Rab-11A GN=RAB11A PE=1 SV=3 | P62491\|RB11A | 4.18 | 18.06 | 3 | 0.7522 | 0.70 | 0.7379 |
|  | Obg-like ATPase 1 GN=OLA1 PE=1 SV=2 | Q9NTK5\|OLA1 | 4.16 | 5.56 | 2 | 1.4681 | 1.27 | 0.8411 |
|  | Heterogeneous nuclear ribonucleoprotein U GN=HNRNPU PE=1 SV=6 | Q00839\|HNRPU | 4.14 | 2.91 | 2 | 0.8971 | 1.02 | 1.0551 |
|  | Sodium/potassium-transporting ATPase subunit beta-1 GN=ATP1B1 PE=1 SV=1 | P05026\|AT1B1 | 4.11 | 8.25 | 2 | 0.8201 | 1.08 | 0.9034 |
|  | Carbonic anhydrase 1 GN=CA1 PE=1 SV=2 | P00915\|CAH1 | 4.11 | 15.33 | 3 | 0.9675 | 1.63 | 1.5742 |
|  | Guanine nucleotide-binding protein subunit alpha-14 GN=GNA14 PE=2 SV=1 | O95837\|GNA14 | 4.1 | 9.58 | 3 | 0.9549 | 0.95 | 0.9338 |
|  | Amyloid beta A4 protein GN=APP PE=1 SV=3 | P05067\|A4 | 4.1 | 3.38 | 2 | 1.1536 | 0.75 | 2.3664 |
|  | 78 kDa glucose-regulated protein GN=HSPA5 PE=1 SV=2 | P11021\|GRP78 | 4.09 | 9.79 | 7 | 1.0300 | 0.94 | 1.1875 |
|  | GTP-binding nuclear protein Ran GN=RAN PE=1 SV=3 | P62826\|RAN | 4.09 | 8.80 | 2 | 1.1491 | 1.10 | 1.0027 |
|  | Phenylalanyl-tRNA synthetase beta chain GN=FARSB PE=1 SV=3 | Q9NSD9\|SYFB | 4.08 | 3.73 | 4 | 0.8378 | 0.94 | 0.9307 |
|  | Excitatory amino acid transporter 1 GN=SLC1A3 PE=1 SV=1 | P43003\|EAA1 | 4.08 | 5.90 | 5 | 0.6581 | 0.89 | 1.0351 |
|  | NADH-ubiquinone oxidoreductase 75 kDa subunit, mitochondrial GN=NDUFS1 PE=1 SV=3 | P28331\|NDUS1 | 4.06 | 3.71 | 2 | 1.0311 | 1.12 | 1.2219 |
|  | Glutamine synthetase GN=GLUL PE=1 SV=4 | P15104\|GLNA | 4.06 | 6.43 | 2 | 1.2347 | 1.02 | 0.8591 |
|  | 3-hydroxyacyl-CoA dehydrogenase type-2 GN=HSD17B10 PE=1 SV=3 | Q99714\|HCD2 | 4.05 | 11.88 | 2 | 0.9240 | 0.95 | 1.0790 |
|  | Mitochondrial inner membrane protein GN=IMMT PE=1 SV=1 | Q16891\|IMMT | 4.04 | 6.20 | 3 | 1.1552 | 1.00 | 0.9835 |
|  | Alpha-aminoadipic semialdehyde dehydrogenase GN=ALDH7A1 PE=1 SV=5 | P49419\|AL7A1 | 4.04 | 9.46 | 3 | 1.2558 | 1.16 | 0.9777 |
|  | Disks large homolog 4 GN=DLG4 PE=1 SV=3 | P78352\|DLG4 | 4.04 | 2.62 | 2 | 0.6734 | 0.67 | 0.6480 |
|  | Hyaluronan and proteoglycan link protein 2 GN=HAPLN2 PE=1 SV=1 | Q9GZV7\|HPLN2 | 4.04 | 7.06 | 2 | 2.9061 | 2.38 | 0.8968 |
|  | Ig alpha-1 chain C region GN=IGHA1 PE=1 SV=2 | P01876\|IGHA1 | 4.04 | 7.08 | 2 | 3.6300 | 2.08 | 1.6377 |
|  | Keratin, type II cytoskeletal 1 GN=KRT1 PE=1 SV=6 | P04264\|K2C1 | 4.03 | 6.06 | 4 | 1.4490 | 1.3384 | 1.3374 |
|  | Heat shock protein 105 kDa GN=HSPH1 PE=1 SV=1 | Q92598\|HS105 | 4.03 | 3.26 | 2 | 1.3632 | 1.13 | 0.9533 |
|  | 2-oxoglutarate dehydrogenase-like, mitochondrial GN=OGDHL PE=1 SV=3 | Q9ULD0\|OGDHL | 4.02 | 2.87 | 2 | 0.7209 | 0.82 | 0.7083 |
|  | Heterogeneous nuclear ribonucleoprotein Q GN=SYNCRIP PE=1 SV=2 | O60506\|HNRPQ | 4.02 | 3.85 | 2 | 0.9815 | 1.21 | 1.2208 |
|  | Pyridoxal phosphate phosphatase GN=PDXP PE=1 SV=2 | Q96GD0\|PLPP | 4.02 | 5.74 | 3 | 0.9011 | 0.78 | 0.6655 |
|  | ADP/ATP translocase 3 GN=SLC25A6 PE=1 SV=4 | P12236\|ADT3 | 4.01 | 19.13 | 5 | 0.5729 | 0.70 | 0.5934 |
|  | Electron transfer flavoprotein subunit alpha, mitochondrial GN=ETFA PE=1 SV=1 | P13804\|ETFA | 4.01 | 12.01 | 3 | 1.0882 | 1.05 | 0.6922 |
|  | Alpha-actinin-4 GN=ACTN4 PE=1 SV=2 | O43707\|ACTN4 | 4 | 6.15 | 5 | 1.0382 | 1.06 | 1.4624 |
|  | Voltage-dependent anion-selective channel protein 3 GN=VDAC3 PE=1 SV=1 | Q9Y277\|VDAC3 | 4 | 15.19 | 3 | 1.0008 | 0.99 | 1.3334 |
|  | Dihydropyrimidinase-related protein 4 GN=DPYSL4 PE=1 SV=2 | O14531\|DPYL4 | 4 | 7.69 | 3 | 0.9772 | 0.78 | 0.9291 |
|  | Heterogeneous nuclear ribonucleoprotein A1 GN=HNRNPA1 PE=1 SV=5 | P09651\|ROA1 | 4 | 10.48 | 2 | 1.0861 | 1.26 | 1.4092 |
|  | Catenin delta-2 GN=CTNND2 PE=1 SV=3 | Q9UQB3\|CTND2 | 4 | 1.22 | 2 | 1.0011 | 0.85 | 0.8352 |
|  | DnaJ homolog subfamily C member 11 GN=DNAJC11 PE=1 SV=2 | Q9NVH1\|DJC11 | 4 | 5.90 | 2 | 0.7973 | 0.67 | 0.6728 |
|  | Tyrosine-protein phosphatase non-receptor type 11 GN=PTPN11 PE=1 SV=2 | Q06124\|PTN11 | 4 | 5.36 | 2 | 1.1985 | 0.98 | 1.0962 |
|  | Cytochrome c oxidase subunit 4 isoform 1, mitochondrial GN=COX4I1 PE=1 SV=1 | P13073\|COX41 | 4 | 13.61 | 2 | 0.8079 | 1.14 | 0.7245 |
|  | Clusterin GN=CLU PE=1 SV=1 | P10909\|CLUS | 4 | 6.24 | 2 | 1.0220 | 1.12 | 2.3399 |
|  | Selenium-binding protein 1 GN=SELENBP1 PE=1 SV=2 | Q13228\|SBP1 | 4 | 7.63 | 2 | 1.1856 | 1.00 | 1.4257 |
|  | Adenylyl cyclase-associated protein 2 GN=CAP2 PE=1 SV=1 | P40123\|CAP2 | 4 | 6.50 | 2 | 0.7981 | 0.93 | 0.8329 |
|  | Stathmin GN=STMN1 PE=1 SV=3 | P16949\|STMN1 | 4 | 14.09 | 2 | 0.9497 | 1.23 | 0.7443 |
|  | Glutathione S-transferase Mu 1 GN=GSTM1 PE=1 SV=3 | P09488\|GSTM1 | 4 | 15.14 | 3 | 1.5541 | 1.67 | 2.2562 |
|  | NADH dehydrogenase [ubiquinone] iron-sulfur protein 8, mitochondrial GN=NDUFS8 PE=1 SV=1 | O00217\|NDUS8 | 4 | 9.52 | 2 | 0.6398 | 0.82 | 0.3840 |
|  | Ras-related protein Rap-1A GN=RAP1A PE=1 SV=1 | P62834\|RAP1A | 4 | 14.13 | 3 | 0.9958 | 1.03 | 1.2343 |
|  | ATP synthase subunit f, mitochondrial GN=ATP5J2 PE=1 SV=3 | P56134\|ATPK | 4 | 25.53 | 2 | 0.6287 | 0.74 | 0.8163 |
|  | Profilin-2 GN=PFN2 PE=1 SV=3 | P35080\|PROF2 | 4 | 23.57 | 2 | 1.1940 | 1.02 | 0.6531 |
|  | V-type proton ATPase subunit D GN=ATP6V1D PE=1 SV=1 | Q9Y5K8\|VATD | 3.99 | 13.77 | 2 | 0.7752 | 0.82 | 0.6936 |
|  | Actin-related protein 2/3 complex subunit 2 GN=ARPC2 PE=1 SV=1 | O15144\|ARPC2 | 3.92 | 8.00 | 2 | 1.0510 | 0.98 | 1.0696 |
|  | Thy-1 membrane glycoprotein GN=THY1 PE=1 SV=2 | P04216\|THY1 | 3.89 | 15.53 | 3 | 0.9080 | 1.03 | 1.1636 |
|  | Reticulon-3 GN=RTN3 PE=1 SV=2 | O95197\|RTN3 | 3.85 | 2.42 | 2 | 0.9790 | 0.94 | 1.2858 |
|  | Voltage-dependent anion-selective channel protein 2 GN=VDAC2 PE=1 SV=2 | P45880\|VDAC2 | 3.83 | 15.65 | 4 | 0.6319 | 0.87 | 1.0379 |
|  | Glutaminase kidney isoform, mitochondrial GN=GLS PE=1 SV=1 | O94925\|GLSK | 3.81 | 4.63 | 2 | 0.6386 | 0.85 | 0.6410 |
|  | 14-3-3 protein eta GN=YWHAH PE=1 SV=4 | Q04917\|1433F | 3.79 | 24.80 | 8 | 0.8694 | 0.81 | 0.9316 |
|  | Phosphatidylinositol-4-phosphate 5-kinase type-1 gamma GN=PIP5K1C PE=1 SV=2 | O60331\|PI51C | 3.78 | 4.94 | 2 | 0.9371 | 0.93 | 1.0184 |
|  | Laminin subunit gamma-1 GN=LAMC1 PE=1 SV=3 | P11047\|LAMC1 | 3.78 | 1.12 | 2 | 1.0980 | 1.28 | 2.5406 |
|  | Microtubule-associated protein 1A GN=MAP1A PE=1 SV=6 | P78559\|MAP1A | 3.71 | 2.57 | 4 | 0.9279 | 0.91 | 0.7791 |
|  | Cell division control protein 42 homolog GN=CDC42 PE=1 SV=2 | P60953\|CDC42 | 3.7 | 15.71 | 2 | 0.9167 | 0.94 | 0.9737 |
|  | Hemoglobin subunit delta GN=HBD PE=1 SV=2 | P02042\|HBD | 3.68 | 63.95 | 44 | 0.9366 | 1.25 | 1.4488 |
|  | Myosin-11 GN=MYH11 PE=1 SV=3 | P35749\|MYH11 | 3.68 | 2.99 | 3 | 0.9844 | 1.07 | 2.5666 |
|  | V-type proton ATPase 116 kDa subunit a isoform 1 GN=ATP6V0A1 PE=1 SV=3 | Q93050\|VPP1 | 3.68 | 3.34 | 2 | 0.7002 | 0.82 | 0.8554 |
|  | Dual specificity mitogen-activated protein kinase kinase 1 GN=MAP2K1 PE=1 SV=2 | Q02750\|MP2K1 | 3.68 | 8.65 | 2 | 0.9420 | 0.86 | 0.5405 |
|  | EH domain-containing protein 1 GN=EHD1 PE=1 SV=2 | Q9H4M9\|EHD1 | 3.67 | 2.06 | 1 | 0.9955 | 1.10 | 1.2073 |
|  | Neuronal-specific septin-3 GN=SEPT3 PE=1 SV=3 | Q9UH03\|SEPT3 | 3.67 | 6.15 | 2 | 1.1159 | 1.02 | 0.9076 |
|  | Abhydrolase domain-containing protein 10, mitochondrial GN=ABHD10 PE=1 SV=1 | Q9NUJ1\|ABHDA | 3.66 | 10.78 | 2 | 0.9570 | 0.97 | 0.6387 |
|  | Superoxide dismutase [Cu-Zn] GN=SOD1 PE=1 SV=2 | P00441\|SODC | 3.65 | 20.13 | 4 | 1.5118 | 1.15 | 1.1851 |
|  | cAMP-dependent protein kinase type II-beta regulatory subunit GN=PRKAR2B PE=1 SV=3 | P31323\|KAP3 | 3.6 | 12.20 | 4 | 0.8438 | 0.89 | 0.8347 |
|  | Prolargin GN=PRELP PE=1 SV=1 | P51888\|PRELP | 3.58 | 6.28 | 6 | 0.8507 | 1.29 | 3.4733 |
|  | Rho GDP-dissociation inhibitor 1 GN=ARHGDIA PE=1 SV=3 | P52565\|GDIR1 | 3.57 | 18.63 | 3 | 1.0915 | 0.81 | 0.9149 |
|  | Kinesin heavy chain isoform 5C GN=KIF5C PE=1 SV=1 | O60282\|KIF5C | 3.56 | 2.30 | 2 | 0.7687 | 0.68 | 0.6260 |
|  | Thioredoxin-dependent peroxide reductase, mitochondrial GN=PRDX3 PE=1 SV=3 | P30048\|PRDX3 | 3.47 | 16.80 | 4 | 1.0456 | 1.21 | 1.1626 |
|  | Copine-5 GN=CPNE5 PE=1 SV=2 | Q9HCH3\|CPNE5 | 3.43 | 4.72 | 2 | 0.7261 | 0.80 | 0.7901 |
|  | Dipeptidyl aminopeptidase-like protein 6 GN=DPP6 PE=1 SV=2 | P42658\|DPP6 | 3.42 | 2.54 | 2 | 0.9274 | 1.04 | 0.9773 |
|  | Myosin light polypeptide 6 GN=MYL6 PE=1 SV=2 | P60660\|MYL6 | 3.42 | 16.56 | 3 | 1.0984 | 1.23 | 2.9975 |
|  | Ras-related protein Rab-14 GN=RAB14 PE=1 SV=4 | P61106\|RAB14 | 3.4 | 14.42 | 2 | 0.9609 | 1.04 | 0.9005 |
|  | UMP-CMP kinase GN=CMPK1 PE=1 SV=3 | P30085\|KCY | 3.4 | 20.92 | 4 | 0.6162 | 0.62 | 0.4612 |
|  | Tubulin alpha-1A chain GN=TUBA1A PE=1 SV=1 | Q71U36\|TBA1A | 3.38 | 50.55 | 84 | 0.7434 | 0.59 | 0.4853 |
|  | Cell adhesion molecule 3 GN=CADM3 PE=1 SV=1 | Q8N126\|CADM3 | 3.38 | 7.54 | 3 | 0.9280 | 1.19 | 0.9104 |
|  | Transgelin-3 GN=TAGLN3 PE=1 SV=2 | Q9UI15\|TAGL3 | 3.37 | 10.05 | 2 | 1.0417 | 0.97 | 0.7362 |
|  | Alpha-1-syntrophin GN=SNTA1 PE=1 SV=1 | Q13424\|SNTA1 | 3.34 | 6.73 | 2 | 0.9576 | 1.05 | 1.0627 |
|  | Phosphoserine aminotransferase GN=PSAT1 PE=1 SV=2 | Q9Y617\|SERC | 3.34 | 6.22 | 2 | 1.2582 | 1.15 | 1.3849 |
|  | NADH dehydrogenase [ubiquinone] 1 alpha subcomplex subunit 9, mitochondrial GN=NDUFA9 PE=1 SV=2 | Q16795\|NDUA9 | 3.33 | 8.22 | 2 | 0.8319 | 1.00 | 0.8835 |
|  | Collagen alpha-2(VI) chain GN=COL6A2 PE=1 SV=4 | P12110\|CO6A2 | 3.32 | 2.06 | 2 | 0.9415 | 1.41 | 3.6496 |
|  | AP-2 complex subunit mu GN=AP2M1 PE=1 SV=2 | Q96CW1\|AP2M1 | 3.32 | 6.21 | 2 | 0.7108 | 0.79 | 0.6662 |
|  | V-type proton ATPase subunit F GN=ATP6V1F PE=1 SV=2 | Q16864\|VATF | 3.27 | 34.45 | 4 | 0.8326 | 0.79 | 0.5185 |
|  | Astrocytic phosphoprotein PEA-15 GN=PEA15 PE=1 SV=2 | Q15121\|PEA15 | 3.25 | 26.92 | 2 | 0.7989 | 0.63 | 0.5724 |
|  | Proteasome subunit beta type-1 GN=PSMB1 PE=1 SV=2 | P20618\|PSB1 | 3.24 | 13.28 | 2 | 1.0293 | 0.95 | 1.2841 |
|  | Cytochrome c oxidase subunit 2 GN=MT-CO2 PE=1 SV=1 | P00403\|COX2 | 3.2 | 8.81 | 1 | 0.5634 | 0.71 | 0.7227 |
|  | Ubiquitin carboxyl-terminal hydrolase 5 GN=USP5 PE=1 SV=2 | P45974\|UBP5 | 3.19 | 5.71 | 3 | 0.6762 | 0.81 | 0.8099 |
|  | Poly(rC)-binding protein 2 GN=PCBP2 PE=1 SV=1 | Q15366\|PCBP2 | 3.19 | 9.31 | 2 | 1.0047 | 1.01 | 1.1699 |
|  | Phospholipid hydroperoxide glutathione peroxidase, mitochondrial GN=GPX4 PE=1 SV=3 | P36969\|GPX4 | 3.19 | 13.71 | 2 | 1.2236 | 0.96 | 1.1197 |
|  | Dihydrolipoyllysine-residue succinyltransferase component of 2-oxoglutarate dehydrogenase complex, mitochondrial GN=DLST PE=1 SV=4 | P36957\|ODO2 | 3.18 | 5.52 | 2 | 0.8094 | 0.80 | 0.6834 |
|  | Vacuolar protein sorting-associated protein 35 GN=VPS35 PE=1 SV=2 | Q96QK1\|VPS35 | 3.17 | 3.14 | 2 | 0.9460 | 0.95 | 1.2890 |
|  | Thiomorpholine-carboxylate dehydrogenase GN=CRYM PE=1 SV=1 | Q14894\|CRYM | 3.15 | 11.46 | 2 | 0.9022 | 0.89 | 0.7946 |
|  | Alpha-2-macroglobulin GN=A2M PE=1 SV=3 | P01023\|A2MG | 3.13 | 1.97 | 2 | 1.8732 | 1.17 | 1.9612 |
|  | Hydroxyacylglutathione hydrolase, mitochondrial GN=HAGH PE=1 SV=2 | Q16775\|GLO2 | 3.11 | 7.79 | 2 | 0.7053 | 0.56 | 0.5474 |
|  | Putative adenosylhomocysteinase 2 GN=AHCYL1 PE=1 SV=2 | O43865\|SAHH2 | 3.11 | 3.77 | 2 | 1.1723 | 1.05 | 1.4840 |
|  | Oxidation resistance protein 1 GN=OXR1 PE=1 SV=2 | Q8N573\|OXR1 | 3.05 | 1.83 | 3 | 1.0583 | 0.95 | 1.0451 |
|  | Platelet-activating factor acetylhydrolase IB subunit beta GN=PAFAH1B2 PE=1 SV=1 | P68402\|PA1B2 | 3.01 | 12.23 | 4 | 1.5210 | 1.11 | 0.9214 |
|  | Oligodendrocyte-myelin glycoprotein GN=OMG PE=1 SV=2 | P23515\|OMGP | 2.98 | 4.09 | 2 | 1.3597 | 1.18 | 0.8652 |
|  | Calcium/calmodulin-dependent protein kinase type II subunit gamma GN=CAMK2G PE=1 SV=3 | Q13555\|KCC2G | 2.96 | 11.65 | 5 | 0.8458 | 0.98 | 0.8688 |
|  | Protein S100-B GN=S100B PE=1 SV=2 | P04271\|S100B | 2.96 | 16.30 | 9 | 1.4744 | 1.19 | 0.9101 |
|  | LanC-like protein 1 GN=LANCL1 PE=1 SV=1 | O43813\|LANC1 | 2.92 | 6.27 | 2 | 1.2507 | 1.20 | 1.3638 |
|  | NEDD8 GN=NEDD8 PE=1 SV=1 | Q15843\|NEDD8 | 2.9 | 25.93 | 3 | 0.8245 | 0.82 | 0.4960 |
|  | Succinate-semialdehyde dehydrogenase, mitochondrial GN=ALDH5A1 PE=1 SV=2 | P51649\|SSDH | 2.89 | 7.10 | 2 | 0.9822 | 0.98 | 1.0551 |
|  | Dynamin-like 120 kDa protein, mitochondrial GN=OPA1 PE=1 SV=3 | O60313\|OPA1 | 2.86 | 2.60 | 3 | 0.7988 | 0.78 | 0.8409 |
|  | Tripeptidyl-peptidase 1 GN=TPP1 PE=1 SV=2 | O14773\|TPP1 | 2.85 | 4.62 | 3 | 1.1547 | 1.13 | 1.6036 |
|  | Cytosolic acyl coenzyme A thioester hydrolase GN=ACOT7 PE=1 SV=3 | O00154\|BACH | 2.83 | 18.68 | 5 | 0.9026 | 0.87 | 0.7853 |
|  | Galectin-1 GN=LGALS1 PE=1 SV=2 | P09382\|LEG1 | 2.82 | 17.78 | 2 | 1.0199 | 1.18 | 1.5679 |
|  | Microtubule-associated proteins 1A/1B light chain 3A GN=MAP1LC3A PE=1 SV=2 | Q9H492\|MLP3A | 2.77 | 17.36 | 2 | 0.9906 | 0.95 | 0.6766 |
|  | Cytochrome b-c1 complex subunit 7 GN=UQCRB PE=1 SV=2 | P14927\|QCR7 | 2.72 | 15.32 | 2 | 0.9740 | 1.12 | 0.9348 |
|  | Contactin-associated protein 1 GN=CNTNAP1 PE=1 SV=1 | P78357\|CNTP1 | 2.71 | 1.08 | 1 | 0.8308 | 0.98 | 0.9660 |
|  | Cytoplasmic FMR1-interacting protein 1 GN=CYFIP1 PE=1 SV=1 | Q7L576\|CYFP1 | 2.7 | 1.92 | 2 | 1.0723 | 1.11 | 1.5971 |
|  | Protein disulfide-isomerase GN=P4HB PE=1 SV=3 | P07237\|PDIA1 | 2.69 | 6.30 | 2 | 0.8099 | 0.87 | 1.6112 |
|  | Isochorismatase domain-containing protein 2, mitochondrial GN=ISOC2 PE=1 SV=1 | Q96AB3\|ISOC2 | 2.69 | 20.00 | 2 | 1.0555 | 0.83 | 1.0604 |
|  | Regulator of microtubule dynamics protein 3 GN=FAM82A2 PE=1 SV=2 | Q96TC7\|RMD3 | 2.63 | 7.02 | 2 | 0.6741 | 0.71 | 0.9335 |
|  | Guanine deaminase GN=GDA PE=1 SV=1 | Q9Y2T3\|GUAD | 2.63 | 7.93 | 3 | 1.0369 | 0.89 | 0.8337 |
|  | Phosphatidylinositol-5-phosphate 4-kinase type-2 beta GN=PIP4K2B PE=1 SV=1 | P78356\|PI42B | 2.63 | 1.92 | 1 | 1.1271 | 1.04 | 0.7491 |
|  | Ezrin GN=EZR PE=1 SV=4 | P15311\|EZRI | 2.62 | 1.54 | 1 | 1.1121 | 0.92 | 1.1643 |
|  | AP-2 complex subunit sigma GN=AP2S1 PE=1 SV=2 | P53680\|AP2S1 | 2.62 | 8.45 | 1 | 0.9356 | 1.23 | 1.1204 |
|  | Serine/threonine-protein kinase PAK 1 GN=PAK1 PE=1 SV=2 | Q13153\|PAK1 | 2.6 | 6.79 | 3 | 1.1166 | 0.96 | 0.7780 |
|  | Acyl-coenzyme A thioesterase 13 GN=ACOT13 PE=1 SV=1 | Q9NPJ3\|ACO13 | 2.59 | 9.29 | 1 | 1.2757 | 0.95 | 0.7143 |
|  | Vacuolar protein sorting-associated protein 29 GN=VPS29 PE=1 SV=1 | Q9UBQ0\|VPS29 | 2.57 | 25.82 | 3 | 0.8789 | 0.95 | 1.1346 |
|  | Alpha-1-acid glycoprotein 2 GN=ORM2 PE=1 SV=2 | P19652\|A1AG2 | 2.57 | 9.45 | 2 | 4.3706 | 1.75 | 1.8590 |
|  | Atlastin-1 GN=ATL1 PE=1 SV=1 | Q8WXF7\|ATLA1 | 2.56 | 2.33 | 1 | 0.7771 | 0.90 | 1.0713 |
|  | Rab GDP dissociation inhibitor alpha GN=GDI1 PE=1 SV=2 | P31150\|GDIA | 2.54 | 15.66 | 10 | 1.2043 | 1.06 | 0.8832 |
|  | WD repeat-containing protein 1 GN=WDR1 PE=1 SV=4 | O75083\|WDR1 | 2.53 | 3.63 | 1 | 0.8866 | 0.95 | 1.4998 |
|  | 10 kDa heat shock protein, mitochondrial GN=HSPE1 PE=1 SV=2 | P61604\|CH10 | 2.49 | 13.73 | 1 | 0.9265 | 0.93 | 0.8359 |
|  | NADH dehydrogenase [ubiquinone] 1 alpha subcomplex subunit 13 GN=NDUFA13 PE=1 SV=3 | Q9P0J0\|NDUAD | 2.48 | 9.03 | 1 | 0.5320 | 0.61 | 0.4960 |
|  | Eukaryotic initiation factor 4A-II GN=EIF4A2 PE=1 SV=2 | Q14240\|IF4A2 | 2.47 | 3.69 | 1 | 0.9867 | 1.07 | 1.0775 |
|  | Ubiquitin thioesterase OTUB1 GN=OTUB1 PE=1 SV=2 | Q96FW1\|OTUB1 | 2.47 | 3.69 | 1 | 1.1208 | 0.95 | 0.9477 |
|  | Poly(rC)-binding protein 1 GN=PCBP1 PE=1 SV=2 | Q15365\|PCBP1 | 2.44 | 5.90 | 2 | 0.8951 | 0.96 | 0.9553 |
|  | Fatty acid-binding protein, heart GN=FABP3 PE=1 SV=4 | P05413\|FABPH | 2.44 | 6.77 | 1 | 1.2400 | 0.93 | 0.8671 |
|  | T-complex protein 1 subunit gamma GN=CCT3 PE=1 SV=4 | P49368\|TCPG | 2.43 | 2.20 | 1 | 1.0510 | 0.96 | 0.9051 |
|  | Ras-related protein Rab-6A GN=RAB6A PE=1 SV=3 | P20340\|RAB6A | 2.43 | 5.77 | 1 | 1.0938 | 0.97 | 1.1815 |
|  | Peptidyl-prolyl cis-trans isomerase NIMA-interacting 1 GN=PIN1 PE=1 SV=1 | Q13526\|PIN1 | 2.41 | 11.66 | 2 | 0.9662 | 0.97 | 1.1189 |
|  | Kelch repeat and BTB domain-containing protein 11 GN=KBTBD11 PE=1 SV=1 | O94819\|KBTBB | 2.37 | 2.25 | 1 | 1.1161 | 1.10 | 1.4688 |
|  | tRNA-splicing ligase RtcB homolog GN=C22orf28 PE=1 SV=1 | Q9Y3I0\|RTCB | 2.37 | 4.95 | 2 | 1.0991 | 1.34 | 0.6763 |
|  | Hippocalcin-like protein 1 GN=HPCAL1 PE=1 SV=3 | P37235\|HPCL1 | 2.36 | 8.81 | 2 | 0.8320 | 1.04 | 1.0231 |
|  | S-phase kinase-associated protein 1 GN=SKP1 PE=1 SV=2 | P63208\|SKP1 | 2.33 | 11.66 | 2 | 0.9913 | 0.92 | 0.6984 |
|  | Heterogeneous nuclear ribonucleoprotein M GN=HNRNPM PE=1 SV=3 | P52272\|HNRPM | 2.32 | 2.05 | 1 | 0.8297 | 0.76 | 0.9590 |
|  | Leukocyte surface antigen CD47 GN=CD47 PE=1 SV=1 | Q08722\|CD47 | 2.29 | 3.41 | 1 | 0.7277 | 0.99 | 0.8596 |
|  | Lumican GN=LUM PE=1 SV=2 | P51884\|LUM | 2.28 | 3.25 | 1 | 0.9619 | 1.61 | 3.0607 |
|  | ATP synthase subunit gamma, mitochondrial GN=ATP5C1 PE=1 SV=1 | P36542\|ATPG | 2.27 | 7.38 | 2 | 0.8997 | 0.89 | 0.7992 |
|  | ATP synthase subunit b, mitochondrial GN=ATP5F1 PE=1 SV=2 | P24539\|AT5F1 | 2.26 | 5.86 | 1 | 0.8478 | 1.13 | 1.2456 |
|  | Protein S100-A9 GN=S100A9 PE=1 SV=1 | P06702\|S10A9 | 2.25 | 31.58 | 3 | 1.3845 | 1.16 | 4.7455 |
|  | Cystatin-B GN=CSTB PE=1 SV=2 | P04080\|CYTB | 2.25 | 12.24 | 1 | 1.4622 | 1.18 | 0.9657 |
|  | Lactoylglutathione lyase GN=GLO1 PE=1 SV=4 | Q04760\|LGUL | 2.23 | 15.76 | 2 | 1.2289 | 1.08 | 1.2177 |
|  | Succinate dehydrogenase [ubiquinone] flavoprotein subunit, mitochondrial GN=SDHA PE=1 SV=2 | P31040\|DHSA | 2.22 | 3.92 | 2 | 0.7706 | 0.98 | 1.1113 |
|  | Beta-soluble NSF attachment protein GN=NAPB PE=1 SV=2 | Q9H115\|SNAB | 2.22 | 3.69 | 1 | 0.7512 | 0.72 | 0.4385 |
|  | ATP synthase subunit e, mitochondrial GN=ATP5I PE=1 SV=2 | P56385\|ATP5I | 2.21 | 20.29 | 2 | 0.9051 | 0.83 | 0.6542 |
|  | Protein S100-A1 GN=S100A1 PE=1 SV=2 | P23297\|S10A1 | 2.21 | 38.30 | 3 | 1.2089 | 1.30 | 1.4067 |
|  | S-formylglutathione hydrolase GN=ESD PE=1 SV=2 | P10768\|ESTD | 2.2 | 7.45 | 1 | 1.3183 | 1.01 | 1.2673 |
|  | Kinesin-like protein KIF21A GN=KIF21A PE=1 SV=2 | Q7Z4S6\|KI21A | 2.18 | 0.66 | 1 | 0.9407 | 0.97 | 2.3755 |
|  | Neural cell adhesion molecule L1 GN=L1CAM PE=1 SV=2 | P32004\|L1CAM | 2.18 | 1.99 | 2 | 0.8898 | 1.17 | 1.5507 |
|  | ATP-dependent RNA helicase A GN=DHX9 PE=1 SV=4 | Q08211\|DHX9 | 2.17 | 1.02 | 1 | 1.0711 | 0.94 | 1.0779 |
|  | T-complex protein 1 subunit delta GN=CCT4 PE=1 SV=4 | P50991\|TCPD | 2.16 | 2.41 | 1 | 1.1249 | 1.26 | 1.4125 |
|  | Ribose-phosphate pyrophosphokinase 1 GN=PRPS1 PE=1 SV=2 | P60891\|PRPS1 | 2.16 | 5.35 | 1 | 1.0879 | 0.87 | 0.8618 |
|  | CaM kinase-like vesicle-associated protein GN=CAMKV PE=2 SV=2 | Q8NCB2\|CAMKV | 2.15 | 2.79 | 1 | 0.8531 | 1.11 | 1.2306 |
|  | Flotillin-2 GN=FLOT2 PE=1 SV=2 | Q14254\|FLOT2 | 2.13 | 2.80 | 1 | 1.0524 | 0.96 | 1.2291 |
|  | Prolyl endopeptidase-like GN=PREPL PE=1 SV=1 | Q4J6C6\|PPCEL | 2.13 | 1.93 | 1 | 1.1883 | 1.16 | 1.3736 |
|  | Transaldolase GN=TALDO1 PE=1 SV=2 | P37837\|TALDO | 2.13 | 3.26 | 1 | 1.7269 | 1.35 | 0.9308 |
|  | Stress-induced-phosphoprotein 1 GN=STIP1 PE=1 SV=1 | P31948\|STIP1 | 2.12 | 1.84 | 1 | 1.2144 | 1.00 | 0.6604 |
|  | Carboxymethylenebutenolidase homolog GN=CMBL PE=1 SV=1 | Q96DG6\|CMBL | 2.12 | 6.94 | 2 | 0.8846 | 1.04 | 1.1741 |
|  | THO complex subunit 4 GN=THOC4 PE=1 SV=3 | Q86V81\|THOC4 | 2.11 | 4.28 | 1 | 1.1745 | 1.19 | 1.0487 |
|  | Beta-adducin GN=ADD2 PE=1 SV=3 | P35612\|ADDB | 2.11 | 1.52 | 1 | 0.8693 | 0.85 | 0.8968 |
|  | Glycogen phosphorylase, muscle form GN=PYGM PE=1 SV=6 | P11217\|PYGM | 2.1 | 1.43 | 1 | 0.8910 | 1.07 | 1.8345 |
|  | T-complex protein 1 subunit theta GN=CCT8 PE=1 SV=4 | P50990\|TCPQ | 2.1 | 2.74 | 1 | 0.9686 | 1.14 | 0.7871 |
|  | Phospholysine phosphohistidine inorganic pyrophosphate phosphatase GN=LHPP PE=1 SV=2 | Q9H008\|LHPP | 2.1 | 12.59 | 3 | 1.1405 | 0.96 | 0.8028 |
|  | Protein bassoon GN=BSN PE=1 SV=4 | Q9UPA5\|BSN | 2.09 | 0.28 | 1 | 0.7789 | 0.89 | 0.6947 |
|  | Synemin GN=SYNM PE=1 SV=2 | O15061\|SYNEM | 2.09 | 0.58 | 1 | 1.4521 | 1.44 | 2.1093 |
|  | Isocitrate dehydrogenase [NAD] subunit beta, mitochondrial GN=IDH3B PE=1 SV=2 | O43837\|IDH3B | 2.09 | 2.60 | 2 | 0.9914 | 0.92 | 1.1479 |
|  | A-kinase anchor protein 12 GN=AKAP12 PE=1 SV=4 | Q02952\|AKA12 | 2.08 | 0.84 | 1 | 0.6611 | 0.70 | 0.6518 |
|  | Catenin beta-1 GN=CTNNB1 PE=1 SV=1 | P35222\|CTNB1 | 2.07 | 1.54 | 1 | 1.0528 | 0.94 | 1.1223 |
|  | Active breakpoint cluster region-related protein GN=ABR PE=2 SV=2 | Q12979\|ABR | 2.07 | 1.40 | 1 | 0.8186 | 0.95 | 0.8306 |
|  | Prostaglandin E synthase 2 GN=PTGES2 PE=1 SV=1 | Q9H7Z7\|PGES2 | 2.07 | 11.14 | 3 | 0.9049 | 0.98 | 1.0346 |
|  | Septin-2 GN=SEPT2 PE=1 SV=1 | Q15019\|SEPT2 | 2.06 | 11.91 | 3 | 0.9625 | 0.91 | 0.8958 |
|  | Talin-2 GN=TLN2 PE=1 SV=4 | Q9Y4G6\|TLN2 | 2.06 | 0.43 | 1 | 1.0889 | 1.01 | 0.6683 |
|  | IQ motif and SEC7 domain-containing protein 1 GN=IQSEC1 PE=1 SV=1 | Q6DN90\|IQEC1 | 2.06 | 1.77 | 1 | 1.0098 | 1.04 | 1.2508 |
|  | ATP synthase subunit delta, mitochondrial GN=ATP5D PE=1 SV=2 | P30049\|ATPD | 2.06 | 8.33 | 1 | 0.8392 | 0.86 | 0.6923 |
|  | Alpha-1-antichymotrypsin GN=SERPINA3 PE=1 SV=2 | P01011\|AACT | 2.05 | 4.96 | 2 | 1.9277 | 1.19 | 1.3464 |
|  | PH and SEC7 domain-containing protein 3 GN=PSD3 PE=1 SV=2 | Q9NYI0\|PSD3 | 2.05 | 0.95 | 1 | 1.0426 | 1.54 | 0.7877 |
|  | N-terminal EF-hand calcium-binding protein 2 GN=NECAB2 PE=1 SV=1 | Q7Z6G3\|NECA2 | 2.05 | 2.85 | 1 | 0.5403 | 0.46 | 0.6219 |
|  | Gamma-adducin GN=ADD3 PE=1 SV=1 | Q9UEY8\|ADDG | 2.05 | 1.42 | 1 | 1.0246 | 1.33 | 2.5510 |
|  | Transforming protein RhoA GN=RHOA PE=1 SV=1 | P61586\|RHOA | 2.05 | 8.81 | 3 | 0.7098 | 0.70 | 0.7342 |
|  | Gap junction alpha-1 protein GN=GJA1 PE=1 SV=2 | P17302\|CXA1 | 2.04 | 3.66 | 1 | 0.7244 | 1.49 | 2.7275 |
|  | Protein NDRG1 GN=NDRG1 PE=1 SV=1 | Q92597\|NDRG1 | 2.04 | 4.06 | 2 | 0.8714 | 0.86 | 1.0738 |
|  | Serine/threonine-protein phosphatase 2A activator GN=PPP2R4 PE=1 SV=3 | Q15257\|PTPA | 2.04 | 2.79 | 1 | 1.1953 | 0.92 | 0.6014 |
|  | V-type proton ATPase subunit C 1 GN=ATP6V1C1 PE=1 SV=4 | P21283\|VATC1 | 2.04 | 4.19 | 1 | 1.0928 | 1.06 | 1.5671 |
|  | 14-3-3 protein theta GN=YWHAQ PE=1 SV=1 | P27348\|1433T | 2.03 | 21.22 | 7 | 1.3893 | 1.40 | 1.3363 |
|  | Plasma membrane calcium-transporting ATPase 2 GN=ATP2B2 PE=1 SV=2 | Q01814\|AT2B2 | 2.03 | 0.88 | 1 | 0.8505 | 1.16 | 0.9299 |
|  | Actin-binding protein anillin GN=ANLN PE=1 SV=2 | Q9NQW6\|ANLN | 2.03 | 1.51 | 1 | 1.3907 | 0.75 | 0.4118 |
|  | Ras GTPase-activating protein SynGAP GN=SYNGAP1 PE=1 SV=4 | Q96PV0\|SYGP1 | 2.03 | 1.27 | 1 | 1.0765 | 0.76 | 0.6146 |
|  | Vacuolar protein sorting-associated protein 33A GN=VPS33A PE=1 SV=1 | Q96AX1\|VP33A | 2.03 | 1.68 | 1 | 2.6462 | 2.71 | 5.9285 |
|  | Integrin-linked protein kinase GN=ILK PE=1 SV=2 | Q13418\|ILK | 2.03 | 3.10 | 1 | 0.6032 | 0.84 | 1.5271 |
|  | Sideroflexin-1 GN=SFXN1 PE=1 SV=4 | Q9H9B4\|SFXN1 | 2.03 | 2.80 | 1 | 0.4761 | 0.73 | 0.5233 |
|  | Proteasome subunit alpha type-3 GN=PSMA3 PE=1 SV=2 | P25788\|PSA3 | 2.03 | 5.49 | 1 | 0.9668 | 0.92 | 0.9980 |
|  | 6-phosphofructokinase type C GN=PFKP PE=1 SV=2 | Q01813\|K6PP | 2.02 | 3.32 | 4 | 1.2823 | 1.12 | 0.8233 |
|  | Gamma-synuclein GN=SNCG PE=1 SV=2 | O76070\|SYUG | 2.02 | 19.69 | 3 | 0.8539 | 0.77 | 0.8963 |
|  | Leucine-rich PPR motif-containing protein, mitochondrial GN=LRPPRC PE=1 SV=3 | P42704\|LPPRC | 2.02 | 1.29 | 1 | 1.1543 | 0.94 | 1.1365 |
|  | Immunity-related GTPase family Q protein GN=IRGQ PE=1 SV=1 | Q8WZA9\|IRGQ | 2.02 | 2.25 | 1 | 0.8859 | 0.61 | 0.7942 |
|  | 60S ribosomal protein L15 GN=RPL15 PE=1 SV=2 | P61313\|RL15 | 2.02 | 5.88 | 1 | 1.0620 | 1.53 | 0.7624 |
|  | Annexin A1 GN=ANXA1 PE=1 SV=2 | P04083\|ANXA1 | 2.02 | 4.33 | 1 | 1.0193 | 1.20 | 2.3245 |
|  | cAMP-dependent protein kinase catalytic subunit alpha GN=PRKACA PE=1 SV=2 | P17612\|KAPCA | 2.02 | 3.42 | 1 | 1.1485 | 1.12 | 1.2767 |
|  | Phytanoyl-CoA hydroxylase-interacting protein GN=PHYHIP PE=1 SV=1 | Q92561\|PHYIP | 2.02 | 2.73 | 1 | 0.8970 | 1.08 | 0.9356 |
|  | Heat shock-related 70 kDa protein 2 GN=HSPA2 PE=1 SV=1 | P54652\|HSP72 | 2.01 | 20.97 | 14 | 2.3460 | 1.66 | 1.0783 |
|  | AP-2 complex subunit alpha-2 GN=AP2A2 PE=1 SV=2 | O94973\|AP2A2 | 2.01 | 5.43 | 4 | 0.7141 | 0.69 | 0.6770 |
|  | Isovaleryl-CoA dehydrogenase, mitochondrial GN=IVD PE=1 SV=1 | P26440\|IVD | 2.01 | 5.91 | 2 | 0.7841 | 0.95 | 0.6950 |
|  | Solute carrier family 12 member 5 GN=SLC12A5 PE=2 SV=3 | Q9H2X9\|S12A5 | 2.01 | 1.93 | 1 | 0.7063 | 0.71 | 0.5937 |
|  | General vesicular transport factor p115 GN=USO1 PE=1 SV=2 | O60763\|USO1 | 2.01 | 1.98 | 1 | 0.7988 | 0.80 | 1.1523 |
|  | IQ motif and SEC7 domain-containing protein 2 GN=IQSEC2 PE=1 SV=1 | Q5JU85\|IQEC2 | 2.01 | 1.15 | 1 | 0.9428 | 0.82 | 1.0953 |
|  | Polyadenylate-binding protein 1 GN=PABPC1 PE=1 SV=2 | P11940\|PABP1 | 2.01 | 2.20 | 1 | 1.1088 | 0.96 | 1.0817 |
|  | Ganglioside-induced differentiation-associated protein 1-like 1 GN=GDAP1L1 PE=2 SV=2 | Q96MZ0\|GD1L1 | 2.01 | 3.54 | 1 | 0.8073 | 1.13 | 1.0444 |
|  | Complement component 1 Q subcomponent-binding protein, mitochondrial GN=C1QBP PE=1 SV=1 | Q07021\|C1QBP | 2.01 | 10.64 | 2 | 0.9178 | 0.93 | 0.9265 |
|  | Ornithine aminotransferase, mitochondrial GN=OAT PE=1 SV=1 | P04181\|OAT | 2.01 | 3.64 | 1 | 1.0611 | 0.72 | 1.0476 |
|  | Vitronectin GN=VTN PE=1 SV=1 | P04004\|VTNC | 2.01 | 3.35 | 1 | 1.3112 | 1.38 | 3.4520 |
|  | Cytochrome c oxidase subunit 7A2, mitochondrial GN=COX7A2 PE=1 SV=1 | P14406\|CX7A2 | 2.01 | 12.05 | 1 | 0.8471 | 1.04 | 0.7009 |
|  | Phosphate carrier protein, mitochondrial GN=SLC25A3 PE=1 SV=2 | Q00325\|MPCP | 2.01 | 3.31 | 1 | 0.2290 | 0.79 | 1.0425 |
|  | Guanine nucleotide-binding protein subunit beta-5 GN=GNB5 PE=1 SV=2 | O14775\|GBB5 | 2.01 | 3.04 | 1 | 0.8883 | 0.87 | 0.6930 |
|  | V-type proton ATPase subunit G 2 GN=ATP6V1G2 PE=1 SV=1 | O95670\|VATG2 | 2.01 | 7.63 | 1 | 1.1678 | 1.45 | 0.5790 |
|  | Hyaluronan and proteoglycan link protein 1 GN=HAPLN1 PE=2 SV=2 | P10915\|HPLN1 | 2.01 | 5.93 | 1 | 0.9138 | 0.86 | 0.9451 |
|  | CD166 antigen GN=ALCAM PE=1 SV=2 | Q13740\|CD166 | 2.01 | 2.40 | 1 | 0.9067 | 0.94 | 1.2970 |
|  | Peptidyl-prolyl cis-trans isomerase FKBP1A GN=FKBP1A PE=1 SV=2 | P62942\|FKB1A | 2.01 | 25.00 | 2 | 0.8846 | 0.78 | 1.0131 |
|  | Actin, alpha skeletal muscle GN=ACTA1 PE=1 SV=1 | P68133\|ACTS | 2 | 50.40 | 37 | 1.2727 | 1.38 | 2.0910 |
|  | Histone H2A.J GN=H2AFJ PE=1 SV=1 | Q9BTM1\|H2AJ | 2 | 49.61 | 11 | 0.7496 | 1.04 | 0.4627 |
|  | ADP/ATP translocase 2 GN=SLC25A5 PE=1 SV=7 | P05141\|ADT2 | 2 | 19.13 | 6 | 0.7669 | 1.04 | 0.9279 |
|  | Guanine nucleotide-binding protein G(k) subunit alpha GN=GNAI3 PE=1 SV=3 | P08754\|GNAI3 | 2 | 15.25 | 5 | 0.6273 | 0.80 | 0.5938 |
|  | Putative elongation factor 1-alpha-like 3 GN=EEF1A1P5 PE=5 SV=1 | Q5VTE0\|EF1A3 | 2 | 10.82 | 4 | 1.2282 | 1.08 | 1.4314 |
|  | Ras-related protein Rab-3C GN=RAB3C PE=2 SV=1 | Q96E17\|RAB3C | 2 | 16.30 | 3 | 0.5893 | 0.63 | 0.5936 |
|  | Ig gamma-2 chain C region GN=IGHG2 PE=1 SV=2 | P01859\|IGHG2 | 2 | 10.12 | 3 | 6.3968 | 1.93 | 2.7215 |
|  | 2-oxoglutarate dehydrogenase, mitochondrial GN=OGDH PE=1 SV=3 | Q02218\|ODO1 | 2 | 3.32 | 2 | 0.6827 | 0.91 | 0.7694 |
|  | Isocitrate dehydrogenase [NADP] cytoplasmic GN=IDH1 PE=1 SV=2 | O75874\|IDHC | 2 | 5.31 | 2 | 1.5062 | 0.95 | 1.4583 |
|  | Alpha-centractin GN=ACTR1A PE=1 SV=1 | P61163\|ACTZ | 2 | 4.26 | 1 | 0.9935 | 1.10 | 1.0720 |
|  | Ubiquilin-2 GN=UBQLN2 PE=1 SV=2 | Q9UHD9\|UBQL2 | 2 | 2.40 | 1 | 0.6722 | 0.76 | 0.7696 |
|  | Aspartyl-tRNA synthetase, cytoplasmic GN=DARS PE=1 SV=2 | P14868\|SYDC | 2 | 2.00 | 1 | 0.9489 | 0.98 | 1.0965 |
|  | [Pyruvate dehydrogenase [acetyl-transferring]]-phosphatase 1, mitochondrial GN=PDP1 PE=1 SV=3 | Q9P0J1\|PDP1 | 2 | 2.79 | 1 | 0.7742 | 1.05 | 0.9897 |
|  | Limbic system-associated membrane protein GN=LSAMP PE=1 SV=2 | Q13449\|LSAMP | 2 | 4.73 | 1 | 0.8753 | 0.81 | 0.8127 |
|  | Tropomodulin-2 GN=TMOD2 PE=1 SV=1 | Q9NZR1\|TMOD2 | 2 | 4.84 | 1 | 1.1528 | 1.24 | 0.7171 |
|  | Synaptic vesicle glycoprotein 2A GN=SV2A PE=1 SV=1 | Q7L0J3\|SV2A | 2 | 1.48 | 1 | 0.6069 | 0.86 | 1.4514 |
|  | 116 kDa U5 small nuclear ribonucleoprotein component GN=EFTUD2 PE=1 SV=1 | Q15029\|U5S1 | 2 | 1.34 | 1 | 0.8338 | 0.78 | 0.7289 |
|  | Alpha-mannosidase 2C1 GN=MAN2C1 PE=1 SV=1 | Q9NTJ4\|MA2C1 | 2 | 1.44 | 1 | 1.1476 | 0.72 | 1.0084 |
|  | Probable ATP-dependent RNA helicase DDX6 GN=DDX6 PE=1 SV=2 | P26196\|DDX6 | 2 | 2.90 | 1 | 0.8806 | 0.84 | 1.0565 |
|  | 6-phosphogluconate dehydrogenase, decarboxylating GN=PGD PE=1 SV=3 | P52209\|6PGD | 2 | 4.76 | 1 | 0.8547 | 0.84 | 0.8478 |
|  | Excitatory amino acid transporter 2 GN=SLC1A2 PE=1 SV=2 | P43004\|EAA2 | 2 | 2.61 | 1 | 0.5302 | 0.73 | 0.7872 |
|  | Calmodulin-regulated spectrin-associated protein 3 GN=CAMSAP3 PE=1 SV=2 | Q9P1Y5\|CAMP3 | 2 | 0.80 | 2 | 1.2280 | 0.93 | 1.2854 |
|  | Complement C3 GN=C3 PE=1 SV=2 | P01024\|CO3 | 2 | 0.84 | 2 | 1.2340 | 0.84 | 0.9848 |
|  | Metabotropic glutamate receptor 5 GN=GRM5 PE=1 SV=2 | P41594\|GRM5 | 2 | 0.83 | 1 | 1.2600 | 0.91 | 0.7990 |
|  | Mannosyl-oligosaccharide glucosidase GN=MOGS PE=1 SV=5 | Q13724\|MOGS | 2 | 2.15 | 1 | 0.8239 | 0.74 | 1.0054 |
|  | Dynactin subunit 1 GN=DCTN1 PE=1 SV=3 | Q14203\|DCTN1 | 2 | 2.03 | 1 | 1.2051 | 1.44 | 0.9660 |
|  | Heterogeneous nuclear ribonucleoprotein H2 GN=HNRNPH2 PE=1 SV=1 | P55795\|HNRH2 | 2 | 2.23 | 1 | 0.7728 | 0.90 | 0.7614 |
|  | Cyclin-G-associated kinase GN=GAK PE=1 SV=2 | O14976\|GAK | 2 | 1.30 | 1 | 0.6704 | 0.83 | 0.5610 |
|  | Long-chain-fatty-acid--CoA ligase 6 GN=ACSL6 PE=2 SV=4 | Q9UKU0\|ACSL6 | 2 | 1.43 | 1 | 0.6041 | 1.21 | 0.7907 |
|  | TOM1-like protein 2 GN=TOM1L2 PE=1 SV=1 | Q6ZVM7\|TM1L2 | 2 | 3.35 | 1 | 1.3707 | 1.06 | 0.7838 |
|  | Solute carrier family 12 member 2 GN=SLC12A2 PE=1 SV=1 | P55011\|S12A2 | 2 | 0.99 | 1 | 0.9477 | 0.82 | 1.1054 |
|  | UPF0317 protein C14orf159, mitochondrial GN=C14orf159 PE=2 SV=2 | Q7Z3D6\|CN159 | 2 | 1.46 | 1 | 0.8798 | 0.82 | 0.4880 |
|  | AP-3 complex subunit beta-2 GN=AP3B2 PE=1 SV=2 | Q13367\|AP3B2 | 2 | 1.20 | 1 | 0.7541 | 1.06 | 0.4510 |
|  | Peptidyl-prolyl cis-trans isomerase FKBP4 GN=FKBP4 PE=1 SV=3 | Q02790\|FKBP4 | 2 | 3.27 | 1 | 1.4642 | 1.06 | 1.5860 |
|  | Delta-1-pyrroline-5-carboxylate dehydrogenase, mitochondrial GN=ALDH4A1 PE=1 SV=3 | P30038\|AL4A1 | 2 | 3.20 | 1 | 1.0151 | 0.75 | 0.8606 |
|  | Reticulon-1 GN=RTN1 PE=1 SV=1 | Q16799\|RTN1 | 2 | 1.68 | 1 | 0.9527 | 1.19 | 1.3938 |
|  | Protein transport protein Sec23A GN=SEC23A PE=1 SV=2 | Q15436\|SC23A | 2 | 2.22 | 1 | 0.7403 | 0.89 | 1.2913 |
|  | Protein tweety homolog 1 GN=TTYH1 PE=2 SV=1 | Q9H313\|TTYH1 | 2 | 3.11 | 1 | 0.7835 | 0.86 | 1.0867 |
|  | ProSAAS GN=PCSK1N PE=1 SV=1 | Q9UHG2\|PCSK1 | 2 | 3.85 | 1 | 0.8117 | 1.49 | 0.6044 |
|  | D-3-phosphoglycerate dehydrogenase GN=PHGDH PE=1 SV=4 | O43175\|SERA | 2 | 1.50 | 1 | 1.1377 | 1.12 | 0.7165 |
|  | NADP-dependent malic enzyme, mitochondrial GN=ME3 PE=2 SV=2 | Q16798\|MAON | 2 | 3.81 | 1 | 0.7764 | 1.16 | 0.5153 |
|  | Actin-related protein 3 GN=ACTR3 PE=1 SV=3 | P61158\|ARP3 | 2 | 2.63 | 1 | 1.0801 | 1.02 | 1.0604 |
|  | Rap1 GTPase-GDP dissociation stimulator 1 GN=RAP1GDS1 PE=1 SV=3 | P52306\|GDS1 | 2 | 1.65 | 1 | 0.8735 | 0.73 | 0.6414 |
|  | Tryptophanyl-tRNA synthetase, cytoplasmic GN=WARS PE=1 SV=2 | P23381\|SYWC | 2 | 3.40 | 2 | 1.0345 | 1.05 | 1.1899 |
|  | Proliferation-associated protein 2G4 GN=PA2G4 PE=1 SV=3 | Q9UQ80\|PA2G4 | 2 | 7.87 | 1 | 1.2988 | 0.82 | 0.8554 |
|  | Vacuolar protein sorting-associated protein 45 GN=VPS45 PE=1 SV=1 | Q9NRW7\|VPS45 | 2 | 2.46 | 1 | 0.9169 | 1.25 | 1.3189 |
|  | Protein SCAI GN=SCAI PE=1 SV=2 | Q8N9R8\|SCAI | 2 | 1.98 | 1 | 0.8241 | 1.20 | 1.7870 |
|  | Protein KIAA1045 GN=KIAA1045 PE=1 SV=2 | Q9UPV7\|K1045 | 2 | 2.50 | 1 | 0.5142 | 0.71 | 0.6470 |
|  | Monoglyceride lipase GN=MGLL PE=1 SV=2 | Q99685\|MGLL | 2 | 7.92 | 1 | 0.9797 | 0.95 | 1.6856 |
|  | Serine protease HTRA1 GN=HTRA1 PE=1 SV=1 | Q92743\|HTRA1 | 2 | 2.50 | 1 | 0.7644 | 1.05 | 2.1834 |
|  | Serine/threonine-protein phosphatase 2A 56 kDa regulatory subunit delta isoform GN=PPP2R5D PE=1 SV=1 | Q14738\|2A5D | 2 | 2.33 | 1 | 0.9767 | 0.98 | 1.2842 |
|  | Growth factor receptor-bound protein 2 GN=GRB2 PE=1 SV=1 | P62993\|GRB2 | 2 | 5.53 | 1 | 0.8906 | 0.85 | 0.8916 |
|  | Adapter molecule crk GN=CRK PE=1 SV=2 | P46108\|CRK | 2 | 4.93 | 1 | 0.9114 | 0.82 | 0.8169 |
|  | Coronin-1A GN=CORO1A PE=1 SV=4 | P31146\|COR1A | 2 | 2.17 | 1 | 0.8639 | 0.80 | 0.6306 |
|  | Secretory carrier-associated membrane protein 1 GN=SCAMP1 PE=1 SV=2 | O15126\|SCAM1 | 2 | 3.25 | 1 | 0.8642 | 1.03 | 0.9047 |
|  | N-terminal EF-hand calcium-binding protein 1 GN=NECAB1 PE=1 SV=1 | Q8N987\|NECA1 | 2 | 2.85 | 1 | 1.0523 | 1.06 | 0.5911 |
|  | DnaJ homolog subfamily A member 1 GN=DNAJA1 PE=1 SV=2 | P31689\|DNJA1 | 2 | 2.02 | 1 | 0.9100 | 1.08 | 1.0768 |
|  | Solute carrier family 2, facilitated glucose transporter member 3 GN=SLC2A3 PE=1 SV=1 | P11169\|GTR3 | 2 | 2.22 | 1 | 0.7171 | 0.88 | 0.8010 |
|  | Saccharopine dehydrogenase-like oxidoreductase GN=SCCPDH PE=1 SV=1 | Q8NBX0\|SCPDL | 2 | 6.76 | 1 | 1.0128 | 1.31 | 0.4149 |
|  | Lactadherin GN=MFGE8 PE=1 SV=2 | Q08431\|MFGM | 2 | 3.88 | 1 | 1.4772 | 1.08 | 2.9630 |
|  | Programmed cell death protein 6 GN=PDCD6 PE=1 SV=1 | O75340\|PDCD6 | 2 | 5.76 | 1 | 1.1395 | 0.92 | 1.1656 |
|  | Synaptojanin-2-binding protein GN=SYNJ2BP PE=1 SV=2 | P57105\|SYJ2B | 2 | 8.28 | 1 | 0.7146 | 0.74 | 0.9896 |
|  | Small glutamine-rich tetratricopeptide repeat-containing protein alpha GN=SGTA PE=1 SV=1 | O43765\|SGTA | 2 | 4.47 | 2 | 0.7413 | 0.77 | 0.7574 |
|  | Glyoxylate reductase/hydroxypyruvate reductase GN=GRHPR PE=1 SV=1 | Q9UBQ7\|GRHPR | 2 | 7.32 | 1 | 1.6372 | 1.42 | 1.0664 |
|  | Fructosamine-3-kinase GN=FN3K PE=1 SV=1 | Q9H479\|FN3K | 2 | 5.50 | 1 | 1.5955 | 1.51 | 0.3240 |
|  | Transmembrane protein 205 GN=TMEM205 PE=1 SV=1 | Q6UW68\|TM205 | 2 | 9.00 | 1 | 0.9310 | 0.97 | 1.6985 |
|  | Galectin-related protein GN=LGALSL PE=1 SV=2 | Q3ZCW2\|LEGL | 2 | 8.14 | 1 | 0.9955 | 0.77 | 0.6338 |
|  | Single-stranded DNA-binding protein, mitochondrial GN=SSBP1 PE=1 SV=1 | Q04837\|SSBP | 2 | 10.14 | 1 | 0.6985 | 0.66 | 0.6418 |
|  | PRA1 family protein 3 GN=ARL6IP5 PE=1 SV=1 | O75915\|PRAF3 | 2 | 10.11 | 1 | 0.8916 | 0.79 | 0.9074 |
|  | DnaJ homolog subfamily A member 2 GN=DNAJA2 PE=1 SV=1 | O60884\|DNJA2 | 2 | 1.94 | 1 | 1.0058 | 1.15 | 0.8031 |
|  | Deoxyribonucleoside 5'-monophosphate N-glycosidase GN=RCL PE=1 SV=1 | O43598\|RCL | 2 | 9.77 | 1 | 1.2816 | 0.88 | 1.1188 |
|  | Redox-regulatory protein PAMM GN=C10orf58 PE=1 SV=3 | Q9BRX8\|PAMM | 2 | 6.55 | 1 | 1.0880 | 1.28 | 1.2117 |
|  | COP9 signalosome complex subunit 5 GN=COPS5 PE=1 SV=4 | Q92905\|CSN5 | 2 | 3.59 | 1 | 0.9928 | 0.97 | 0.7861 |
|  | Ermin GN=ERMN PE=2 SV=1 | Q8TAM6\|ERMIN | 2 | 4.58 | 1 | 1.6136 | 1.38 | 0.8939 |
|  | Microfibril-associated glycoprotein 4 GN=MFAP4 PE=1 SV=2 | P55083\|MFAP4 | 2 | 7.06 | 1 | 0.8034 | 1.17 | 12.8273 |
|  | NADH dehydrogenase [ubiquinone] flavoprotein 2, mitochondrial GN=NDUFV2 PE=1 SV=2 | P19404\|NDUV2 | 2 | 5.22 | 1 | 0.6374 | 0.78 | 0.5781 |
|  | Eukaryotic translation initiation factor 3 subunit F GN=EIF3F PE=1 SV=1 | O00303\|EIF3F | 2 | 4.20 | 1 | 0.8799 | 0.71 | 0.7345 |
|  | Diablo homolog, mitochondrial GN=DIABLO PE=1 SV=1 | Q9NR28\|DBLOH | 2 | 4.60 | 1 | 1.0000 | 1.16 | 1.0607 |
|  | Ubiquitin-conjugating enzyme E2 L3 GN=UBE2L3 PE=1 SV=1 | P68036\|UB2L3 | 2 | 5.84 | 1 | 1.3338 | 1.15 | 1.0369 |
|  | Neuronal membrane glycoprotein M6-a GN=GPM6A PE=1 SV=2 | P51674\|GPM6A | 2 | 5.04 | 1 | 0.6104 | 1.12 | 0.6748 |
|  | Eukaryotic translation initiation factor 1A, X-chromosomal GN=EIF1AX PE=1 SV=2 | P47813\|IF1AX | 2 | 6.94 | 1 | 1.1213 | 1.20 | 1.1823 |
|  | Cysteine and glycine-rich protein 1 GN=CSRP1 PE=1 SV=3 | P21291\|CSRP1 | 2 | 7.77 | 2 | 1.0795 | 0.93 | 3.5342 |
|  | T-cell surface glycoprotein CD8 alpha chain GN=CD8A PE=1 SV=1 | P01732\|CD8A | 2 | 3.83 | 1 | 1.9677 | 1.45 | 2.2551 |
|  | Prostamide/prostaglandin F synthase GN=C1orf93 PE=2 SV=1 | Q8TBF2\|PGFS | 2 | 9.60 | 1 | 1.2938 | 1.20 | 0.8281 |
|  | SH3 domain-binding glutamic acid-rich-like protein 2 GN=SH3BGRL2 PE=1 SV=2 | Q9UJC5\|SH3L2 | 2 | 13.08 | 1 | 1.1613 | 1.10 | 1.2667 |
|  | Cytochrome c oxidase subunit 7C, mitochondrial GN=COX7C PE=1 SV=1 | P15954\|COX7C | 2 | 14.29 | 1 | 1.1074 | 2.08 | 1.0915 |
|  | Cytochrome c1, heme protein, mitochondrial GN=CYC1 PE=1 SV=3 | P08574\|CY1 | 1.92 | 3.08 | 1 | 1.1127 | 1.12 | 1.1920 |
|  | Fatty acid-binding protein, epidermal GN=FABP5 PE=1 SV=3 | Q01469\|FABP5 | 1.89 | 6.67 | 1 | 0.9470 | 0.80 | 1.2598 |
|  | Drebrin GN=DBN1 PE=1 SV=4 | Q16643\|DREB | 1.85 | 4.31 | 1 | 0.6295 | 0.53 | 0.5427 |
|  | Kinesin light chain 1 GN=KLC1 PE=1 SV=2 | Q07866\|KLC1 | 1.84 | 2.44 | 1 | 0.8437 | 0.76 | 1.0088 |
|  | Adenylyl cyclase-associated protein 1 GN=CAP1 PE=1 SV=5 | Q01518\|CAP1 | 1.83 | 4.00 | 1 | 1.1368 | 0.60 | 0.8469 |
|  | Serine/threonine-protein phosphatase 5 GN=PPP5C PE=1 SV=1 | P53041\|PPP5 | 1.83 | 2.20 | 1 | 1.2062 | 0.95 | 0.8743 |
|  | 60S ribosomal protein L24 GN=RPL24 PE=1 SV=1 | P83731\|RL24 | 1.82 | 8.28 | 1 | 0.6536 | 0.97 | 0.7397 |
|  | Tumor protein D52 GN=TPD52 PE=1 SV=2 | P55327\|TPD52 | 1.82 | 6.25 | 1 | 1.0459 | 1.04 | 0.9390 |
|  | Ras-related C3 botulinum toxin substrate 1 GN=RAC1 PE=1 SV=1 | P63000\|RAC1 | 1.81 | 11.98 | 2 | 1.0964 | 1.11 | 0.8469 |
|  | Histidine triad nucleotide-binding protein 1 GN=HINT1 PE=1 SV=2 | P49773\|HINT1 | 1.81 | 24.60 | 2 | 0.9212 | 0.86 | 0.7606 |
|  | Eukaryotic translation initiation factor 5A-1 GN=EIF5A PE=1 SV=2 | P63241\|IF5A1 | 1.8 | 7.79 | 1 | 1.1735 | 1.13 | 0.8397 |
|  | 3-ketoacyl-CoA thiolase, mitochondrial GN=ACAA2 PE=1 SV=2 | P42765\|THIM | 1.8 | 2.52 | 1 | 1.0448 | 1.10 | 1.3052 |
|  | Phosphatidylinositol-5-phosphate 4-kinase type-2 gamma GN=PIP4K2C PE=1 SV=3 | Q8TBX8\|PI42C | 1.8 | 3.56 | 1 | 0.7591 | 0.79 | 0.6932 |
|  | Mimecan GN=OGN PE=1 SV=1 | P20774\|MIME | 1.79 | 11.74 | 2 | 0.7269 | 1.06 | 2.0506 |
|  | Fibrinogen gamma chain GN=FGG PE=1 SV=3 | P02679\|FIBG | 1.78 | 3.31 | 1 | 1.1494 | 0.91 | 1.6883 |
|  | CD44 antigen GN=CD44 PE=1 SV=3 | P16070\|CD44 | 1.74 | 1.62 | 1 | 1.1636 | 1.12 | 1.7767 |
|  | Receptor-type tyrosine-protein phosphatase zeta GN=PTPRZ1 PE=1 SV=4 | P23471\|PTPRZ | 1.73 | 0.48 | 1 | 1.0781 | 0.91 | 0.9061 |
|  | 4F2 cell-surface antigen heavy chain GN=SLC3A2 PE=1 SV=3 | P08195\|4F2 | 1.72 | 4.92 | 2 | 0.7503 | 0.97 | 0.7170 |
|  | High mobility group protein B1 GN=HMGB1 PE=1 SV=3 | P09429\|HMGB1 | 1.72 | 6.98 | 1 | 1.4077 | 1.39 | 1.8645 |
|  | Haloacid dehalogenase-like hydrolase domain-containing protein 2 GN=HDHD2 PE=1 SV=1 | Q9H0R4\|HDHD2 | 1.71 | 5.02 | 1 | 1.2042 | 1.16 | 0.9922 |
|  | Aflatoxin B1 aldehyde reductase member 2 GN=AKR7A2 PE=1 SV=3 | O43488\|ARK72 | 1.71 | 4.18 | 1 | 1.1035 | 1.06 | 1.0506 |
|  | Cytoplasmic aconitate hydratase GN=ACO1 PE=1 SV=3 | P21399\|ACOC | 1.69 | 2.25 | 1 | 0.8110 | 1.32 | 0.9037 |
|  | Nuclease EXOG, mitochondrial GN=EXOG PE=1 SV=2 | Q9Y2C4\|EXOG | 1.68 | 3.26 | 1 | 1.4324 | 1.48 | 1.2570 |
|  | X-ray repair cross-complementing protein 5 GN=XRCC5 PE=1 SV=3 | P13010\|XRCC5 | 1.68 | 1.50 | 1 | 0.9306 | 1.14 | 1.6741 |
|  | NAD-dependent deacetylase sirtuin-2 GN=SIRT2 PE=1 SV=2 | Q8IXJ6\|SIRT2 | 1.64 | 4.11 | 1 | 3.1247 | 2.34 | 1.1527 |
|  | Lambda-crystallin homolog GN=CRYL1 PE=1 SV=3 | Q9Y2S2\|CRYL1 | 1.63 | 4.39 | 1 | 1.1164 | 1.00 | 1.3325 |
|  | ES1 protein homolog, mitochondrial GN=C21orf33 PE=1 SV=3 | P30042\|ES1 | 1.63 | 17.54 | 3 | 0.9811 | 1.12 | 1.5261 |
|  | Calcium/calmodulin-dependent 3',5'-cyclic nucleotide phosphodiesterase 1C GN=PDE1C PE=1 SV=1 | Q14123\|PDE1C | 1.62 | 1.98 | 1 | 0.8427 | 0.96 | 1.1442 |
|  | NADH dehydrogenase [ubiquinone] 1 beta subcomplex subunit 9 GN=NDUFB9 PE=1 SV=3 | Q9Y6M9\|NDUB9 | 1.62 | 10.61 | 1 | 0.7212 | 0.84 | 0.9951 |
|  | Prenylcysteine oxidase 1 GN=PCYOX1 PE=1 SV=3 | Q9UHG3\|PCYOX | 1.6 | 3.56 | 1 | 0.8801 | 1.03 | 1.0470 |
|  | Toll-interacting protein GN=TOLLIP PE=1 SV=1 | Q9H0E2\|TOLIP | 1.6 | 4.74 | 1 | 1.1200 | 1.39 | 1.8243 |
|  | Myelin proteolipid protein GN=PLP1 PE=1 SV=2 | P60201\|MYPR | 1.6 | 2.53 | 1 | 1.2644 | 1.85 | 1.0114 |
|  | Ras-related protein Rab-35 GN=RAB35 PE=1 SV=1 | Q15286\|RAB35 | 1.57 | 12.94 | 2 | 0.8099 | 0.84 | 0.9633 |
|  | Protein S100-A13 GN=S100A13 PE=1 SV=1 | Q99584\|S10AD | 1.56 | 12.24 | 1 | 1.0559 | 1.32 | 1.4128 |
|  | Endonuclease domain-containing 1 protein GN=ENDOD1 PE=1 SV=2 | O94919\|ENDD1 | 1.55 | 2.00 | 1 | 1.1590 | 0.97 | 1.0695 |
|  | Ganglioside-induced differentiation-associated protein 1 GN=GDAP1 PE=1 SV=3 | Q8TB36\|GDAP1 | 1.53 | 2.24 | 1 | 0.6394 | 0.84 | 0.8653 |
|  | Probable phospholipid-transporting ATPase IA GN=ATP8A1 PE=1 SV=1 | Q9Y2Q0\|AT8A1 | 1.53 | 1.46 | 1 | 1.2810 | 1.10 | 1.0616 |
|  | 26S protease regulatory subunit 10B GN=PSMC6 PE=1 SV=1 | P62333\|PRS10 | 1.52 | 3.60 | 1 | 0.5988 | 0.81 | 0.7792 |
|  | G-protein coupled receptor family C group 5 member B GN=GPRC5B PE=2 SV=2 | Q9NZH0\|GPC5B | 1.52 | 2.98 | 1 | 1.0722 | 1.19 | 1.4118 |
|  | V-type proton ATPase subunit H GN=ATP6V1H PE=1 SV=1 | Q9UI12\|VATH | 1.5 | 4.97 | 2 | 0.9121 | 1.01 | 0.8695 |
|  | Catalase GN=CAT PE=1 SV=3 | P04040\|CATA | 1.48 | 3.04 | 1 | 1.2452 | 1.18 | 2.1987 |
|  | DNA fragmentation factor subunit alpha GN=DFFA PE=1 SV=1 | O00273\|DFFA | 1.47 | 5.14 | 1 | 0.8763 | 1.01 | 0.9516 |
|  | Galectin-3-binding protein GN=LGALS3BP PE=1 SV=1 | Q08380\|LG3BP | 1.46 | 2.91 | 1 | 1.1795 | 1.21 | 1.7703 |
|  | Heat shock 70 kDa protein 4 GN=HSPA4 PE=1 SV=4 | P34932\|HSP74 | 1.42 | 3.81 | 2 | 1.0271 | 0.92 | 0.7955 |
|  | 3-hydroxyisobutyrate dehydrogenase, mitochondrial GN=HIBADH PE=1 SV=2 | P31937\|3HIDH | 1.42 | 4.17 | 1 | 1.6220 | 1.13 | 0.7466 |
|  | Heterogeneous nuclear ribonucleoprotein H3 GN=HNRNPH3 PE=1 SV=2 | P31942\|HNRH3 | 1.42 | 2.89 | 1 | 0.5290 | 0.62 | 0.6344 |
|  | Prefoldin subunit 1 GN=PFDN1 PE=1 SV=2 | O60925\|PFD1 | 1.41 | 8.20 | 1 | 0.7942 | 1.00 | 0.8211 |
|  | Serine/threonine-protein phosphatase 2A 55 kDa regulatory subunit B delta isoform GN=PPP2R2D PE=1 SV=1 | Q66LE6\|2ABD | 1.4 | 2.43 | 1 | 0.9614 | 0.93 | 1.0080 |
|  | EF-hand domain-containing protein D2 GN=EFHD2 PE=1 SV=1 | Q96C19\|EFHD2 | 1.4 | 2.92 | 1 | 0.7520 | 0.66 | 0.6662 |
|  | ATPase ASNA1 GN=ASNA1 PE=1 SV=2 | O43681\|ASNA | 1.4 | 2.59 | 1 | 0.9097 | 1.02 | 1.1062 |
|  | Matrin-3 GN=MATR3 PE=1 SV=2 | P43243\|MATR3 | 1.38 | 1.06 | 1 | 0.9704 | 1.10 | 1.3888 |
|  | Apolipoprotein A-I GN=APOA1 PE=1 SV=1 | P02647\|APOA1 | 1.36 | 4.12 | 1 | 1.6094 | 1.16 | 1.3694 |
|  | Serum amyloid P-component GN=APCS PE=1 SV=2 | P02743\|SAMP | 1.35 | 5.38 | 1 | 1.8982 | 1.36 | 5.1449 |
|  | Outer dense fiber protein 2 GN=ODF2 PE=1 SV=1 | Q5BJF6\|ODFP2 | 1.34 | 1.69 | 1 | 0.9890 | 1.02 | 1.4926 |
|  | Phosphoglucomutase-1 GN=PGM1 PE=1 SV=3 | P36871\|PGM1 | 1.33 | 1.42 | 1 | 1.4982 | 1.21 | 1.3829 |
|  | Sarcoplasmic/endoplasmic reticulum calcium ATPase 2 GN=ATP2A2 PE=1 SV=1 | P16615\|AT2A2 | 1.31 | 2.49 | 1 | 0.7116 | 0.99 | 0.6534 |
|  | ATP synthase subunit epsilon-like protein, mitochondrial GN=ATP5EP2 PE=1 SV=1 | Q5VTU8\|AT5EL | 1.3 | 15.69 | 1 | 1.1074 | 1.14 | 0.9930 |
|  | Complement C4-B GN=C4B PE=1 SV=1 | P0C0L5\|CO4B | 1.29 | 0.52 | 1 | 1.6076 | 1.27 | 4.5000 |
|  | Trypsin-2 GN=PRSS2 PE=1 SV=1 | P07478\|TRY2 | 1.28 | 12.15 | 4 | 1.1607 | 1.02 | 1.2385 |
|  | Synaptojanin-1 GN=SYNJ1 PE=1 SV=2 | O43426\|SYNJ1 | 1.27 | 1.59 | 2 | 0.8550 | 0.87 | 0.7572 |
|  | Ig mu heavy chain disease protein PE=1 SV=1 | P04220\|MUCB | 1.27 | 3.84 | 1 | 1.5196 | 1.17 | 3.3716 |
|  | 4-trimethylaminobutyraldehyde dehydrogenase GN=ALDH9A1 PE=1 SV=3 | P49189\|AL9A1 | 1.26 | 3.04 | 1 | 1.1557 | 1.02 | 1.5745 |
|  | Calreticulin GN=CALR PE=1 SV=1 | P27797\|CALR | 1.25 | 6.00 | 2 | 1.1193 | 1.01 | 1.0732 |
|  | UTP--glucose-1-phosphate uridylyltransferase GN=UGP2 PE=1 SV=5 | Q16851\|UGPA | 1.25 | 2.36 | 1 | 1.2961 | 1.11 | 1.6563 |
|  | Protein disulfide-isomerase A3 GN=PDIA3 PE=1 SV=4 | P30101\|PDIA3 | 1.23 | 1.98 | 1 | 1.1124 | 1.05 | 1.1456 |
|  | T-complex protein 1 subunit eta GN=CCT7 PE=1 SV=2 | Q99832\|TCPH | 1.22 | 1.84 | 1 | 0.9029 | 1.00 | 0.9961 |
|  | Ras-related protein Rab-10 GN=RAB10 PE=1 SV=1 | P61026\|RAB10 | 1.21 | 11.00 | 2 | 0.8848 | 0.81 | 0.9588 |
|  | Neuroplastin GN=NPTN PE=1 SV=2 | Q9Y639\|NPTN | 1.21 | 2.51 | 1 | 1.0540 | 0.94 | 0.8781 |
|  | Guanine nucleotide-binding protein G(s) subunit alpha isoforms XLas GN=GNAS PE=1 SV=2 | Q5JWF2\|GNAS1 | 1.19 | 3.57 | 3 | 1.1640 | 1.14 | 1.3475 |
|  | Mammalian ependymin-related protein 1 GN=EPDR1 PE=1 SV=2 | Q9UM22\|EPDR1 | 1.19 | 4.46 | 1 | 1.2540 | 1.33 | 0.9970 |
|  | Dihydropyrimidinase-related protein 5 GN=DPYSL5 PE=1 SV=1 | Q9BPU6\|DPYL5 | 1.18 | 2.48 | 2 | 0.7071 | 0.70 | 0.7153 |
|  | Adenylate kinase isoenzyme 5 GN=AK5 PE=1 SV=2 | Q9Y6K8\|KAD5 | 1.17 | 3.74 | 2 | 0.8461 | 0.95 | 1.1097 |
|  | Adenylate kinase isoenzyme 4, mitochondrial GN=AK4 PE=1 SV=1 | P27144\|KAD4 | 1.17 | 4.93 | 1 | 0.9359 | 0.94 | 1.2252 |
|  | Septin-5 GN=SEPT5 PE=1 SV=1 | Q99719\|SEPT5 | 1.16 | 2.44 | 1 | 1.0004 | 0.95 | 0.8591 |
|  | Histidine triad nucleotide-binding protein 2, mitochondrial GN=HINT2 PE=1 SV=1 | Q9BX68\|HINT2 | 1.16 | 10.43 | 1 | 0.8538 | 0.99 | 0.8269 |
|  | EH domain-containing protein 2 GN=EHD2 PE=1 SV=2 | Q9NZN4\|EHD2 | 1.13 | 2.21 | 1 | 0.8998 | 0.95 | 1.5623 |
|  | Putative olfactory receptor 56B2 GN=OR56B2P PE=5 SV=1 | Q8NGI1\|O56B2 | 1.12 | 2.17 | 1 | 1.3016 | 1.03 | 1.0169 |
|  | Nucleophosmin GN=NPM1 PE=1 SV=2 | P06748\|NPM | 1.12 | 7.14 | 1 | 1.1908 | 1.09 | 0.9680 |
|  | Secernin-1 GN=SCRN1 PE=1 SV=2 | Q12765\|SCRN1 | 1.1 | 1.93 | 1 | 1.0786 | 0.97 | 0.9206 |
|  | Ubiquitin-conjugating enzyme E2 N GN=UBE2N PE=1 SV=1 | P61088\|UBE2N | 1.1 | 13.16 | 1 | 1.3619 | 1.06 | 0.8514 |
|  | Electron transfer flavoprotein subunit beta GN=ETFB PE=1 SV=3 | P38117\|ETFB | 1.1 | 4.71 | 1 | 1.1134 | 1.19 | 1.0424 |
|  | GTPase HRas GN=HRAS PE=1 SV=1 | P01112\|RASH | 1.1 | 4.76 | 1 | 0.9850 | 0.88 | 1.0275 |
|  | 40S ribosomal protein S15a GN=RPS15A PE=1 SV=2 | P62244\|RS15A | 1.08 | 6.92 | 1 | 0.5182 | 0.72 | 0.8513 |
|  | Tubulointerstitial nephritis antigen-like GN=TINAGL1 PE=1 SV=1 | Q9GZM7\|TINAL | 1.07 | 1.93 | 1 | 0.9794 | 1.08 | 4.7219 |
|  | Hydroxyacyl-coenzyme A dehydrogenase, mitochondrial GN=HADH PE=1 SV=3 | Q16836\|HCDH | 1.07 | 3.50 | 1 | 0.9064 | 0.81 | 0.5661 |
|  | Heterogeneous nuclear ribonucleoproteins C1/C2 GN=HNRNPC PE=1 SV=4 | P07910\|HNRPC | 1.07 | 3.92 | 1 | 0.9681 | 1.06 | 1.4590 |
|  | Reticulon-4 GN=RTN4 PE=1 SV=2 | Q9NQC3\|RTN4 | 1.06 | 2.01 | 2 | 3.0830 | 1.53 | 1.9294 |
|  | Argininosuccinate synthase GN=ASS1 PE=1 SV=2 | P00966\|ASSY | 1.06 | 1.94 | 1 | 1.3594 | 1.60 | 5.0363 |
|  | Calcineurin subunit B type 1 GN=PPP3R1 PE=1 SV=2 | P63098\|CANB1 | 1.06 | 16.47 | 3 | 1.0129 | 1.19 | 0.5765 |
|  | Peptidyl-prolyl cis-trans isomerase B GN=PPIB PE=1 SV=2 | P23284\|PPIB | 1.04 | 6.48 | 1 | 1.1963 | 1.06 | 1.6346 |
|  | 3-hydroxyisobutyryl-CoA hydrolase, mitochondrial GN=HIBCH PE=1 SV=2 | Q6NVY1\|HIBCH | 1.04 | 2.85 | 1 | 1.2650 | 0.98 | 1.0093 |
|  | Cytochrome b-c1 complex subunit 9 GN=UQCR10 PE=1 SV=3 | Q9UDW1\|QCR9 | 1.01 | 11.11 | 1 | 1.0194 | 0.83 | 0.9547 |
|  | Protein-glutamine gamma-glutamyltransferase 2 GN=TGM2 PE=1 SV=2 | P21980\|TGM2 | 1 | 1.89 | 1 | 1.0633 | 1.21 | 2.4378 |
|  | Ribosome maturation protein SBDS GN=SBDS PE=1 SV=4 | Q9Y3A5\|SBDS | 1 | 4.00 | 1 | 1.1944 | 1.07 | 1.2768 |
|  | Protein S100-A8 GN=S100A8 PE=1 SV=1 | P05109\|S10A8 | 1 | 11.83 | 1 | 0.7806 | 0.77 | 2.0379 |
|  | Microtubule-associated protein RP/EB family member 2 GN=MAPRE2 PE=1 SV=1 | Q15555\|MARE2 | 0.99 | 3.67 | 1 | 1.1594 | 1.05 | 1.3990 |
|  | AMME syndrome candidate gene 1 protein GN=AMMECR1 PE=2 SV=1 | Q9Y4X0\|AMER1 | 0.97 | 3.60 | 1 | 0.8545 | 0.98 | 1.0836 |
|  | Probable phospholipid-transporting ATPase IIB GN=ATP9B PE=2 SV=4 | O43861\|ATP9B | 0.95 | 0.61 | 1 | 1.3685 | 1.05 | 0.4282 |
|  | V-type proton ATPase subunit d 1 GN=ATP6V0D1 PE=1 SV=1 | P61421\|VA0D1 | 0.95 | 2.28 | 1 | 0.7181 | 0.91 | 0.7728 |
|  | Sodium/calcium exchanger 2 GN=SLC8A2 PE=2 SV=2 | Q9UPR5\|NAC2 | 0.94 | 2.71 | 1 | 0.8562 | 0.70 | 0.9077 |
|  | Succinyl-CoA ligase [ADP-forming] subunit beta, mitochondrial GN=SUCLA2 PE=1 SV=3 | Q9P2R7\|SUCB1 | 0.92 | 1.94 | 1 | 0.9328 | 1.09 | 0.7680 |
|  | Atlastin-3 GN=ATL3 PE=1 SV=1 | Q6DD88\|ATLA3 | 0.9 | 1.66 | 1 | 0.9501 | 0.98 | 2.8388 |
|  | Serine/threonine-protein kinase DCLK1 GN=DCLK1 PE=1 SV=2 | O15075\|DCLK1 | 0.89 | 1.35 | 1 | 0.6313 | 0.67 | 0.7985 |
|  | NADH-cytochrome b5 reductase 3 GN=CYB5R3 PE=1 SV=3 | P00387\|NB5R3 | 0.87 | 3.99 | 1 | 1.2017 | 1.08 | 1.0034 |
|  | Destrin GN=DSTN PE=1 SV=3 | P60981\|DEST | 0.86 | 10.30 | 1 | 1.1180 | 1.09 | 1.5559 |
|  | Ig kappa chain C region GN=IGKC PE=1 SV=1 | P01834\|IGKC | 0.86 | 16.98 | 1 | 1.9560 | 1.43 | 1.4038 |
|  | Myosin-14 GN=MYH14 PE=1 SV=2 | Q7Z406\|MYH14 | 0.84 | 1.15 | 2 | 1.0848 | 0.95 | 1.0190 |
|  | von Willebrand factor A domain-containing protein 1 GN=VWA1 PE=2 SV=1 | Q6PCB0\|VWA1 | 0.84 | 4.04 | 1 | 0.7458 | 0.91 | 1.7892 |
|  | Apolipoprotein E GN=APOE PE=1 SV=1 | P02649\|APOE | 0.83 | 2.84 | 1 | 0.9138 | 1.07 | 1.4636 |
|  | Sodium-driven chloride bicarbonate exchanger GN=SLC4A10 PE=1 SV=1 | Q6U841\|S4A10 | 0.83 | 0.98 | 1 | 0.2775 | 0.70 | 0.4265 |
|  | Core histone macro-H2A.1 GN=H2AFY PE=1 SV=4 | O75367\|H2AY | 0.82 | 10.22 | 3 | 1.1234 | 1.26 | 1.3190 |
|  | Acyl-CoA-binding protein GN=DBI PE=1 SV=2 | P07108\|ACBP | 0.82 | 18.39 | 1 | 1.4745 | 1.46 | 1.5432 |
|  | Putative myosin light chain kinase 3 GN=MYLK3 PE=2 SV=3 | Q32MK0\|MYLK3 | 0.81 | 0.98 | 1 | 0.7758 | 0.83 | 1.0304 |
|  | Acyl-CoA synthetase short-chain family member 3, mitochondrial GN=ACSS3 PE=2 SV=1 | Q9H6R3\|ACSS3 | 0.79 | 1.60 | 1 | 1.1745 | 1.11 | 2.0323 |
|  | Brevican core protein GN=BCAN PE=1 SV=2 | Q96GW7\|PGCB | 0.79 | 0.77 | 1 | 0.9441 | 0.92 | 0.7163 |
|  | SH3 domain-binding glutamic acid-rich-like protein 3 GN=SH3BGRL3 PE=1 SV=1 | Q9H299\|SH3L3 | 0.79 | 10.75 | 1 | 1.1735 | 0.63 | 0.8736 |
|  | Ras-related protein Ral-A GN=RALA PE=1 SV=1 | P11233\|RALA | 0.78 | 5.82 | 1 | 0.8798 | 0.87 | 0.5997 |
|  | Fibroblast growth factor receptor 4 GN=FGFR4 PE=1 SV=2 | P22455\|FGFR4 | 0.76 | 1.00 | 1 | 0.7700 | 0.82 | 0.9009 |
|  | NudC domain-containing protein 3 GN=NUDCD3 PE=1 SV=3 | Q8IVD9\|NUDC3 | 0.76 | 2.22 | 1 | 1.1971 | 0.78 | 0.6323 |
|  | Phosphorylase b kinase regulatory subunit alpha, liver isoform GN=PHKA2 PE=1 SV=1 | P46019\|KPB2 | 0.75 | 0.57 | 1 | 0.9315 | 1.28 | 1.2676 |
|  | G-protein coupled receptor 64 GN=GPR64 PE=1 SV=2 | Q8IZP9\|GPR64 | 0.75 | 1.48 | 1 | 0.7399 | 0.88 | 1.1298 |
|  | Protein CutA GN=CUTA PE=1 SV=2 | O60888\|CUTA | 0.73 | 7.82 | 1 | 0.9256 | 0.74 | 0.9927 |
|  | Cytochrome b5 type B GN=CYB5B PE=1 SV=2 | O43169\|CYB5B | 0.73 | 6.16 | 1 | 0.6911 | 0.76 | 1.2104 |
|  | Ras-related protein Rab-15 GN=RAB15 PE=1 SV=1 | P59190\|RAB15 | 0.7 | 9.91 | 2 | 0.7437 | 0.68 | 0.6605 |
|  | Histone H2A.V GN=H2AFV PE=1 SV=3 | Q71UI9\|H2AV | 0.69 | 35.16 | 3 | 0.9624 | 1.02 | 0.6374 |
|  | Rho GTPase-activating protein 32 GN=ARHGAP32 PE=1 SV=1 | A7KAX9\|RHG32 | 0.67 | 0.48 | 1 | 1.0012 | 1.10 | 1.0015 |
|  | Dynein light chain 1, cytoplasmic GN=DYNLL1 PE=1 SV=1 | P63167\|DYL1 | 0.66 | 7.86 | 1 | 1.1159 | 1.58 | 1.0195 |
|  | Heterogeneous nuclear ribonucleoprotein D-like GN=HNRPDL PE=1 SV=3 | O14979\|HNRDL | 0.66 | 1.91 | 1 | 1.1577 | 1.43 | 1.3954 |
|  | Protein phosphatase 1 regulatory subunit 12A GN=PPP1R12A PE=1 SV=1 | O14974\|MYPT1 | 0.66 | 0.97 | 1 | 1.1943 | 1.11 | 1.3229 |
|  | Septin-8 GN=SEPT8 PE=1 SV=4 | Q92599\|SEPT8 | 0.65 | 3.73 | 2 | 1.3145 | 0.76 | 0.7589 |
|  | Septin-11 GN=SEPT11 PE=1 SV=3 | Q9NVA2\|SEP11 | 0.34 | 7.46 | 3 | 0.9920 | 0.76 | 1.1499 |
|  | Acid ceramidase GN=ASAH1 PE=1 SV=5 | Q13510\|ASAH1 | 0.65 | 3.04 | 1 | 1.0140 | 0.93 | 1.0647 |
|  | Cytochrome b-c1 complex subunit Rieske, mitochondrial GN=UQCRFS1 PE=1 SV=2 | P47985\|UCRI | 0.65 | 12.41 | 2 | 0.9299 | 1.11 | 0.9131 |
|  | Tubulin polymerization-promoting protein family member 3 GN=TPPP3 PE=1 SV=1 | Q9BW30\|TPPP3 | 0.65 | 7.95 | 1 | 0.9360 | 0.90 | 1.1217 |
|  | L-asparaginase GN=ASRGL1 PE=1 SV=2 | Q7L266\|ASGL1 | 0.65 | 3.25 | 1 | 0.9686 | 0.84 | 0.5767 |
|  | Carnitine O-palmitoyltransferase 1, liver isoform GN=CPT1A PE=1 SV=2 | P50416\|CPT1A | 0.64 | 1.55 | 1 | 0.3244 | 0.71 | 0.8365 |
|  | Electrogenic sodium bicarbonate cotransporter 1 GN=SLC4A4 PE=1 SV=1 | Q9Y6R1\|S4A4 | 0.63 | 2.22 | 1 | 0.5304 | 0.70 | 0.6748 |
|  | Macrophage-capping protein GN=CAPG PE=1 SV=2 | P40121\|CAPG | 0.63 | 2.01 | 1 | 1.0871 | 1.09 | 2.2206 |
|  | Coronin-1C GN=CORO1C PE=1 SV=1 | Q9ULV4\|COR1C | 0.62 | 7.81 | 2 | 0.8782 | 0.91 | 1.1469 |
|  | Cytosol aminopeptidase GN=LAP3 PE=1 SV=3 | P28838\|AMPL | 0.62 | 3.66 | 1 | 1.2954 | 1.06 | 0.4797 |
|  | Collagen alpha-1(XII) chain GN=COL12A1 PE=1 SV=2 | Q99715\|COCA1 | 0.61 | 0.29 | 1 | 0.9744 | 0.77 | 1.5633 |
|  | Melanotransferrin GN=MFI2 PE=1 SV=2 | P08582\|TRFM | 0.61 | 0.95 | 1 | 5.2228 | 2.29 | 2.4472 |
|  | 26S proteasome non-ATPase regulatory subunit 13 GN=PSMD13 PE=1 SV=2 | Q9UNM6\|PSD13 | 0.61 | 7.98 | 1 | 1.4097 | 1.20 | 0.7709 |
|  | F-box/LRR-repeat protein 17 GN=FBXL17 PE=2 SV=3 | Q9UF56\|FXL17 | 0.6 | 1.43 | 1 | 1.3416 | 1.10 | 0.9456 |
|  | Serine/arginine-rich splicing factor 8 GN=SRSF8 PE=1 SV=1 | Q9BRL6\|SRSF8 | 0.6 | 2.84 | 1 | 0.9986 | 1.04 | 1.2200 |
|  | WD repeat-containing protein 47 GN=WDR47 PE=1 SV=1 | O94967\|WDR47 | 0.58 | 0.76 | 1 | 1.1763 | 1.16 | 2.0780 |
|  | CB1 cannabinoid receptor-interacting protein 1 GN=CNRIP1 PE=1 SV=1 | Q96F85\|CNRP1 | 0.58 | 14.02 | 1 | 0.9343 | 1.00 | 0.7464 |
|  | 26S proteasome non-ATPase regulatory subunit 3 GN=PSMD3 PE=1 SV=2 | O43242\|PSMD3 | 0.55 | 2.43 | 1 | 0.9847 | 1.14 | 1.3065 |
|  | Alcohol dehydrogenase [NADP+] GN=AKR1A1 PE=1 SV=3 | P14550\|AK1A1 | 0.55 | 3.38 | 1 | 1.3480 | 1.17 | 1.3419 |
|  | Palmitoyl-protein thioesterase 1 GN=PPT1 PE=1 SV=1 | P50897\|PPT1 | 0.55 | 5.88 | 2 | 0.9820 | 1.12 | 0.8554 |
|  | Bis(5'-nucleosyl)-tetraphosphatase [asymmetrical] GN=NUDT2 PE=1 SV=3 | P50583\|AP4A | 0.55 | 5.44 | 1 | 0.7386 | 0.68 | 0.6722 |
|  | Lysine-specific demethylase 4D-like GN=KDM4DL PE=1 SV=1 | B2RXH2\|KD4DL | 0.55 | 2.57 | 1 | 1.2993 | 1.25 | 1.1759 |
|  | Thioredoxin domain-containing protein 17 GN=TXNDC17 PE=1 SV=1 | Q9BRA2\|TXD17 | 0.54 | 8.13 | 1 | 1.1565 | 0.90 | 1.1958 |
|  | DENN domain-containing protein 1A GN=DENND1A PE=1 SV=2 | Q8TEH3\|DEN1A | 0.53 | 0.89 | 1 | 0.8702 | 0.80 | 0.9527 |
|  | 6-phosphogluconolactonase GN=PGLS PE=1 SV=2 | O95336\|6PGL | 0.53 | 5.81 | 1 | 1.2558 | 0.74 | 0.9939 |
|  | Calnexin GN=CANX PE=1 SV=2 | P27824\|CALX | 0.52 | 1.69 | 1 | 0.9375 | 1.56 | 2.2080 |
|  | Epsin-1 GN=EPN1 PE=1 SV=2 | Q9Y6I3\|EPN1 | 0.52 | 1.74 | 1 | 0.8776 | 0.76 | 0.7238 |
|  | Putative adenosylhomocysteinase 3 GN=AHCYL2 PE=1 SV=1 | Q96HN2\|SAHH3 | 0.5 | 1.96 | 1 | 0.5996 | 0.56 | 0.6609 |
|  | Microtubule-associated proteins 1A/1B light chain 3B GN=MAP1LC3B PE=1 SV=3 | Q9GZQ8\|MLP3B | 0.5 | 16.80 | 2 | 1.6908 | 1.45 | 0.5586 |
|  | Alpha-adducin GN=ADD1 PE=1 SV=2 | P35611\|ADDA | 0.48 | 2.44 | 3 | 0.9856 | 1.10 | 0.8511 |
|  | Tubulin beta-6 chain GN=TUBB6 PE=1 SV=1 | Q9BUF5\|TBB6 | 0.47 | 37.44 | 47 | 0.9592 | 0.89 | 0.4240 |
|  | Probable ATP-dependent RNA helicase DDX17 GN=DDX17 PE=1 SV=1 | Q92841\|DDX17 | 0.44 | 3.08 | 1 | 1.0247 | 1.00 | 1.1010 |
|  | Ras-related protein Rab-5C GN=RAB5C PE=1 SV=2 | P51148\|RAB5C | 0.4 | 9.72 | 2 | 0.6871 | 0.85 | 0.8853 |
|  | Acetyl-CoA acetyltransferase, cytosolic GN=ACAT2 PE=1 SV=2 | Q9BWD1\|THIC | 0.4 | 2.77 | 1 | 1.1265 | 1.08 | 0.8718 |
|  | Glutathione S-transferase Mu 3 GN=GSTM3 PE=1 SV=3 | P21266\|GSTM3 | 0.39 | 11.11 | 2 | 1.3023 | 0.88 | 1.1684 |
|  | Coactosin-like protein GN=COTL1 PE=1 SV=3 | Q14019\|COTL1 | 0.38 | 11.27 | 1 | 0.8727 | 0.77 | 1.0352 |
|  | Septin-11 GN=SEPT11 PE=1 SV=3 | Q9NVA2\|SEP11 | 0.34 | 7.46 | 3 | 0.9920 | 0.76 | 1.1499 |
|  | Serine/threonine-protein kinase PAK 3 GN=PAK3 PE=1 SV=2 | O75914\|PAK3 | 0.3 | 2.15 | 1 | 0.8108 | 1.08 | 0.8190 |
|  | SPARC-like protein 1 GN=SPARCL1 PE=1 SV=2 | Q14515\|SPRL1 | 0.3 | 1.96 | 1 | 0.9074 | 1.07 | 1.0527 |
|  | Guanine nucleotide-binding protein subunit alpha-13 GN=GNA13 PE=1 SV=2 | Q14344\|GNA13 | 0.28 | 7.69 | 3 | 1.1867 | 1.13 | 1.1422 |
|  | Septin-9 GN=SEPT9 PE=1 SV=2 | Q9UHD8\|SEPT9 | 0.27 | 2.05 | 1 | 1.1680 | 1.02 | 1.5783 |
|  | Elongation factor 2 GN=EEF2 PE=1 SV=4 | P13639\|EF2 | 0.23 | 2.10 | 1 | 0.9834 | 1.03 | 1.0086 |
|  | Serpin B6 GN=SERPINB6 PE=1 SV=3 | P35237\|SPB6 | 0.21 | 5.32 | 1 | 1.3136 | 1.00 | 2.0421 |
|  | Prostaglandin-H2 D-isomerase GN=PTGDS PE=1 SV=1 | P41222\|PTGDS | 0.18 | 8.42 | 2 | 1.2508 | 0.99 | 1.3103 |
|  | UPF0452 protein C7orf41 GN=C7orf41 PE=2 SV=2 | Q8N3F0\|CG041 | 0.16 | 9.16 | 1 | 1.3272 | 1.07 | 0.7876 |
|  | Copine-2 GN=CPNE2 PE=1 SV=3 | Q96FN4\|CPNE2 | 0.15 | 1.64 | 1 | 0.6378 | 0.58 | 0.6041 |
|  | Endophilin-B2 GN=SH3GLB2 PE=1 SV=1 | Q9NR46\|SHLB2 | 0.14 | 3.80 | 1 | 0.9609 | 0.91 | 1.1678 |
|  | Serine/threonine-protein phosphatase 2B catalytic subunit gamma isoform GN=PPP3CC PE=1 SV=3 | P48454\|PP2BC | 0.13 | 1.95 | 1 | 0.9287 | 1.15 | 1.2072 |
|  | Calcium/calmodulin-dependent protein kinase type II subunit beta GN=CAMK2B PE=1 SV=3 | Q13554\|KCC2B | 0.09 | 10.06 | 5 | 0.6931 | 0.82 | 0.4128 |
|  | Nucleoside diphosphate kinase B GN=NME2 PE=1 SV=1 | P22392\|NDKB | 0.07 | 13.82 | 2 | 1.3478 | 1.27 | 0.9974 |
|  | Kinesin-like protein KIF7 GN=KIF7 PE=1 SV=2 | Q2M1P5\|KIF7 | 0.06 | 0.82 | 1 | 0.7107 | 0.97 | 0.9457 |
|  | Zinc finger protein 606 GN=ZNF606 PE=2 SV=1 | Q8WXB4\|ZN606 | 0.06 | 0.76 | 1 | 0.8049 | 0.71 | 0.8396 |

^a^ UniProtKB ID, UniProt Knowledgebase Identifier. ^b^ Unused, Unused Protein Score. It is calculated using only peptides from spectra that have not been used by other proteins; ^c^ % Cov. (≥95), Percentage of coverage; ^d^ Pep. Ident. (**≥**95), Number of peptides with at least 95% of confidence in their identification; ^e^ 114:113, ADB1/Normal brain ratio; ^f^ 116:113, ADB2/Normal brain ratio; ^g^ 118:113, ADB3/Normal brain ratio. GN, Gene Name. PE, Protein Existence: 1. Experimental evidence at protein level; 2. Experimental evidence at transcript level; 3. Protein inferred from homology; 4. Protein predicted; 5. Protein uncertain. SV, Sequence Variant. GN, PE and SV are described for each protein file in UniProtKB.
