# Supplementary material for: Data set of interactomes and metabolic pathways of proteins differentially expressed in brains with Alzheimer׳s disease
Source: Data Brief. 2016 May 6;7:1707–19. doi: 10.1016/j.dib.2016.04.071 (PMC4878460; doi:10.1016/j.dib.2016.04.071)
Supplement: Supplementary file 3 — Table 2. [file mmc3.docx]

**Table 2. Classification of the overexpressed and subexpressed proteins found in common in all brains with Alzheimer’s disease according to diseases and biofunctions with the IPA software (Core I).**

| **Category** | **p-value** | **N** | **Molecules** |
| --- | --- | --- | --- |
| 1. Neurological Disease | 5.89 x 10^-14^  to  2.18 x 10^-2^ | 62 | **CRYAB**, **FTL**, *CCT2*, **SERPINA3**, *MT-CO2*, **LDHB**, **CTSD**, **SOD2**, *SLC25A6*, **APCS**, *DLG4*, **GFAP**, *KIF5C*, **ALDH6A1**, *TUBB2A*, **GSN**, *CPNE5*, *RHOA*, **RTN4**, RAB11A, **GSTP1**, **FTH1**, **PRDX2**, **HSPB1**, **CA2**, *MAP2*, **PRDX1**, *GAK*, **IGHG1**, **PRDX6**, *SLC12A5*, **VCAN**, **COL1A2**, C4A/**C4B**, **MFI2**, **PREPL**, *EFTUD2*, **ANXA5**, *NEDD8*, *OPA1*, **SERPINA1**, *CAMK2B*, **S100A1**, **GSTM1**, *SLC25A4*, **DBI**, *GLS*, **IGKC**, **HNRNPDL**, *NDUFS3*, *NDUFA13*, *DNM1*, **HP**, *UBQLN2*, **APOA1**, *TUBA1A*, *NDUFS8*, *NDUFV2*, **ORM2**, *SLC1A2*, *SYN2*, **A2M** |
| 1. Psychological Disorders | 5.89 x 10^-14^  to  1.62 x 10^-2^ | 53 | **CRYAB**,**FTL**,**SERPINA3**,*MT-CO2*, **LDHB**, **CTSD**, *SLC25A6*, **SOD2**, *DLG4*, **GFAP**, **ALDH6A1**, *TUBB2A*, **GSN**, *CPNE5*, **RTN4**, RAB11A, **GSTP1**, **FTH1**, **PRDX2**, **HSPB1**, **CA2**, *MAP2*, **PRDX1**, *GAK*, **IGHG1**, **PRDX6**, *SLC12A5*, **VCAN**, C4A/**C4B**, **MFI2**, **PREPL**, *NEDD8*, *OPA1*, **SERPINA1**, *CAMK2B*, **S100A1**, **GSTM1**, *SLC25A4*, **IGKC**, *GLS*, **HNRNPDL**, *NDUFS3*, *NDUFA13*, *DNM1*, **HP**, *ANXA6*, **APOA1**, *TUBA1A*, *NDUFS8*, *NDUFV2*, *SLC1A2*, *SYN2*, **A2M** |
| 1. Skeletal and Muscular Disorders | 6.99 x 10^-11^  to  2.18 x 10^-2^ | 50 | **CA2**,**FTL**,**CRYAB**,*MAP2*,**PRDX1**,**SERPINA3**,**IGHG1**,**VCAN**,**PRDX6**,**LDHB**,C4A/**C4B**,**COL1A2**,**HMGB1**,**AKR1A1**,**SOD2**,*SLC25A6*,**PREPL**,*EFTUD2*,**SERPINA1**,**GFAP**,**ALDH6A1**,**ACTA1**,*CAMK2B*,**GSTM1**,*SLC25A4*,**IGKC**,*TUBB2A*,**GSN**,**HNRNPDL**,*NDUFS3*,*NDUFA13*,*DNM1*,*GNAI3*,**HP**,**APOA1**,*TUBA1A*,*NDUFS8*,*CPNE5*,**S100A9**,*RHOA*,**CAT**,**RTN4**,RAB11A,*SLC1A2*,**A2M**,*SYN2*,**GSTP1**,**FTH1**,**PRDX2**,**HSPB1** |
| 1. Cancer | 1.02 x 10^-9^  to  2.18 x 10^-2^ | 52 | *DBN1*,**CRYAB**,**ASS1**,*CCT2*,*MT-CO2*, **YWHAQ**, **CTSD**, **SOD2**, *SLC25A6*, **APCS**, **GFAP**, *CPT1A*, *TUBB2A*, **GSN**, *NRCAM*, *RHOA*, *RPS15A*, **GSTP1**, **FTH1**, **PRDX2**, *AKAP12*, *SLC4A4*, *DCLK1*, **VCAN**, **PRDX6**, **COL1A2**, C4A/**C4B**, **MFI2**, **PREPL**, *PHB*, **ANXA5**, *EFTUD2*, *NEDD8*, *ATP5J2*, **SERPINA1**, **S100A1**, **PSAT1**, **GSTM1**, *SLC25A4*, **IGKC**, *NDUFA13*, *CRMP1*, **HP**, *TUBA1A*, **APOA1**, *ANXA6*, *UBQLN2*, *NDUFV2*, **S100A9**, **LGALS3BP**, *PEA15*, *HAGH* |
| 1. Organismal Injury and Abnormalities | 1.02 x 10^-9^  to  2.18 x 10^-2^ | 44 | *SLC4A4*,**CA2**,**CRYAB**,**ASS1**,*CCT2*,**PRDX6**,**VCAN**,**HMGB1**,**YWHAQ**,**COL1A2**,C4A/**C4B**,**CTSD**,**MFI2**,*SLC25A6*,**SOD2**,*PHB*,**APCS**,*EFTUD2*,**ANXA5**,*NEDD8*,*ATP5J2*,**SERPINA1**,*KIF5C*,**S100A1**,**PSAT1**,**GSTM1**,*SLC25A4*,**DBI**,**IGKC**,*TUBB2A*,**GSN**,*CRMP1*,**HP**,*TUBA1A*,**APOA1**,**S100A9**,*NRCAM*,*RHOA*,**CAT**,*PEA15*,*HAGH*,**GSTP1**,**PRDX2**,**HSPB1** |
| 1. Hereditary Disorder | 1.35 x 10^-8^  to  2.18 x 10^-2^ | 59 | **CRYAB**,**FTL**,**ASS1**,**SERPINA3**,*MT-CO2*, **CD8A**, **LDHB**, **CTSD**, **SOD2**, **APCS**, *DLG4*, **GFAP**, *KIF5C*, **ACTA1**, **ALDH6A1**, *CPT1A*, *TUBB2A*, **PGM1**, **GSN**, *CPNE5*, **RTN4**, RAB11A, **HSPB1**, **FTH1**, *SLC4A4*, **CA2**, *MAP2*, **VCAN**, **PRDX6**, *SLC12A5*, C4A/**C4B**, **COL1A2**, **PREPL**, **ANXA5**, *EFTUD2*, *NEDD8*, *OPA1*, **SERPINA1**, *CAMK2B*, **S100A1**, **PSAT1**, **GSTM1**, *SLC25A4*, **IGKC**, **HNRNPDL**, *NDUFS3*, *NDUFA13*, *DNM1*, *GNAI3*, **HP**, **APOA1**, *TUBA1A*, *NDUFS8*, *NDUFV2*, **CAT**, *SLC1A2*, *SYN2*, **A2M**, *HAGH* |
| 1. Endocrine System Disorders | 3.53 x 10^-8^  to  2.15 x 10^-2^ | 23 | **CA2**,**GSTM1**,**PSAT1**,*CPT1A*,*TUBB2A*,**PRDX6**,*NDUFA13*,**VCAN**,C4A/**C4B**,**CTSD**,**HP**,**SOD2**,**APOA1**,*TUBA1A*,**PREPL**,*NRCAM*,**APCS**,**ANXA5**,**CAT**,*PEA15*,**SERPINA1**,**GSTP1**,**PRDX2** |
| 1. Free Radical Scavenging | 8.29 x 10^-8^  to  2.18 x 10^-2^ | 14 | **FTL**,**CRYAB**,**EXOG**,**PRDX1**,**GSN**,**PRDX6**,**HMGB1**,**SOD2**,**CAT**,*DLST*,**SERPINA1**,**HSPB1**,**PRDX2**,**FTH1** |
| 1. Metabolic Disease | 1.37 x 10^-7^  to  2.15 x 10^-2^ | 36 | *SLC4A4*,**CA2**,**PRDX1**,**ASS1**,*GAK*,**SERPINA3**,**IGHG1**,*MT-CO2*, **LDHB**, C4A/**C4B**, **COL1A2**, **CTSD**,**MFI2**,**SOD2**,*OPA1*,**SERPINA1**,**GFAP**,**ALDH6A1**,*CAMK2B*,**PSAT1**,*CPT1A*,*SLC25A4*,**IGKC**,**PGM1**,**GSN**,**HNRNPDL**,*NDUFS3*,**HP**,**APOA1**,*NDUFS8*,*NDUFV2*,**CAT**,*SLC1A2*,**A2M**,*HAGH*,**FTH1** |
| 1. Cell Death and Survival | 1.54 x 10^-7^  to  2.18 x 10^-2^ | 49 | *AKAP12*,**CA2**,**CRYAB**,**EXOG**,**PRDX1**,**VTN**,*CCT2*,**SERPINA3**,**IGHG1**,*NUDT2*,**CD8A**,*AP2A2*,**PRDX6**,**SYNM**,C4A/**C4B**,**HMGB1**,**YWHAQ**,**CTSD**,*SLC25A6*,**SOD2**,*PHB*,**HIST1H2BO**,**ANXA5**,*DLST*,*OPA1*,*CAMK2B*,**S100A1**,**GSTM1**,*SLC25A4*,**IGKC**,**GSN**,*NDUFA13*,*DNM1*,**HP**,*UBQLN2*,**SIRT2**,*TUBA1A*,**APOA1**,**S100A9**,*RHOA*,**LGALS3BP**,**CAT**,*PEA15*,**RTN4**,RAB11A,**GSTP1**,**FTH1**,**HSPB1**,**PRDX2** |
| 1. Small Molecule Biochemistry | 3.49 x 10^-6^  to  2.18 x 10^-2^ | 17 | **PRDX1**,**VTN**,**PRDX6**,*PGD*,**AKR1A1**,**SOD2**,**APOA1**,**ANXA5**,*RHOA*,**CAT**,*SLC1A2*,*OPA1*, **SERPINA1**,*CMPK1*,**GSTP1**,**PRDX2**,**FTH1** |
| 1. Molecular Transport | 4.9 x 10^-6^  to  2.18 x 10^-2^ | 16 | **S100A1**, **FTL**, **EXOG**, **PRDX1**, **GSN**, **CD8A**, **PRDX6**, **APOA1**, **SOD2**, **S100A9**, **CAT**, *SLC1A2*, **A2M**, **GSTP1**, **PRDX2**, **FTH1** |
| 1. Immunological Disease | 1.08 x 10^-5^  to  1.46 x 10^-2^ | 30 | **CA2**,**PRDX1**,**IGHG1**,**CD8A**,**LDHB**,**COL1A2**,C4A/**C4B**,**HMGB1**,**CTSD**,**AKR1A1**,**SOD2**, **HIST1H2BO**,**ANXA5**,**GFAP**,**ACTA1**,**IGKC**,*TUBB2A*,**PGM1**,**GSN**,*DNM1*,**HP**,*TUBA1A*,**APOA1**,**SIRT2**,**S100A9**,**LGALS3BP**,**CAT**,*SLC1A2*,**PRDX2**,**FTH1** |
| 1. Cell Morphology | 1.12 x 10^-5^  to  2.18 x 10^-2^ | 26 | *AKAP12*,**PRDX1**,**VTN**,*AP2A2*,**VCAN**,**SYNM**,**SOD2**,*SLC25A6*,*PHB*,**ANXA5**,*OPA1*,*KIF5C*, **S100A1**,*AP2M1*,**GSN**,*DNM1*,*CRMP1*,*GNAI3*,*NDUFV2*,*ATP6V1F*,**S100A9**,*RHOA*,**CAT**,**RTN4**,RAB11A,**HSPB1** |
| 1. Cellular Function and Maintenance | 1.12 x 10^-5^  to  2.18 x 10^-2^ | 28 | *MAP2*,*ATP6V1D*,**VTN**,**CD8A**,*AP2A2*,**VCAN**,**HMGB1**,**SOD2**,*SLC25A6*,*PHB*,**APCS**,*OPA1*, *KIF5C*,**GSTM1**,*AP2M1*,**GSN**,*DNM1*,*GNAI3*,**APOA1**,*ANXA6*,*ATP6V1F*,**S100A9**,*RHOA*,**LGALS3BP**,**CAT**,RAB11A,**IGHA1**,**HSPB1** |
| 1. Connective Tissue Disorders | 1.33 x 10^-5^  to  2.01 x 10^-2^ | 29 | **CA2**,**CRYAB**,**FTL**,**PRDX1**,**IGHG1**,**LDHB**,**COL1A2**,**HMGB1**,**AKR1A1**,**SOD2**,**APCS**, *EFTUD2*,**GFAP**,**ACTA1**,*CAMK2B*,**IGKC**,*TUBB2A*,**GSN**,*DNM1*,*GNAI3*,**HP**,*TUBA1A*,**APOA1**,**S100A9**,*RHOA*,*SLC1A2*,**GSTP1**,**PRDX2**,**FTH1** |
| 1. Inflammatory Disease | 1.33 x 10^-5^  to  2.01 x 10^-2^ | 31 | **FTL**,**CRYAB**,**PRDX1**,**IGHG1**,**LDHB**,C4A/**C4B**,**HMGB1**,**COL1A2**,**CTSD**,**AKR1A1**,**SOD2**, **ANXA5**,**SERPINA1**,**GFAP**,**ACTA1**,*CAMK2B*,**S100A1**,**IGKC**,*TUBB2A*,**GSN**,*DNM1*,**HP**,*TUBA1A*,**APOA1**,**S100A9**,*RHOA*,**CAT**,*SLC1A2*,**GSTP1**,**PRDX2**,**FTH1** |
| 1. Ophthalmic Disease | 4.17 x 10^-5^  to  2.15 x 10^-2^ | 10 | **CRYAB**,**FTL**,*TUBA1A*,*SLC25A4*,*TUBB2A*,*OPA1*,*SLC1A2*,**SERPINA3**,**GSN**,**VCAN** |
| 1. Cellular Movement | 4.48 x 10^-5^  to  2.18 x 10^-2^ | 27 | **CRYAB**,**VTN**,**SERPINA3**,**IGHG1**,**VCAN**,**SYNM**,**YWHAQ**,**HMGB1**,C4A/**C4B**,**CTSD**,**MFI2**, **SOD2**,*PHB*,**SERPINA1**,*AP2M1*,**GSN**,*DNM1*,*CRMP1*,*GNAI3*,**APOA1**,**S100A9**,*RHOA*,**CAT**,**RTN4**,**A2M**,**HSPB1**,**PRDX2** |
| 1. Cellular Development | 5.3 x 10^-5^  to  1.46 x 10^-2^ | 28 | *AKAP12*,**FTL**,**VTN**,**IGHG1**,**CD8A**,**VCAN**,**SYNM**,**YWHAQ**,**HMGB1**,**CTSD**,*SLC25A6*,**SOD2**, *PHB*,**SERPINA1**,**GSTM1**,**IGKC**,*TUBB2A*,*NDUFA13*,**SIRT2**,**APOA1**,*ANXA6*,*SSBP1*,*RHOA*,**CAT**,**A2M**,**HSPB1**,**FTH1**,**PRDX2** |
| 1. Cellular Growth and Proliferation | 5.3 x 10^-5^  to  1.46 x 10^-2^ | 37 | *AKAP12*,*DBN1*,**FTL**,**VTN**,*CCT2*,*GAK*,**IGHG1**,**CD8A**,**VCAN**,**SYNM**,**HMGB1**,**YWHAQ**,**CTSD**,**SOD2**,*SLC25A6*,*PHB*,*DLST*,**SERPINA1**,**GSTM1**,*SLC25A4*,**IGKC**,*TUBB2A*,**GSN**,*NDUFS3*,*NDUFA13*,**APOA1**,*ANXA6*,*SSBP1*,**SIRT2**,*RHOA*,**CAT**,**RTN4**,*RPS15A*,**A2M**,**GSTP1**,**FTH1**,**PRDX2** |
| 1. Cell-To-Cell Signaling and Interaction | 7.34 x 10^-5^  to  2.18 x 10^-2^ | 19 | *AKAP12*,**CRYAB**,**VTN**,**IGKC**,**CD8A**,**VCAN**,C4A/**C4B**,**HMGB1**,**CTSD**,**SOD2**,**S100A9**,**APCS**,*RHOA*,**ANXA5**,**CAT**,**SERPINA1**,**IGHA1**,**A2M**,**PRDX2** |
| 1. Hematological System, Development and Function | 7.34 x 10^-5^  to  2.18 x 10^-2^ | 17 | **FTL**,**VTN**,**SERPINA3**,**IGHG1**,**CD8A**,C4A/**C4B**,**HMGB1**,*GNAI3*,**APOA1**,**S100A9**,**APCS**, **ANXA5**,*RHOA*,**SERPINA1**,**IGHA1**,**A2M**,**PRDX2** |
| 1. Inflammatory Response | 7.34 x 10^-5^  to  2.18 x 10^-2^ | 24 | **CA2**,**IGKC**,**VTN**,*TUBB2A*,**SERPINA3**,**IGHG1**,**GSN**,**HMGB1**,**COL1A2**,C4A/**C4B**,**CTSD**, *GNAI3*,**APOA1**,*TUBA1A*,**SOD2**,**S100A9**,**APCS**,*RHOA*,**ANXA5**,**CAT**,**SERPINA1**,**IGHA1**,**GSTP1**,**PRDX2** |
| 1. Cellular Assembly and Organization | 1.56 x 10^-4^  to  2.18 x 10^-2^ | 20 | **CRYAB**,*MAP2*,**VTN**,**IGHG1**,**GSN**,**VCAN**,**APOA1**,**SOD2**,*PHB*,**APCS**,**ANXA5**,*RHOA*,**CAT**, *OPA1*,RAB11A,**GFAP**,**A2M**,**ACTA1**,**IGHG2**,**HSPB1** |
| 1. Tissue Development | 1.56 x 10^-4^  to  2.18 x 10^-2^ | 18 | *AKAP12*,**CRYAB**,**VTN**,**GSN**,**CD8A**,**VCAN**,C4A/**C4B**,**SOD2**,**S100A9**,**APCS**,*RHOA*,**CAT**, **SERPINA1**,**A2M**,**ACTA1**,**HSPB1**,**PRDX2**,**FTH1** |
| 1. Embryonic Development | 1.58 x 10^-4^  to  1.65 x 10^-2^ | 12 | *DNM1*,**YWHAQ**,**CRYAB**,**SOD2**,**APOA1**,**SIRT2**,*RHOA*,**VTN**,*DLST*,*PEA15*,  **IGHG1**,**HSPB1** |
| 1. Renal and Urological System Development and Function | 1.58 x 10^-4^  to  2.02 x 10^-2^ | 6 | *DNM1*,**SOD2**,**APOA1**,*RHOA*,**VTN**,**RTN4** |
| 1. Gastrointestinal Disease | 2.34 x 10^-4^  to  2.18 x 10^-2^ | 22 | **GSTM1**,**CRYAB**,*CPT1A*,*TUBB2A*,*DCLK1*,**COL1A2**,**CTSD**,**HP**,**SOD2**,**APOA1**,*TUBA1A*, *UBQLN2*,**S100A9**,**PREPL**,*PHB*,*EFTUD2*,*RHOA*,**CAT**,**SERPINA1**,**GSTP1**,**FTH1**,**PRDX2** |
| 1. Cellular Compromise | 2.87 x 10^-4^  to  2.18 x 10^-2^ | 13 | **PRDX1**,**VTN**,**PRDX6**,*DNM1*,**SIRT2**,**SOD2**,**APCS**,**ANXA5**,*RHOA*,**CAT**,**RTN4**,*OPA1*, **IGHA1** |
| 1. Developmental Disorder | 3.8 x 10^-4^  to  2.15 x 10^-2^ | 30 | **CA2**,**FTL**,**CRYAB**,**ASS1**,*MT-CO2*,**LDHB**,C4A/**C4B**,**COL1A2**,**CTSD**,**SOD2**,**APCS**,*EFTUD2*,**SERPINA1**,**GFAP**,*KIF5C*, **ALDH6A1**,**ACTA1**,*SLC25A4*,*CPT1A*,**PGM1**,**GSN**,*NDUFS3*,*GNAI3*,**HP**,*TUBA1A*,**APOA1**,*NDUFV2*,*NDUFS8*,**CAT**,**FTH1** |
| 1. Renal and Urological Disease | 4.79 x 10^-4^  to  1.01 x 10^-2^ | 23 | *SLC4A4*,**S100A1**,**CA2**,**GSTM1**,**CRYAB**,**DBI**,*TUBB2A*,**PGM1**,*DPYSL5*,**HNRNPDL**,**GSN**, C4A/**C4B**,**HMGB1**,*TUBA1A*,**SOD2**,**HIST1H2BO**,**APCS**,**ANXA5**,*NEDD8*,*ATP5J2*,**GSTP1**,*HAGH*,**PRDX2** |
| 1. Infectious Disease | 4.89 x 10^-4^  to  1.69 x 10^-2^ | 27 | **CA2**,**CRYAB**,*GAK*,*CCT2*,*DCLK1*,**HMGB1**,C4A/**C4B**,**APCS**,**HIST1H2BO**,*DLST*,**SERPINA1**,*CAMK2B*,**S100A1**,*AP2M1*,**IGKC**,*TUBB2A*,**PGM1**,*DPYSL5*,**HNRNPDL**,*ATP6V0A1*,*DNM1*,*CRMP1*,**HP**,*ANXA6*,*TUBA1A*,**S100A9**,RAB11A |
| 1. Lipid Metabolism | 5.22 x 10^-4^  to  2.18 x 10^-2^ | 10 | **AKR1A1**,**APOA1**,**ANXA5**,*RHOA*,**CAT**,*OPA1*,**SERPINA1**,**PRDX6**,**GSTP1**,**PRDX2** |
| 1. Dermatological Diseases and Conditions | 6.26 x 10^-4^  to  2.01 x 10^-2^ | 21 | *DBN1*,**GSTM1**,**IGKC**,*TUBB2A*,**IGHG1**,**GSN**,**COL1A2**,C4A/**C4B**,**YWHAQ**,*PGD*,**APOA1**, *TUBA1A*,**SOD2**,*PHB*,**S100A9**,**ANXA5**,**LGALS3BP**,**CAT**,**SERPINA1**,**A2M**,**GSTP1** |
| 1. Cell Cycle | 7.44 x 10^-4^  to  1.46 x 10^-2^ | 7 | **HMGB1**,*AKAP12*,*GNAI3*,*TUBA1A*,**SIRT2**,*RHOA*,**CAT** |
| 1. Post-Translational Modificationion | 7.79 x 10^-4^  to  7.31 x 10^-3^ | 4 | **CTSD**,**FTL**,**APCS**,**FTH1** |
| 1. Protein Degradation | 7.79 x 10^-4^  to  7.31 x 10^-3^ | 4 | **CTSD**,**FTL**,**APCS**,**FTH1** |
| 1. Nervous System Development and Function | 1.09 x 10^-3^  to  7.31 x 10^-3^ | 3 | *RHOA*,**RTN4**,**HSPB1** |
| 1. Tissue Morphology | 1.09 x 10^-3^  to  2.18 x 10^-2^ | 3 | **GSTM1**,*RHOA*,**RTN4** |
| 1. Immune Cell Trafficking | 1.14 x 10^-3^  to  2.18 x 10^-2^ | 9 | C4A/**C4B**,**HMGB1**,*GNAI3*,**APOA1**,**S100A9**,*RHOA*,**SERPINA1**,**SERPINA3**,**PRDX2** |
| 1. Hepatic System Disease | 1.16 x 10^-3^  to  2.15 x 10^-2^ | 5 | *CPT1A*,**SOD2**,**CAT**,**SERPINA1**,**GSTP1** |
| 1. Drug Metabolism | 1.84 x 10^-3^  to  1.46 x 10^-2^ | 5 | **GSTM1**,**VTN**,**CAT**,**GSTP1**,**FTH1** |
| 1. Organismal Development | 2.29 x 10^-3^  to  2.18 x 10^-2^ | 6 | **GSTM1**,**CRYAB**,**SIRT2**,**APCS**,*RHOA*,**IGHG1** |
| 1. Reproductive System Disease | 2.69 x 10^-3^  to  2.01 x 10^-2^ | 33 | **CA2**,**CRYAB**,**ASS1**,*CCT2*,**VCAN**,C4A/**C4B**,**YWHAQ**,**COL1A2**,**CTSD**,**MFI2**,*SLC25A6*,  **SOD2**,*PHB*,*EFTUD2*,**ANXA5**,*ATP5J2*,**SERPINA1**,**PSAT1**,**GSTM1**,*SLC25A4*,**IGKC**,  *TUBB2A*,**GSN**,*CRMP1*,**HP**,*TUBA1A*,**APOA1**,*NRCAM*,**S100A9**,*RHOA*,*PEA15*,*HAGH*,  **GSTP1** |
| 1. Respiratory System Development and Function | 2.79 x 10^-3^  to  7.31 x 10^-3^ | 3 | **HMGB1**,**VTN**,**SERPINA1** |
| 1. Tumor Morphology | 2.79 x 10^-3^  to  2.18 x 10^-2^ | 3 | **COL1A2**,**VTN**,**GSTP1** |
| 1. Hematological Disease | 4.55 x 10^-3^  to  2.18 x 10^-2^ | 23 | **CA2**,**GSTM1**,**FTL**,**CRYAB**,*CPT1A*,**IGKC**,*TUBB2A*,*MT-CO2*, **GSN**, **VCAN**, C4A/**C4B**, **COL1A2**,**CTSD**,**HP**,**APOA1**,*TUBA1A*,*ANXA6*,**SOD2**,*RHOA*,**GFAP**,**A2M**,**GSTP1**,**FTH1** |
| 1. Hair and Skin Development and Function | 5.19 x 10^-3^  to  1.65 x 10^-2^ | 4 | *DNM1*,**APOA1**,*RHOA*,**VTN** |
| 1. Cardiovascular Disease | 5.23 x 10^-3^  to  2.01 x 10^-2^ | 20 | **GSTM1**,**CA2**,**CRYAB**,*SLC25A4*,*GLS*,*TUBB2A*,**GSN**,C4A/**C4B**,**COL1A2**,**APOA1**,*TUBA1A*, **SOD2**,**S100A9**,**APCS**,**ANXA5**,*EFTUD2*,**RTN4**,*SLC1A2*,**GSTP1**,**FTH1** |
| 1. Respiratory Disease | 5.23 x 10^-3^  to  1.65 x 10^-2^ | 5 | **GSTM1**,*TUBA1A*,*TUBB2A*,**SERPINA1**,**GSTP1** |
| 1. Connective Tissue Development and Function | 5.79 x 10^-3^  to  1.46 x 10^-2^ | 5 | **SOD2**,*RHOA*,**VTN**,**VCAN**,**FTH1** |
| 1. Vitamin and Mineral Metabolism | 5.94 x 10^-3^  to  1.54 x 10^-2^ | 5 | **APOA1**,*RHOA*,*OPA1*,**SERPINA1**,**FTH1** |
| 1. DNA Replication, Recombination, and Repair | 6 x 10^-3^  to  2.02 x 10^-2^ | 9 | **HMGB1**,**CTSD**,**SOD2**,**EXOG**,**CAT**,*OPA1*,**GSN**,**GSTP1**, **FTH1** |
| 1. Amino Acid Metabolism | 7.31 x 10^-3^  to  1.46 x 10^-2^ | 2 | *SLC1A2*,**FTH1** |
| 1. Carbohydrate Metabolism | 7.31 x 10^-3^  to  1.46 x 10^-2^ | 9 | **CRYAB**,*PGD*,**APOA1**,**VTN**,*RHOA*,**ANXA5**,**CAT**,**PRDX6**,**PRDX2** |
| 1. Cardiovascular System Development and Function | 7.31 x 10^-3^  to  2.18 x 10^-2^ | 8 | **S100A1**,**GSTM1**,**CRYAB**,**VTN**,*RHOA*,**RTN4**,**IGHG1**,**HSPB1** |
| 1. Endocrine System Development and Function | 7.31 x 10^-3^  to  1.46 x 10^-2^ | 2 | **VTN**,**GSTP1** |
| 1. Energy Production | 7.31 x 10^-3^  to  7.31 x 10^-3^ | 1 | **CRYAB** |
| 1. Nucleic Acid Metabolism | 7.31 x 10^-3^  to  1.46 x 10^-2^ | 9 | *AKAP12*,*CRMP1*,*PGD*,**APOA1**,*PSMC6*,*ATP5J2*,*OPA1*,*CMPK1*,*NUDT2* |
| 1. Nutritional Disease | 7.31 x 10^-3^  to  7.31 x 10^-3^ | 1 | **FTH1** |
| 1. Organ Development | 7.31 x 10^-3^  to  7.31 x 10^-3^ | 1 | **SIRT2** |
| 1. Protein Synthesis | 7.31 x 10^-3^  to  1.37 x 10^-2^ | 8 | **CTSD**,**GSTM1**,**CRYAB**,*ANXA6*,**SOD2**,**APCS**,**CAT**,**GSTP1** |
| 1. Reproductive System Development and Function | 7.31 x 10^-3^  to  7.31 x 10^-3^ | 1 | **SIRT2** |
| 1. Skeletal and Muscular System Development and Function | 7.31 x 10^-3^  to  1.46 x 10^-2^ | 4 | **S100A1**,**CRYAB**,**VTN**,*RHOA* |
| 1. Hematopoiesis | 8.35 x 10^-3^  to  1.46 x 10^-2^ | 3 | C4A/**C4B**,**HMGB1**,**APCS** |
| 1. Behavior | 9.23 x 10^-3^  to  1.46 x 10^-2^ | 4 | *GNAI3*,**SOD2**,*NRCAM*,*DLG4* |
| 1. Cell-mediated Immune Response | 1.46 x 10^-2^  to  1.46 x 10^-2^ | 1 | **HMGB1** |
| 1. Humoral Immune Response | 1.46 x 10^-2^  to  1.46 x 10^-2^ | 1 | **HMGB1** |
| 1. Lymphoid Tissue Structure and Development | 1.46 x 10^-2^  to  1.46 x 10^-2^ | 1 | **HMGB1** |
| 1. Organ Morphology | 1.46 x 10^-2^  to  1.46 x 10^-2^ | 1 | **S100A1** |
| 1. Visual System Development and Function | 1.46 x 10^-2^  to  1.46 x 10^-2^ | 1 | **IGHG1** |
| 1. Gene Expression | 2.03 x 10^-2^  to  2.03 x 10^-2^ | 7 | **HMGB1**,**HP**,**SOD2**,*PHB*,**PRDX1**,**CAT**,**GSTP1** |
